# Synthesis of Potential Antiviral Agents for SARS-CoV-2 Using Molecular Hybridization Approach

**DOI:** 10.3390/molecules27185923

**Published:** 2022-09-12

**Authors:** Kailey A. Wyman, Adel S. Girgis, Pragnakiran S. Surapaneni, Jade M. Moore, Noura M. Abo Shama, Sara H. Mahmoud, Ahmed Mostafa, Reham F. Barghash, Zou Juan, Radha D. Dobaria, Ahmad J. Almalki, Tarek S. Ibrahim, Siva S. Panda

**Affiliations:** 1 Department of Chemistry and Physics, Augusta University, Augusta, GA 30912, USA; 2 Department of Pesticide Chemistry, National Research Centre, Dokki, Giza 12622, Egypt; 3 Schulich School of Medicine and Dentistry, Western University, London, ON N6A5C1.1, Canada; 4 Center of Scientific Excellence for Influenza Viruses, National Research Centre, Giza 12622, Egypt; 5 Department of Pharmaceutical Chemistry, Faculty of Pharmacy, King Abdulaziz University, Jeddah 21589, Saudi Arabia

**Keywords:** quinoline, indole, rhodanine, SARS-CoV-2, molecular modeling, healthcare

## Abstract

We synthesized a set of small molecules using a molecular hybridization approach with good yields. The antiviral properties of the synthesized conjugates against the SAR-CoV-2 virus were investigated and their cytotoxicity was also determined. Among all the synthesized conjugates, compound **9f** showed potential against SARS-CoV-2 and low cytotoxicity. The conjugates’ selectivity indexes (SIs) were determined to correlate the antiviral properties and cytotoxicity. The observed biological data were further validated using computational studies.

## 1. Introduction

SARS-CoV-2 is a virulent strain of the *Coronaviridae* family probably originating primarily from bats that was first transmitted through an unknown intermediate host to humans and found in Wuhan, China [1]. This virus family mainly infects mammals, such as camels, cats, and cattle [2]. However, strains with specific genome mutations can infect humans [3]. Furthermore, when the virus began to spread across the globe, it developed multiple genotypes varying in the level of contagiousness, as well as the severity of symptoms. The delta and omicron variants have become the most prevalent strains found in those who have contracted the virus [1]. Currently, there are three ways to describe the variants: a variant of interest, a variant of concern, and a variant of high consequence [1]. A variant of interest refers to a strain of the virus that differs from the original. A concern variant is a strain with high infectability that can cause breakthrough infections. Finally, a variant of high consequence is a strain that causes recurrent cases or is not prevented by current vaccines [1]. There are no current strains that cannot be prevented through the administration of one of the vaccines; however developing more treatment options is important before resistance becomes the case [3].

**Figure 1:**
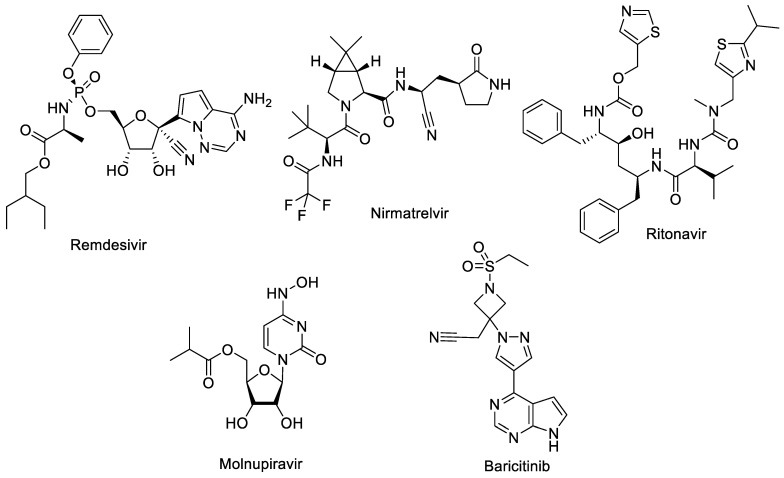
FDA-approved anti-SARS-CoV-2 drugs.

The current antiviral treatments exclusively for SARS-CoV-2 are limited. However, the use of alternative drug compounds has been given emergency approval by the FDA in order to offer additional treatment options. This process is called drug repurposing and it involves using a drug to treat a disease or illness when it has been approved and designed for a different disease [3]. In trying to find a treatment as quickly as possible for SARS-CoV-2, repurposing has been the main way of offering treatments and gaining data on possible drugs that naturally possess efficacy. Currently, there are four treatments used for SARS-CoV-2 patients; however, they are only used when the patient is in a dire condition. Critical patients are usually placed on a ventilator or on a high volume of supplementary oxygen, and they often have other serious health conditions [4]. One of the first drugs to receive emergency approval was Veklury (Remdesivir), originally used as an injectable broad-spectrum antiviral [5]. Its use was approved because it is believed that it behaves like an analog of adenosine triphosphate (ATP) and competes with it to incorporate into viral DNA [4]. This is thought to disrupt the process of DNA replication for SARS-CoV-2, thus preventing subsequent RNA replication [4]. The next drug that was approved is an oral antiviral known as Paxlovid, developed by Pfizer, which is comprised of two separate drugs. The first is Nirmatrelvir, which blocks a specific enzyme required for SARS-CoV-2 DNA replication. The second is called Ritonavir, which prevents the metabolic breakdown of Nirmatrelvir [4]. Another approved oral antiviral is Molnupiravir, developed by Merck, which has shown efficacy in severe SARS-CoV-2 cases. The last drug approved is Oluminant (Baricitnib), developed by Lilly [4]. This is an anti-inflammatory drug used for rheumatoid arthritis (inhibitor of Janus kinases (JAK1 and JAK2)) and it is not proven to directly affect the SARS-CoV-2 virus. Its efficacy, in some cases, is due to the decrease in inflammation it causes, which can help give the body the chance to heal affected structures, including the respiratory system. While there are current treatments that are specific to SARS-CoV-2, toxicity has become a significant concern with the use of the new drugs (Figure 1).

Developing new drugs has always been time-consuming, expensive, tedious, and challenging. In the current situation, molecular hybridization of bioactive scaffolds could be a powerful and attractive rational drug design strategy for the development of new potential drug candidates due to several advantages, such as (a) achieving selectivity, (b) gaining desired activity, (c) multiple pharmacological targets, and (d) lower possible cytotoxicity. We aimed to develop potential drug candidates for use with biologically important quinolone, indole, and rhodamine scaffolds.

The quinolinyl skeleton from the repurposed drugs (chloroquine and hydroxychloroquine) and triazolyl heterocycle was considered because of its key role in the drug development process and well-established, diverse pharmacological properties [6,7,8,9,10]. Insertion of the fluorine atom in the quinolinyl heterocycle is a bioisosteric approach and, due to its electronegative properties, it may alter the physicochemical properties of the designed molecules [11]. The use of indoles in the designed drug candidates could be highly effective and crucial due to how they function in biological systems. Indoles typically act as mimics of amino acids and reversibly bind to enzymes, usually causing inhibition [12]. The FDA-approved drug Arbidol is useful as a broad-spectrum antiviral, and Delavirdine is a first-generation reverse transcriptase inhibitor for HIV-1 viral strains [13]. In addition, rhodanine is an important scaffold for the development of potential antiviral drug candidates [14,15,16].

## 2. Results and Discussion

**Figure 2:**
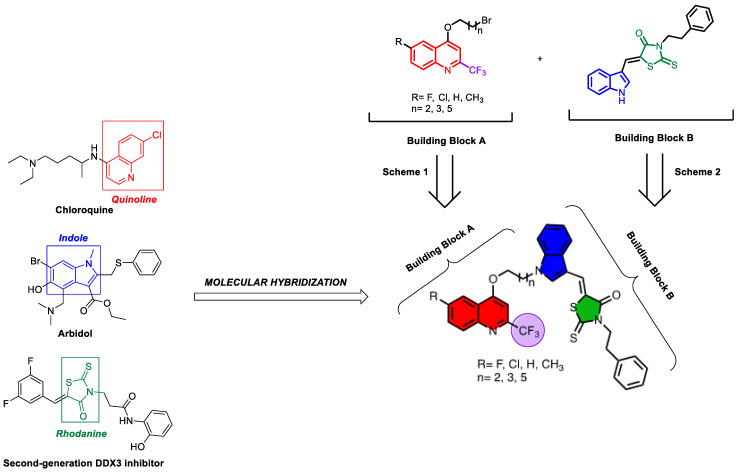
Proposed compounds for molecular hybridization.

Molecular hybridization (conjugation of two or more antiviral molecules via a covalent bond) is an effective and efficient tool for the development of new drug candidates, such as anti-SARS-CoV-2 drugs. Furthermore, the molecular hybrids could also have the advantages of overcoming drug resistance, lowering the risk of drug–drug interactions, being cost-effective, having a synergistic effect, exhibiting dual activity, and minimizing redundant side effects. Concerning the importance of medicinally interesting scaffolds (**quinoline**, **indole**, and **rhodanine**), we utilized a hybridized molecular approach and proposed several targets (Figure 2). The fluorine in the targeted molecules was introduced due to its unique properties. The high electronegativity of fluorine can enhance lipophilicity and significantly alter the physicochemical properties (such as solubility or the logP) of a molecule predictably. In addition, the incorporation of fluorine may improve the stability and efficacy while reducing the cytotoxicity of our designed molecules.

**Scheme 1:**
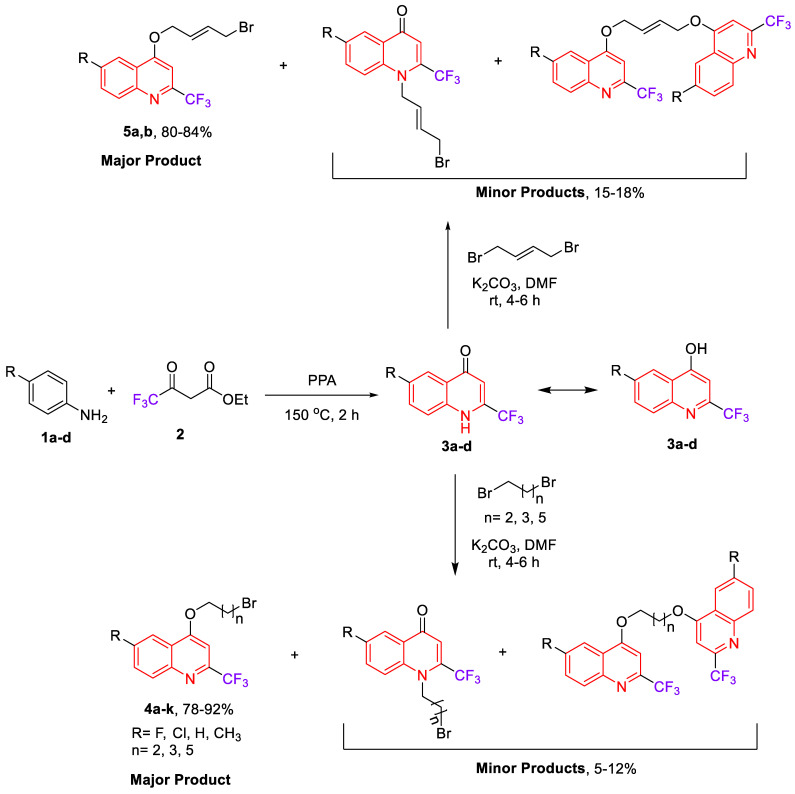
Synthesis of building block A.

**Scheme 2:**
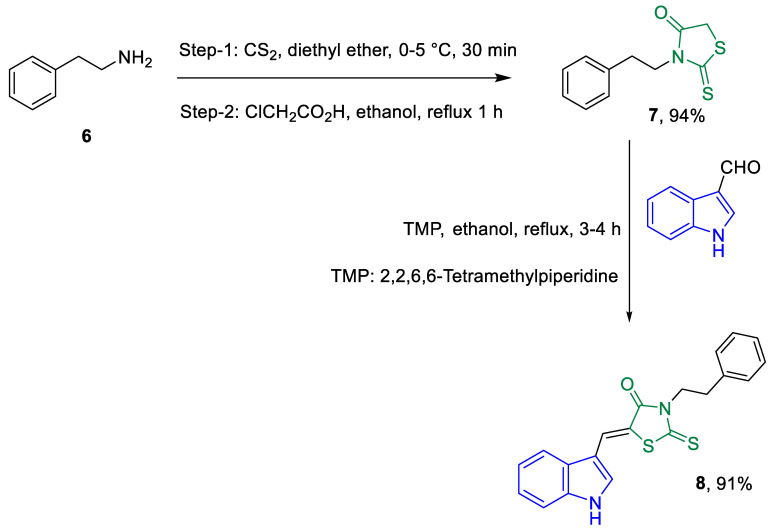
Synthesis of building block B.

**Scheme 3:**
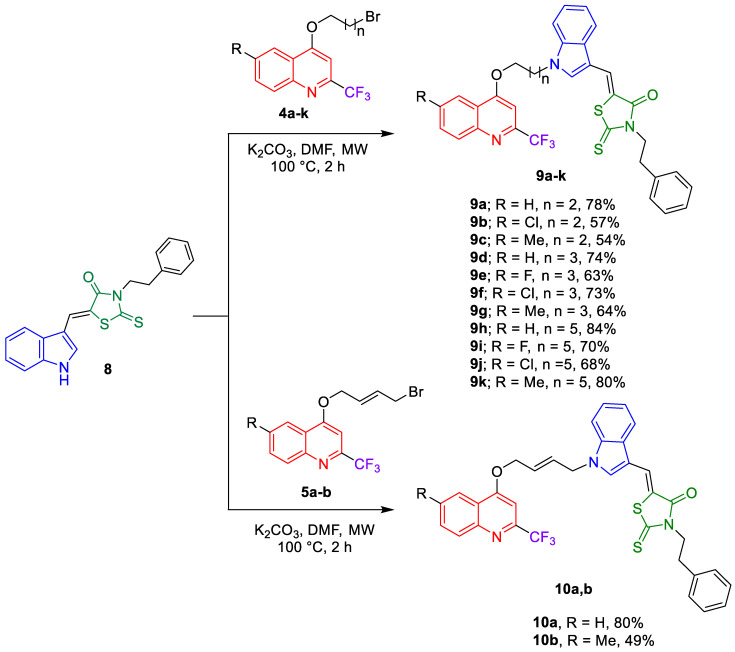
Synthesis of molecular hybrid conjugates **9a–k** and **10a,b**.

The synthetic routes designed and employed to obtain the proposed molecular hybrid conjugates **9** and **10** are represented in Scheme 1, Scheme 2 and Scheme 3. To prepare building block A, the synthesis of 6-substituted-2-(trifuoromethyl)quinoline-4(1*H*)-ones **3a–d** was initially carried out using Conrad–Limpach cyclo-condensation. The quinolones formed were further alkylated with dibromoalkanes/dibromobutene in the presence of K_2_CO_3_. The reaction resulted in a mixture of the major *O*-alkylated quinolines **4**/**5** and the minor *N*-alkylated quinolones, along with bis-derivatives, as illustrated in Scheme 1 [17,18,19,20]. All the compounds in the mixture were separated by column chromatography and the structures were confirmed by spectroscopic studies (Supplementary Materials).

Building block B was prepared as described in Scheme 2. Phenethyl-2-thioxothiazolidin-4-one **7** was synthesized through treatment of phenylethylamine **6** with CS_2_ in diethyl ether (0–5 °C. 30 min), followed by heating the precipitated solid in ethanol with chloroacetic acid for 1 h, which gave **7** in an 85% yield. Further, compound **7** was refluxed with indole-3-aldehyde in ethanol in the presence of tetramethylpiperidine (TMP) for 3–4 h to obtain **8** in a good yield (Scheme 2).

Finally, building block A was coupled with building block B in DMF in the presence of K_2_CO_3_ under microwave irradiation at 100 °C for 2 h to yield the desired hybrid conjugates **9a–k** and **10a**,**b** (Scheme 3). This step was also carried out under conventional heating conditions, but we found that microwave irradiation conditions produced the desired molecules in good yields and with good purity. All the synthesized hybrid conjugates were characterized with spectral studies. The overall yields for the synthesis of the desired final products ranged from 48–61%. We also scaled up the reaction to a 5 g scale, which worked as described above without any roadblocks.

### 2.1. HPLC-MS Analysis

**Figure 3:**
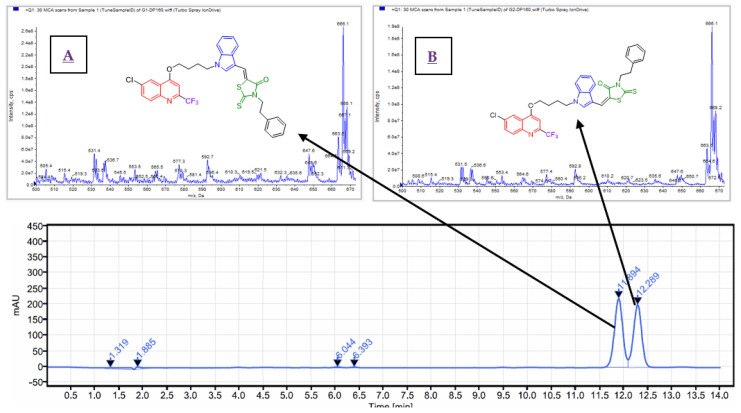
HPLC–MS spectra of compound **9f**. Figure (**A**,**B**) are mass spectra of two isomers (*E*/*Z*) observed in HPLC.

Since we had a double bond in our final products, we were expecting the formation of possible *E* and *Z* isomers, but we could not visualize them in TLC analysis. Furthermore, we were not able to see, or it was difficult to identify, the duplication of peaks in both the proton and carbon, probably due to the double bond attached to the thioxothiazolidinone ring. Liquid chromatography was performed to confirm the presence of *E* and *Z* isomers. As expected, we observed the presence of *E* and *Z* isomers in the HPLC-MS analysis of the final products. We adopted the HPLC gradient method with two solvents, water with 0.1% formic acid and acetonitrile, and injected a 5 µL sample into the system. The HPLC spectrum showed two close-by peaks, which indicated the possible formation of *E* and *Z* isomers. Further mass analysis of both peaks confirmed the *E* and *Z* isomers, as both peaks had the same molecular weights (Figure 3).

### 2.2. Antiviral Studies

**Figure 4:**
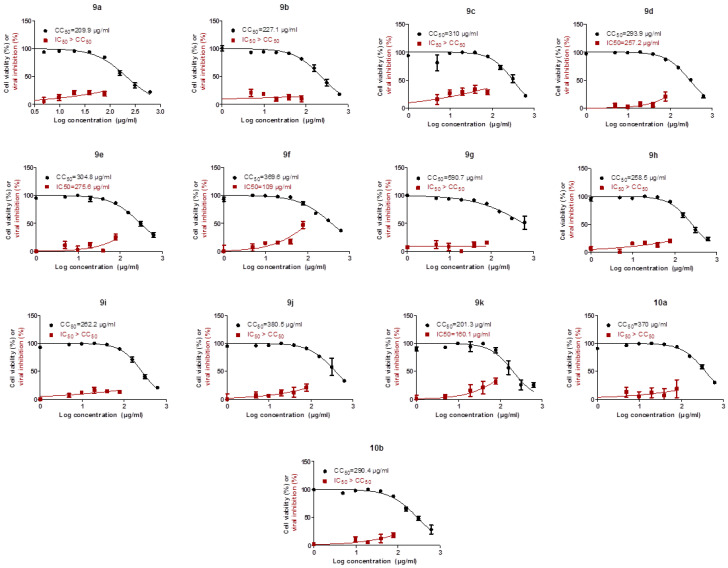
Dose–response curves for the tested anti-SARS-CoV-2compounds (**9a–k** and **10a,b**).

The antiviral properties of the synthesized conjugates (**9a–k**, and **10a**,**b**) against SARS-CoV-2 were determined with the standard VERO-E6 (normal *Clorocebus aethiops* kidney cells) technique [21,22,23,24] (Table 1, Figure 4). It was noted that some of the synthesized agents showed promising anti-SARS-CoV-2 properties with reasonable selectivity indexes (SIs, the ratio of CC_50_ relative to IC_50_). Conjugate **9f** (n = 3, R = Cl) was the most effective agent synthesized, revealing potent antiviral inhibitory properties with a promising selectivity index (IC_50_ = 163.6 µM, CC_50_ = 554.8 µM, SI = 3.4). Conjugate **9k** (n = 5, R = Me) also revealed comparable anti-SARS-CoV-2 properties, with a lower selectivity index (IC_50_ = 237.6 µM, CC_50_ = 298.7 µM, SI = 1.3) compared to that of **9f**. Conjugates **9d** and **9e** showed mild selectivity indexes (SI = 1.1) due to the CC_50_ being close to their IC_50_ values (IC_50_ = 407.1, 424.2, CC_50_ = 465.2, 469.1 µM for **9d** and **9e**, respectively). Based on the observed data, it can be concluded that the alkyl linkage with four carbon atoms connecting the quinolinyl and indolyl heterocycle was the most appropriate for optimizing anti-SARS-CoV-2 agents. Although none of the synthesized conjugates revealed a potency comparable to hydroxychloroquine or remdesivir (standard references) [23], they were considered promising hits that can be manipulated for optimizing good hits/leads against SARS-CoV-2. To better understand the controlling parameters behind bio-observations, and also rationalize the promising anti-SARS-CoV-2 properties of conjugate **9f**, computational studies were undertaken in the form of docking studies in PDB ID: 6LU7, which is the main SARS-CoV-2 protease (M^pro^) [25,26].

### 2.3. Docking Studies

**Figure 5:**
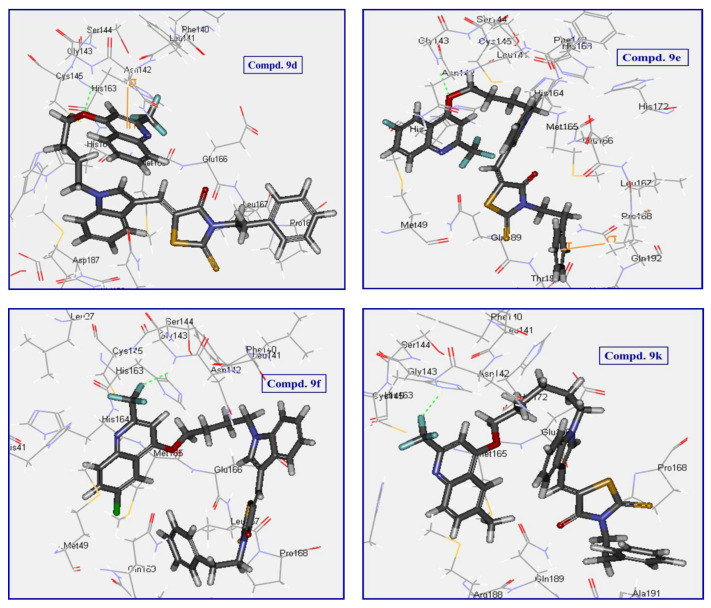
Docking poses of the tested compounds (**9d**, **9e**, **9f**, and **9k**) in PDB ID: 6LU7.

Computational techniques (either ligand- or structure-based techniques) are helpful in medicinal chemical studies and can determine the parameters necessary for bio-properties [28,29]. To understand the reason(s) behind the bio-properties of the synthesized agents (**9d–f,k**) docking studies were undertaken using the standard CDOCKER technique (Discovery Studio 2.5 software) in PDB ID: 6LU7 [24]. Table 2 shows the docking results, including CDOCKER interaction energy scores (Kcal mol^−1^) and the interaction(s) (including hydrogen bonding and π interactions) that took place between the docked agent and the protein active site (Figure 5). The RMS gradient (0.098) validated the docking observations. It was noticed that all the tested agents demonstrated hydrogen bonding with GLY143, which is one of the amino acids of the protein active site that enables hydrogen bonding interactions with the co-crystallized ligand “N3 inhibitor” of PDB ID: 6LU7 [24]. The observed interaction docking score values were comparable with the IC_50_ of the tested agents (interaction docking scores = −50.9, −49.4, −51.8, −50.7 Kcal/mol; IC_50_ = 407.1, 424.2, 163.6, 237.6 µM, for compounds **9d–f,k** respectively). The slight deviations in these values can be attributed to the differences between the computational and biological techniques.

### 2.4. Absorption, Distribution, Metabolism, and Excretion (ADME) Studies

Computed ADME descriptors were determined with the standard technique (force field: CHARMM, partial charge: Momany-Rone) in Discovery Studio 2.5 software [30]. Table 3 shows the most important ADME descriptors of the synthesized agents with anti-SARS-CoV-2 properties. It was noticed that all the agents tested had good aqueous solubility (considering that the solubility levels were 0 = extremely low, 1 = very low, 2 = low, 3 = good, and 4 = optimal). Good intestinal absorptions were also noticed for all the tested conjugates (considering that the intestinal solubility levels were 0 = good, 1 = moderate, and 2 = poor). The plasma protein binding (PPB) level was > 95% (considering that the PPB levels were 0 = < 90%, 1 = > 90%, 2 = > 95%). From all the above, it can be concluded that the synthesized agents, especially **9f**, can be considered for optimizing hits/leads with high efficacy against SARS-CoV-2.

## 3. Conclusions

In conclusion, using an optimized facile reaction condition, we synthesized a set of quinolone-, indole-, and rhodanine-incorporated molecular hybrids in good yields. Among all the synthesized conjugates, compound **9f** showed promising antiviral activity against SARS-CoV-2. The selectivity indexes (SIs) of the conjugates and the molecular docking studies data highlight the potential for further investigation and indicate that the findings can be used as a resource for developing potential antiviral drug candidates, considering **9f** as a lead molecule. HPLC-MS studies confirmed the presence of both *E* and *Z* isomers in the final products. This could be interesting for further investigations of the antiviral properties of both isomers separately through the development of an advanced analytical methodology.

## 4. Experimental Section

Melting points were determined on a capillary point apparatus equipped with a digital thermometer. NMR spectra were recorded in DMSO-*d_6_* on a Bruker spectrometer operating at 500 MHz for ^1^H (with TMS as an internal standard) and 125 MHz for ^13^C using the NMR facility at the Department of Chemistry and Physics, Augusta University, Augusta, GA, USA. IR spectra (KBr, cm^−1^) were recorded on a Nicolet iS5 spectrophotometer (Thermo Fisher Scientific, Waltham, MA, USA) at the Department of Chemistry and Physics, Augusta University, Augusta, GA, USA. MS was measured using an Agilent Technologies 6545 Q-TOF LC/MS. TLC was performed on pre-coated silica gel (Merck 60 F254); spots were visualized by iodine vapors or irradiation with UV light (254 nm). High-Pressure Liquid Chromatography (HPLC) Agilent 1100 Series with a 1260 Infinity II LC was used to obtain the HPLC-MS data. All microwave-assisted reactions were carried out with a single-mode cavity Discover Microwave Synthesizer (CEM Corporation, Matthews, NC, USA). The reaction mixtures were transferred into a 10 mL glass pressure microwave tube equipped with a magnetic stirrer bar. The tube was closed with a silicon septum and the reaction mixture was subjected to microwave irradiation (Discover mode; run time: 60 s; Power Max cooling mode).

### 4.1. General Procedure for the Synthesis of Building Block A

All the derivatives of building block A were prepared according to our previously reported method [18,19].

### 4.2. General Procedure for the Synthesis of Building Block B

Using the synthesized compound **7** (12.56 g, 53 mmol, 1 eq.) [15], an equimolar amount of indole-3-carboxaldehyde was added to a 100 mL round bottom flask (RBF). Then, 14 drops of 2,2,6,6-tetramethylpiperidine were added to the flask followed by 50 mL ethanol. The reaction was heated to reflux for 3–4 h while stirring. The separated solid upon cooling the reaction mixture (20–30 min.) was collected and washed with minimal ethanol. The obtained solid was purified by flash chromatography.

(E/Z)-5-((1H-indol-3-yl)methylene)-3-phenethyl-2-thioxothiazolidin-4-one (**8**)

Light yellow solid, mp: 165–167 °C, yield: 91%. ^1^H NMR δ: 8.81 (bs, 1H), 8.10 (s, 1H), 7.88 (d, *J* = 7.3 Hz, 1H), 7.53 (d, *J* = 3.1 Hz, 1H), 7.46 (d, *J* = 9.8 Hz, 1H), 7.36–7.31 (m, 6H), 4.38–4.34 (m, 2H), 3.05–3.01 (m, 2H); ^13^C NMR δ: 184.7, 171.9, 137.7, 129.0, 128.6, 127.6, 126.8, 122.2, 118.9, 111.8, 45.8, 33.1; LC-MS m/z for C20H16N2OS2 [M+H]^+^ Calcd. 365.07. Found. 365.10.

### 4.3. General Procedure for the Synthesis of Molecular Hybrid Conjugates ***9a–k*** and ***10a,b***

Equimolar amounts of building block A (0.26 mmol, 1 eq.) and building block B were mixed in a microwave tube with a stir bar with K_2_CO_3_ (0.78 mmol, 3 eq.) followed by DMF (3.4 mL). The reaction was irradiated by microwave for 2 h at 100 °C. After completion, the reaction mixture was poured over ice water. The product was extracted with ethyl acetate and brine and dried over anhydrous sodium sulfate. The solid obtained upon evaporating the solvent under reduced pressure was recrystallized from aqueous ethanol giving the corresponding **9a–k** and **10a,b**.

(E/Z)-3-Phenethyl-2-thioxo-5-((1-(3-((2-(trifluoromethyl)quinolin-4-yl)oxy)propyl)-1H-indol-3-yl)methylene)thiazolidin-4-one (**9a**)

Yellow solid, mp: 185–187 °C, yield: 78%. IR: ν_max_/cm^−1^ 3076, 2950, 1706, 1614, 1590, 1375, 1189, 1111; ^1^H NMR δ: 8.05–7.82 (m, 6H), 7.70 (t, *J* = 9.5 Hz, 1H), 7.61 (t, *J* = 7.0 Hz, 1H), 7.31–7.15 (m, 8H), 4.67 (t, *J* = 7.4 Hz, 2H), 4.41 (t, *J* = 5.7, 2H), 4.20 (t, *J* = 7.8 Hz, 2H), 2.93 (t, *J* = 7.8 Hz, 2H), 2.47–2.51 (m, 2H); ^13^C NMR δ: 192.0, 166.3, 162.7, 147.3, 137.8, 136.3, 133.3, 131.3, 129.0, 128.7, 128.6, 127.7, 127.6, 126.7, 125.3, 123.5, 122.0, 121.9, 121.0, 118.9, 114.5, 111.1, 110.6, 97.3, 67.1, 45.2, 44.1, 32.2, 28.5; LC-MS m/z for C33H26F3N3O2S2 [M+H]^+^ Calcd. 618.14. Found. 618.10.

(E/Z)-5-((1-(3-((6-Chloro-2-(trifluoromethyl)quinolin-4-yl)oxy)propyl)-1H-indol-3-yl)methylene)-3-phenethyl-2-thioxothiazolidin-4-one (**9b**)

Yellow solid, mp: 176–179 °C, yield: 57%. IR: ν_max_/cm^−1^ 3020, 2951, 2924, 1699, 1594, 1519, 1365, 1344, 1120. ^1^H NMR δ: 7.95 (d, *J* = 9.0 Hz, 1H), 7.88 (s, 1H), 7.85 (d, *J* = 7.8 Hz, 1H), 7.83 (s, 1H), 7.76–7.74 (m, 1H), 7.67 (d, *J* = 8.1 Hz, 1H), 7.47 (d, *J* = 2.5 Hz, 1H), 7.31–7.17 (m, 8H), 4.65 (t, *J* = 6.3 Hz, 2H), 4.46 (t, *J* = 5.4 Hz, 2H), 4.17 (t, *J* = 7.9 Hz, 2H), 2.92–2.88 (m, 2H), 2.47–2.42 (m, 2H). ^13^C NMR δ: 191.9, 166.3, 161.9, 145.8, 137.8, 136.3, 132.6, 131.8, 131.0, 128.7, 128.7, 127.5, 126.7, 121.9, 120.8, 114.2, 110.6, 98.2, 68.3, 45.1, 44.8, 32.3, 28.3. LC-MS m/z for C_33_H_25_ClF_3_N_3_O_2_S_2_ [M+H]^+^ Calcd. 652.10. Found. 652.20.

(E/Z)-5-((1-(3-((6-Methyl-2-(trifluoromethyl)quinolin-4-yl)oxy)propyl)-1H-indol-3-yl)methylene)-3-phenethyl-2-thioxothiazolidin-4-one (**9c**)

Yellow solid, mp: 182–184 °C, yield: 54%. IR: ν_max_/cm^−1^ 3059, 2933, 1705, 1590, 1575, 1516, 1280, 1181, 1164, 1150. ^1^H NMR δ: 7.93–7.85 (m, 4H), 7.69 (d, *J* = 8.2 Hz, 1H), 7.62 (d, *J* = 8.7 Hz, 1H), 7.47 (s, 1H), 7.31–7.23 (m, 8H), 4.67 (s, 2H), 4.42 (s, 2H), 4.19 (t, *J* = 7.9 Hz, 2H), 3.17 (d, *J* = 5.2 Hz, 1H), 2.93 (t, *J* = 8.0 Hz, 2H), 2.44 (s, 4H). ^13^C NMR δ: 191.9, 166.2, 162.0, 145.9, 137.7, 137.5, 136.2, 133.4, 128.6, 128.5, 127.5, 126.6, 125.1, 123.4, 121.8, 120.9, 120.4, 118.8, 114.3, 111.0, 110.5, 97.1, 67.4, 48.6, 45.1, 44.5, 32.2, 28.3, 21.4. LC-MS m/z for C_34_H_28_F_3_N_3_O_2_S_2_ [M+H]^+^ Calcd. 632.16. Found. 632.10.

(E/Z)-3-Phenethyl-2-thioxo-5-((1-(4-((2-(trifluoromethyl)quinolin-4-yl)oxy)butyl)-1H-indol-3-yl)methylene)thiazolidin-4-one (**9d**)

Yellow solid, mp: 157–159 °C, yield: 74%. IR: ν_max_/cm^−1^ 3059, 2961, 2937, 1698, 1592, 1374, 1171, 1157, 1131, 1113 (C-F). ^1^H NMR δ: 8.11 (d, *J* = 8.4 Hz, 1H), 8.04 (d, *J* = 8.3 Hz, 1H), 8.00 (s, 1H), 7.97 (s, 1H), 7.92 (d, *J* = 7.3 Hz, 1H), 7.86–7.83 (m, 1H), 7.68–7.65 (m, 2H), 7.32–7.21 (m, 8H), 4.42 (t, *J* = 7.0 Hz, 2H), 4.35 (t, *J* = 6.3 Hz, 2H), 4.24–4.20 (m, 2H), 2.95 (t, *J* = 7.8 Hz, 2H), 2.11–2.08 (m, 2H), 1.90–1.87 (m, 2H). ^13^C NMR δ: 192.1, 166.4, 162.8, 147.4, 137.7, 136.3, 133.1, 131.4, 129.1, 128.7, 128.6, 128.0, 127.4, 126.6, 125.4, 123.5, 121.9, 121.7, 121.2, 118.8, 114.5, 111.2, 110.4, 97.4, 68.6, 46.1, 45.2, 32.2, 26.0, 25.5. LC-MS m/z for C_34_H_28_F_3_N_3_O_2_S_2_ [M+H]^+^ Calcd. 632.16. Found. 632.10.

(E/Z)-5-((1-(4-((6-Fluoro-2-(trifluoromethyl)quinolin-4-yl)oxy)butyl)-1H-indol-3-yl)methylene)-3-phenethyl-2-thioxothiazolidin-4-one (**9e**)

Yellow solid, mp: 160–162 °C, yield: 63%. IR: ν_max_/cm^−1^ 2937, 2974, 1698, 1597, 1517, 1478, 1187, 1169, 1139. ^1^H NMR δ: 8.14–8.11 (m, 1H), 7.99 (s, 1H), 7.93 (s, 1H), 7.90 (d, *J* = 7.9 Hz, 1H), 7.78–7.74 (m, 2H), 7.68 (d, *J* = 8.3 Hz, 1H), 7.31–7.28 (m, 4H), 7.23–7.20 (m, 4H), 4.47 (t, *J* = 7.0 Hz, 2H), 4.34 (t, *J* = 6.2 Hz, 2H), 4.22 (t, *J* = 7.8 Hz, 2H), 2.96 (t, *J* = 7.9 Hz, 2H), 2.12–2.09 (m, 2H), 1.91–1.86 (m, 2H). ^13^C NMR δ: 192.0, 166.3, 162.4, 161.7, 159.7, 144.5, 137.7, 136.2, 133.0, 132.3, 132.2, 128.6, 128.5, 127.3, 126.6, 125.2, 123.4, 121.8, 121.5, 121.3, 118.8, 114.4, 111.2, 110.3, 105.6, 105.4, 97.9, 68.7, 46.1, 45.1, 32.2, 25.9, 25.4. LC-MS m/z for C_34_H_27_F_4_N_3_O_2_S_2_ [M+H]^+^ Calcd. 650.15. Found. 650.10.

(E/Z)-5-((1-(4-((6-Chloro-2-(trifluoromethyl)quinolin-4-yl)oxy)butyl)-1H-indol-3-yl)methylene)-3-phenethyl-2-thioxothiazolidin-4-one (**9f**)

Yellow solid, mp: 181–183 °C, yield: 73%. IR: ν_max_/cm^−1^ 3093, 2946, 1688, 1586, 1516, 1340, 1168, 1124, 1069. ^1^H NMR δ: 8.07–8.05 (m, 2H), 7.91 (s, 1H), 7.88 (s, 1H), 7.85 (d, *J* = 9.0, 1H), 7.83 (d, *J* = 9.0, 1H), 7.68 (d, *J* = 8.3, 1H), 7.32–7.29 (m, 4H), 7.28–7.20 (m, 4H), 4.48 (t, *J* = 6.8 Hz, 2H), 4.35 (t, *J* = 6.1 Hz, 2H), 4.22 (t, *J* = 7.9 Hz, 2H), 2.96 (t, *J* = 7.8 Hz, 2H), 2.13–2.07 (m, 2H), 1.91–1.86 (m, 2H). ^13^C NMR δ:192.1, 166.3, 162.0, 145.8, 136.2, 133.0, 131.9, 131.3, 128.7, 128.5, 127.3, 126.6, 125.2, 121.8, 120.6, 118.8, 111.1, 98.3, 68.6, 46.1, 45.2, 32.2, 25.9, 25.4. LC-MS m/z for C_34_H_27_ClF_3_N_3_O_2_S_2_ [M+H]^+^ Calcd. 666.12. Found. 666.10.

(E/Z)-5-((1-(4-((6-Methyl-2-(trifluoromethyl)quinolin-4-yl)oxy)butyl)-1H-indol-3-yl)methylene)-3-phenethyl-2-thioxothiazolidin-4-one (**9g**)

Yellow solid, mp: 175–177 °C, yield: 64%. IR: ν_max_/cm^−1^ 2942, 2876, 1697, 1594, 1574, 1382, 1254, 1169, 1129, 1095. ^1^H NMR δ: 7.99 (s, 1H), 7.94–7.88 (m, 4H), 7.69 (t, *J* = 8.9 Hz, 2H), 7.33–7.20 (m, 8H), 4.49 (t, *J =* 7.0 Hz, 2H), 4.33 (t, *J* = 6.3 Hz, 2H), 4.23 (t, *J* = 7.8 Hz, 2H), 2.96 (t, *J* = 7.8 Hz, 2H), 2.49 (s, 3H), 2.14–2.09 (m, 2H), 1.92–1.86 (m, 2H). ^13^C NMR δ: 192.1, 166.3, 162.1, 145.9, 137.9, 137.7, 136.3, 133.4, 133.1, 128.9, 128.7, 128.5, 127.4, 126.6, 125.3, 123.5, 121.9, 121.0, 120.3, 118.8, 114.4, 111.2, 110.4, 97.4, 68.3, 46.0, 45.2, 32.2, 26.0, 25.5, 21.4. LC-MS m/z for C_35_H_30_F_3_N_3_O_2_S_2_ [M+H]^+^ Calcd. 646.17. Found. 646.10.

(E/Z)-3-Phenethyl-2-thioxo-5-((1-(6-((2-(trifluoromethyl)quinolin-4-yl)oxy)hexyl)-1H-indol-3-yl)methylene)thiazolidin-4-one (**9h**)

Yellow solid, mp: 180–182 °C, yield: 84%. IR: ν_max_/cm^−1^ 3025, 2944, 1699, 1591, 1574, 1513, 1171, 1157, 1134. ^1^H NMR δ: 8.17–8.15 (d, *J* = 8.4 Hz, 1H), 8.06–8.05 (d, *J* = 8.6 Hz, 1H), 7.96–7.94 (d, *J =* 6.3 Hz, 2H), 7.92–7.90 (d, *J* = 7.8, 1H), 7.86 (t, *J* = 7.6 Hz, 1H), 7.77 (t, *J* = 7.6 Hz, 1H), 7.61 (d, *J* = 8.1 Hz, 1H), 7.31–7.21 (m, 8H), 4.36–4.29 (m, 4H), 4.20 (t, *J* = 7.8 Hz, 2H), 2.93 (t, *J* = 7.8 Hz, 2H), 1.87–1.81 (m, 4H), 1.57–1.50 (m, 2H), 1.41–1.34 (m, 2H). ^13^C NMR δ: 192.0, 166.3, 162.9, 147.3, 137.7, 136.2, 133.1, 131.3, 129.1, 128.7, 128.5, 127.9, 127.4, 126.6, 125.4, 123.4, 121.8, 121.7, 121.2, 118.8, 114.3, 111.1. 110.3, 97.4, 69.2, 46.4, 45.1, 32.2, 29.4, 28.1, 25.8, 25.1. LC-MS m/z for C_36_H_32_F_3_N_3_O_2_S_2_ [M+H]^+^ Calcd. 660.19. Found. 660.20.

(E/Z)-5-((1-(6-((6-Fluoro-2-(trifluoromethyl)quinolin-4-yl)oxy)hexyl)-1H-indol-3-yl)methylene)-3-phenethyl-2-thioxothiazolidin-4-one (**9i**)

Yellow solid, mp: 185–187 °C, yield: 70%. IR: ν_max_/cm^−1^ 2946, 2878, 1697, 1593, 1252, 1167, 1155, 1124. ^1^H NMR δ: 8.16–8.17 (m, 1H), 7.95 (s, 1H), 7.91 (d, *J* = 7.8, 2H), 7.79–7.77 (m, 2H), 7.62 (d, *J* = 8.2 Hz, 1H), 7.35 (s, 1H), 7.31–7.20 (m, 7H), 4.37 (t, *J* = 7.0 Hz, 2H), 4.31 (t, *J* = 6.4 Hz, 2H), 4.21 (t, *J* = 7.8 Hz, 2H), 2.95 (t, *J* = 7.8, 2H), 1.90–1.81 (m, 4H), 1.57–1.51 (m, 2H), 1.41–1.34 (m, 2H). ^13^C NMR δ: 192.0, 166.3, 162.5, 161.6, 144.5, 137.7, 136.2, 133.1, 132.4, 132.3, 128.7, 128.5, 127.4, 126.6, 125.4, 123.4, 121.8, 120.4, 118.8, 114.3, 111.1, 110.2, 97.9, 69.4, 46.4, 45.2, 32.2, 29.4, 28.0, 25.7, 25.0. LC-MS m/z for C_36_H_31_F_4_N_3_O_2_S_2_ [M+H]^+^ Calcd. 678.18. Found. 678.20.

(E/Z)-5-((1-(6-((6-Chloro-2-(trifluoromethyl)quinolin-4-yl)oxy)hexyl)-1H-indol-3-yl)methylene)-3-phenethyl-2-thioxothiazolidin-4-one (**9j**)

Yellow solid, mp: 184–186 °C, yield: 68%. IR: ν_max_/cm^−1^ 2943, 1697, 1594, 1365, 1253, 1189, 1124, 1095. ^1^H NMR δ: 8.08 (d, *J* = 9.2 Hz, 2H), 7.94–7.85 (m, 4H), 7.62 (d, *J* = 8.1 Hz, 1H), 7.36 (s, 1H), 7.30 (t, *J* = 7.6, 3H), 7.23 (t, *J* = 7.1 Hz, 4H), 4.36 (t, *J* = 7.0 Hz, 2H), 4.30 (t, *J* = 6.4, 2H), 4.20 (t, *J* = 7.8 Hz, 2H), 2.94 (t, *J* = 7.8 Hz, 2H), 1.86–1.83 (m, 4H), 1.56–1.49 (m, 2H), 1.40–1.33 (m, 2H). ^13^C-NMR δ: 190.2, 166.1, 162.8, 151.4, 144.3, 138.7, 136.1, 133.4, 132.1, 132.0, 128.9, 128.3, 127.1, 126.6, 125.5, 123.3, 121.6, 120.4, 119.0, 114.1, 111.1, 110.4, 98.1, 69.1, 48.4, 43.2, 31.1, 29.3, 27.9, 25.4. LC-MS m/z for C_36_H_31_ClF_3_N_3_O_2_S_2_ [M+H]^+^ Calcd. 694.15. Found. 694.20.

(E/Z)-5-((1-(6-((6-Methyl-2-(trifluoromethyl)quinolin-4-yl)oxy)hexyl)-1H-indol-3-yl)methylene)-3-phenethyl-2-thioxothiazolidin-4-one (**9k**)

Yellow solid, mp: 148–150 °C, yield: 80%. IR: ν_max_/cm^−1^ 2944, 1699, 1591, 1574, 1517, 1364, 1319, 1173, 1133, 1173, 1133, 1090. ^1^H NMR δ: 7.97–7.91 (m, 4H), 7.69 (t, *J* = 8.0 Hz, 1H), 7.62 (d, *J* = 8.3 Hz, 1H), 7.32–7.21 (m, 9H), 4.37–4.27 (m, 4H), 4.20 (t, *J* = 5.9 Hz, 2H), 2.94 (t, *J* = 7.8 Hz, 2H), 2.48 (s, 3H), 1.88–1.84 (m, 4H), 1.57–1.51 (m, 2H), 1.41–1.35 (m, 2H). ^13^C NMR δ: 192.0, 166.3, 162.3, 145.9, 137.9, 137.7, 135.8, 133.4, 133.1, 131.3, 129.1, 128.9, 128.7, 128.7, 128.5, 128.0, 127.4, 126.6, 125.5, 125.4, 123.4, 121.8, 121.7, 121.2, 121.1, 120.3, 118.8, 114.3, 111.1, 110.3, 97. 3, 69.2, 46.4, 45.1, 29.4, 25.8, 25.7, 25.1, 25.0. LC-MS m/z for C_37_H_34_F_3_N_3_O_2_S_2_ [M+H]^+^ Calcd. 674.20. Found. 674.20.

(E/Z)-3-Phenethyl-2-thioxo-5-((1-((E)-4-((2-(trifluoromethyl)quinolin-4-yl)oxy)but-2-en-1-yl)-1H-indol-3-yl)methylene)thiazolidin-4-one (**10a**)

Yellow solid, mp: 179–181 °C, yield: 80%. IR: ν_max_/cm^−1^ 3026, 2949, 1698, 1595, 1371, 1261, 1170, 1111. ^1^H NMR δ: 8.19 (d, *J* = 8.4 Hz, 1H), 8.06 (d, *J* = 8.4, 1H), 8.01 (d, *J* = 6.6 Hz, 2H), 7.97 (d, *J* = 9.0 Hz, 1H), 7.87 (t, *J* = 7.8 Hz, 1H), 7.70 (t, *J* = 7.7 Hz, 1H), 7.60 (d, *J* = 8.0 Hz, 1H), 7.34 (s, 1H), 7.31–7.21 (m, 7H), 6.26–6.21 (m, 1H), 6.06–6.00 (m, 1H), 5.10 (d, *J* = 6.0 Hz, 2H), 4.98 (d, *J* = 5.8 Hz, 2H), 4.35 (t, *J* = 7.8, 2H), 2.95 (t, *J* = 7.7 Hz, 2H). ^13^C NMR δ: 192.0, 166.4, 162.3, 147.4, 137.7, 136.2, 133.0, 131.4, 129.3, 129.1 128.7, 128.5, 128.1, 127.5, 127.3, 126.6, 125.3, 123.5, 122.0, 121.7, 121.1, 118.9, 114.8, 111.4, 110.6, 97.9, 68.5, 47.8, 45.2, 32.2. LC-MS m/z for C_34_H_26_F_3_N_3_O_2_S_2_ [M+H]^+^ Calcd. 630.14. Found. 630.10.

(E/Z)-5-((1-((E)-4-((6-Methyl-2-(trifluoromethyl)quinolin-4-yl)oxy)but-2-en-1-yl)-1H-indol-3-yl)methylene)-3-phenethyl-2-thioxothiazolidin-4-one (**10b**)

Yellow solid, mp: 168–170 °C, yield: 49%. IR: ν_max_/cm^−1^ 2948, 2864, 1698, 1594, 1345, 1279, 1169, 1131, 1096. ^1^H NMR δ: 8.00 –7.93 (m, 5H), 7.68 (d, *J* = 8.7 Hz, 1H), 7.60 (d, *J* = 7.8 Hz, 1H), 7.31–7.21 (m, 8H), 6.25–6.20 (m, 1H), 6.05–5.99 (m, 1H), 5.10 (d, *J* = 6.0 Hz, 2H), 4.95 (d, *J* = 5.7 Hz, 2H), 4.22 (t, *J* = 7.9 Hz, 2H), 2.96 (t, *J* = 7.7 Hz, 2H), 2.50 (s, 3H). ^13^C NMR δ:192.0, 166.4, 160.5, 146.0, 138.0, 137.7, 136.2, 133.4, 133.0, 129.4, 128.9, 128.7, 128.5, 127.5, 127.4, 126.6, 125.3, 123.5, 121.1, 120.3, 118.9, 114.7, 11.4, 110.6, 97.9, 68.4, 47.8, 45.2, 32.2, 21.4. LC-MS m/z for C_35_H_28_F_3_N_3_O_2_S_2_ [M+H]^+^ Calcd. 644.16. Found. 644.2.

### 4.4. HPLC-MS Conditions

The high-pressure liquid chromatographic (HPLC) studies were carried out on a system consisting of an Agilent 1100 Series with a 1260 Infinity II LC separations module, an automatic injector for volumes ranging from 0.1 to 100 µL, and a 1260 infinity II diode array detector HS (Agilent Technologies Inc., Santa Clara, CA, USA). A stationary phase (Agilent Prep-C18 Scalar column with internal diameter 4.6 mm and length 100 mm, and particle size 5-micron, Agilent Technologies Inc., Santa Clara, CA, USA) and mobile phase solvents (water and acetonitrile) were used. Water contained 0.1% of formic acid.

### 4.5. Biological Data

#### 4.5.1. MTT Cytotoxicity Assay

To assess the half-maximal cytotoxic concentration (CC_50_), stock solutions of the tested compounds were prepared in 10% DMSO in ddH_2_O and diluted further to the working solutions with DMEM. The cytotoxic activity of the compounds was tested in normal *Chlorocebus aethiops* kidney VERO-E6 cells by using the 3-(4,5-dimethylthiazol-2-yl)-2,5-diphenyltetrazolium bromide (MTT) method with minor modifications. Briefly, the cells were seeded in 96-well plates (9100 µL/well at a density of 3 × 10^5^ cell/mL) and incubated for 24 h at 37 °C in 5% CO_2_. After 24 h, cells were treated with various concentrations of the tested compounds in triplicates. 24 h later, the supernatant was discarded, and cell monolayers were washed with sterile 1x phosphate buffer saline (PBS) three times. MTT solution (20 µL of 5 mg/mL) was added to each well and the cells incubated at 37 °C for 4 h and then underwent medium aspiration. In each well, the formed formazan crystals were dissolved with 200 µL of acidified isopropanol (0.04 M HCl in absolute isopropanol = 0.073 mL HCl in 50 mL isopropanol). The absorbance of formazan solutions was measured at *λ*_max_ 540 nm with 620 nm as a reference wavelength using a multi-well plate reader [20,21,22,23].

#### 4.5.2. IC_50_ Determination

In 96-well tissue culture plates, 2.4 × 10^4^ Vero-E6 cells were distributed in each well and incubated overnight in a humidified 37 °C incubator under 5% CO_2_ condition. The cell monolayers were then washed with 1x PBS and subjected to virus absorption (hCoV-19/Egypt/NRC-03/2020, Accession Number on GSAID: EPI_ISL_430820) for 1 h at room temperature (RT). The cell monolayers were further overlaid with 50 µL of DMEM containing varying concentrations of the test sample and then incubated at 37 °C in a 5% CO_2_ incubator for 72 h. The cells were fixed with 100 µL of 4% paraformaldehyde for 20 min. and stained with 0.1% crystal violet in distilled water for 15 min. at RT. The crystal violet dye was then dissolved using 100 µL absolute methanol per well and the optical density of the color was measured at 570 nm using an Anthos Zenyth 200 rt plate reader (Anthos Labtec Instruments, Heerhugowaard, Netherlands). The IC_50_ of the compound was that required to reduce the virus-induced cytopathic effect (CPE) by 50%, relative to the virus control [20,21,22,23].

## Figures and Tables

**Table 1:** Anti-SARS-CoV-2 results for the promising agents discovered.

Entry	Compd.	IC_50_, µg/mL (µM)	CC_50_, µg/mL (µM)	SI
1	**9d**	257.2 (407.1)	293.9 (465.2)	1.1
2	**9e**	275.6 (424.2)	304.8 (469.1)	1.1
3	**9f**	109.0 (163.6)	369.6 (554.8)	3.4
4	**9k**	160.1 (237.6)	201.3 (298.7)	1.3
5	**Hydroxychloroquine** [22]	(29.25)	(356.4)	12.2
6	**Remdesivir** [27] ^a^	(3.38)	(58.12)	17.18

^a^ Against RAW264.7 cells.

**Table 2:** CDOCKER interaction energy scores, hydrogen bonding interactions and π interactions in PDB ID: 6LU7.

Entry	Compd.	Hydrogen Bonding Interactions	CDOCKER Interaction Energy Scores (Kcal mol^−1^)
1	**9d**	Hydrogen bonding: quinolinyl O–GLY143, π–σ interactions: quinoline–ASN142	−50.9
2	**9e**	Hydrogen bonding: quinolinyl O–GLY143, π–σ interactions: phenyl–PRO168	−49.4
3	**9f**	Hydrogen bonding: F of CF_3_–GLY143	−51.8
4	**9k**	Hydrogen bonding: F of CF_3_–GLY143	−50.7

**Table 3:** ADME descriptor values for the synthesized compounds.

Entry	Compd.	Aqueous Solubility	Intestinal Absorption	PPB
1	**9d**	3	0	2
2	**9e**	3	0	2
3	**9f**	3	0	2
4	**9k**	3	0	2

## Data Availability

Data are contained within the article or Supplementary Material.

## Supplementary Materials

The following supporting information can be downloaded at: https://www.mdpi.com/article/10.3390/molecules27185923/s1, 1H-,13C-NMR and HPLC-MS spectra of all synthetized molecules.

## Sample Availability

Samples of the compounds are available on request from the corresponding author.
