# Supplementary material for: Synthesis of Potential Antiviral Agents for SARS-CoV-2 Using Molecular Hybridization Approach
Source: Molecules. 2022 Sep 12;27(18):5923. doi: 10.3390/molecules27185923 (PMC9501548; doi:10.3390/molecules27185923)
Supplement: Supplementary file 1 [file molecules-27-05923-s001.zip › molecules-1898024-supplementary.pdf]

Supporting Information

Synthesis of Potential Antiviral Agents for SARS-CoV-2 using Molecular Hybridization Approach

Kailey A. Wyman,[a] Adel S. Girgis,[b] Pragnakiran Surapaneni,[a,c] Jade M. Moore,[a] Noura M. Abo Shama,[d] Sara H. Mahmoud,[d] Ahmed Mostafa,[d] Reham F. Barghash,[b] Zou Juan,[a] Radha D. Dobaria,[a] Ahmad J. Almalki,[e] Tarek S. Ibrahim,[e] Siva S. Panda,[a,]*

[a]*Department of Chemistry and Physics, Augusta University, Augusta, GA, 30912, USA*

[b]*Department of Pesticide Chemistry, National Research Centre, Dokki, Giza, 12622, Egypt*

[c]*Schulich School of Medicine and Dentistry, Western University, London, ON, N6A5C1, Canada*

[d]*Center of Scientific Excellence for Influenza Viruses, National Research Centre, Giza 12622, Egypt*

[e]*Department of Pharmaceutical Chemistry, Faculty of Pharmacy, King Abdulaziz University, Jeddah 21589, Saudi Arabia*

*Corresponding author, *E-mail address:* sipanda@augusta.edu, sspanda12@gmail.com

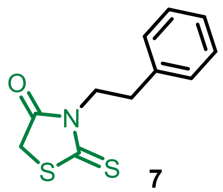

7

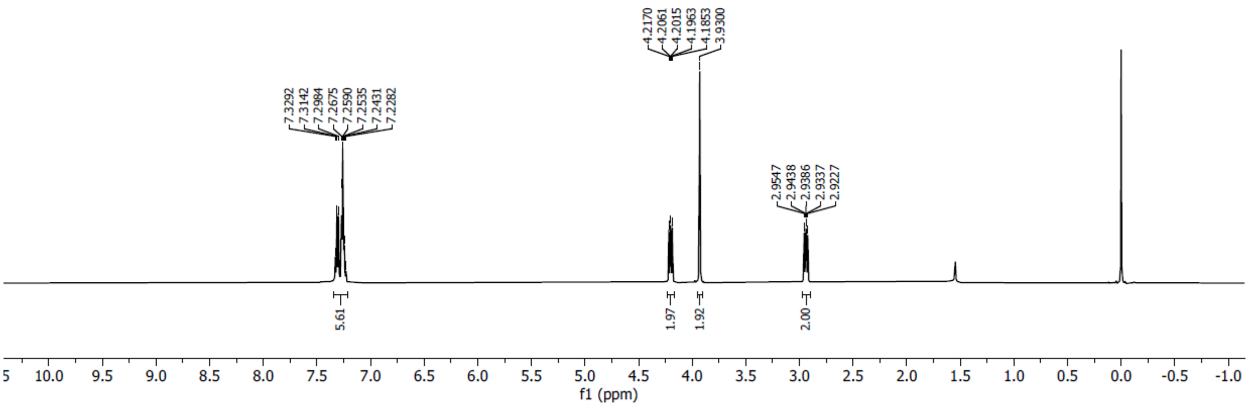
7.3292
7.3142
7.2984
7.2675
7.2590
7.2535
7.2431
7.2282
4.2170
4.2061
4.2015
4.1963
4.1853
3.9300
2.9547
2.9438
2.9386
2.9337
2.9227
5.61
1.97
1.92
2.00
f1 (ppm)

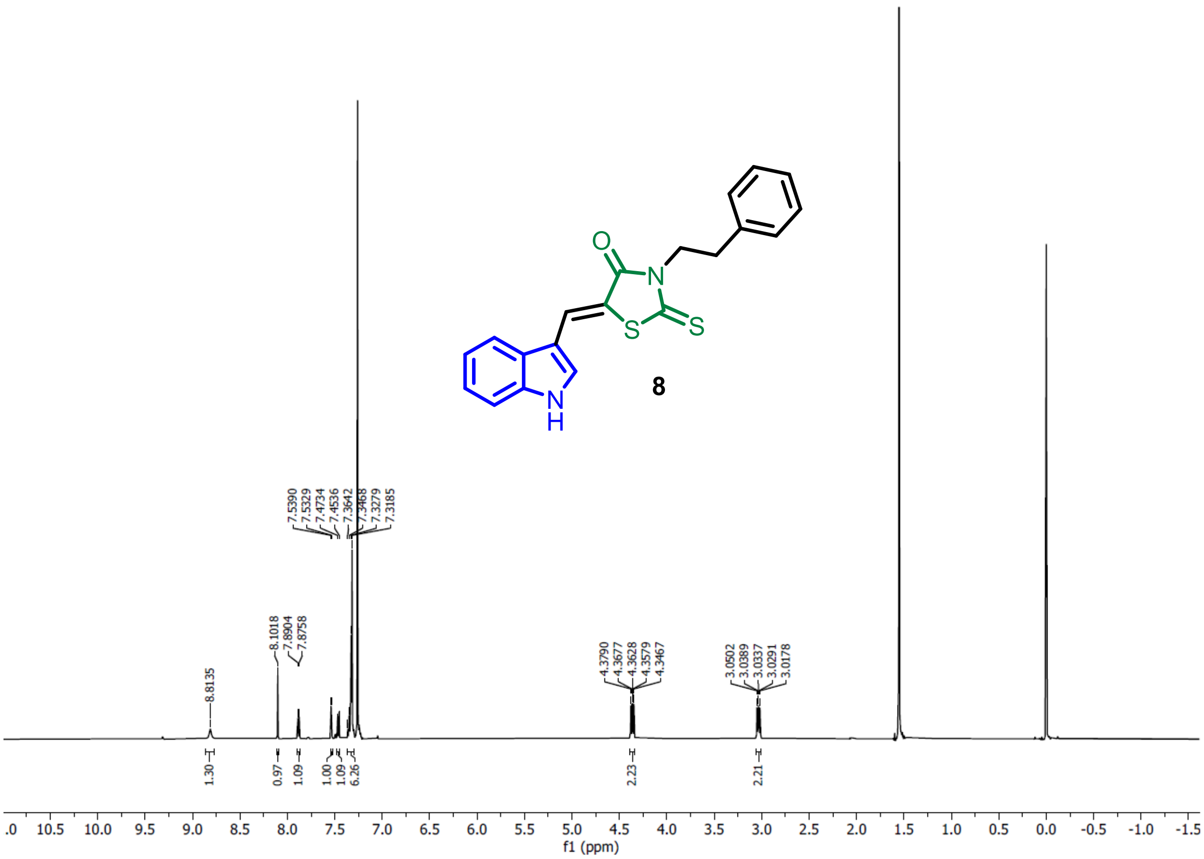
8
7.5390
7.5329
7.4734
7.4536
7.3642
7.3468
7.3279
7.3185
8.1018
7.8904
7.8758
8.8135
4.3790
4.3677
4.3628
4.3579
4.3467
3.0502
3.0389
3.0337
3.0291
3.0178
1.30
0.97
1.09
1.00
1.09
6.26
2.23
2.21
f1 (ppm)

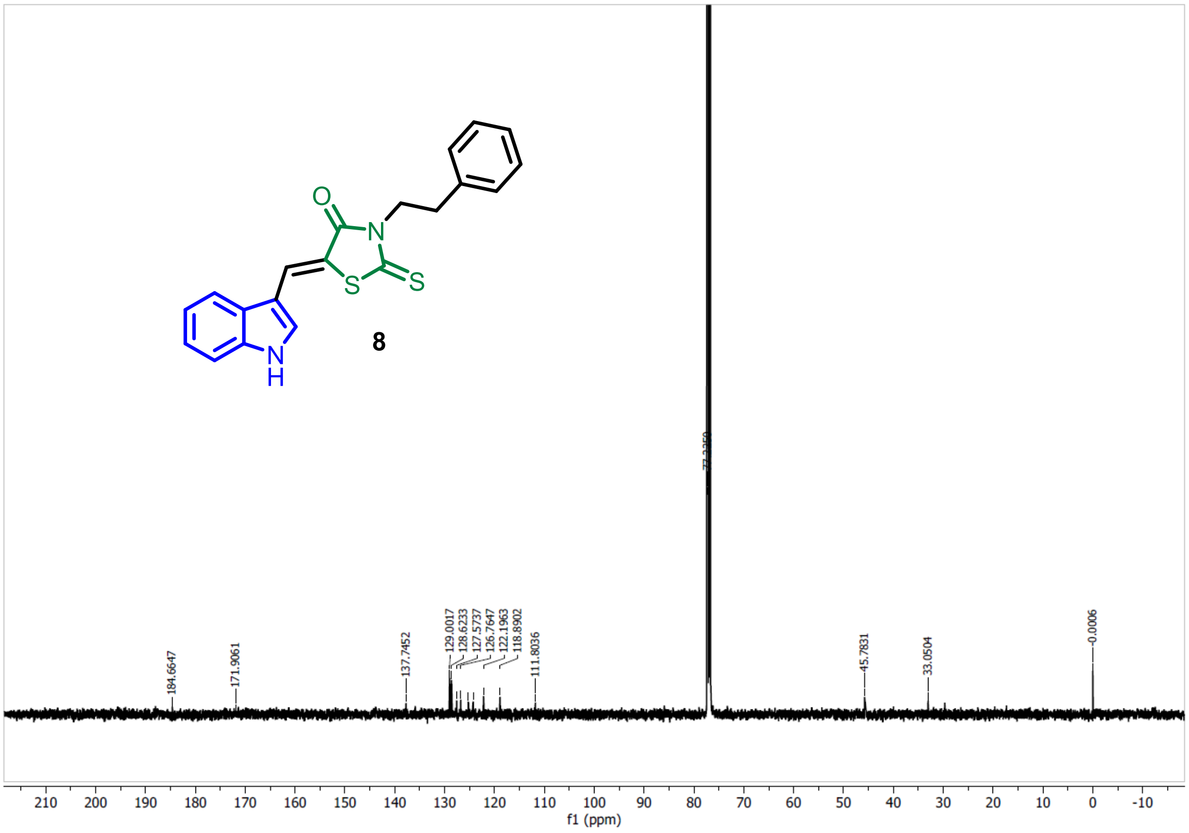
8
184.6647
171.9061
137.7452
129.0017
128.6233
127.5737
126.7647
122.1963
118.8902
111.8036
45.7831
33.0504
-0.0006
f1 (ppm)

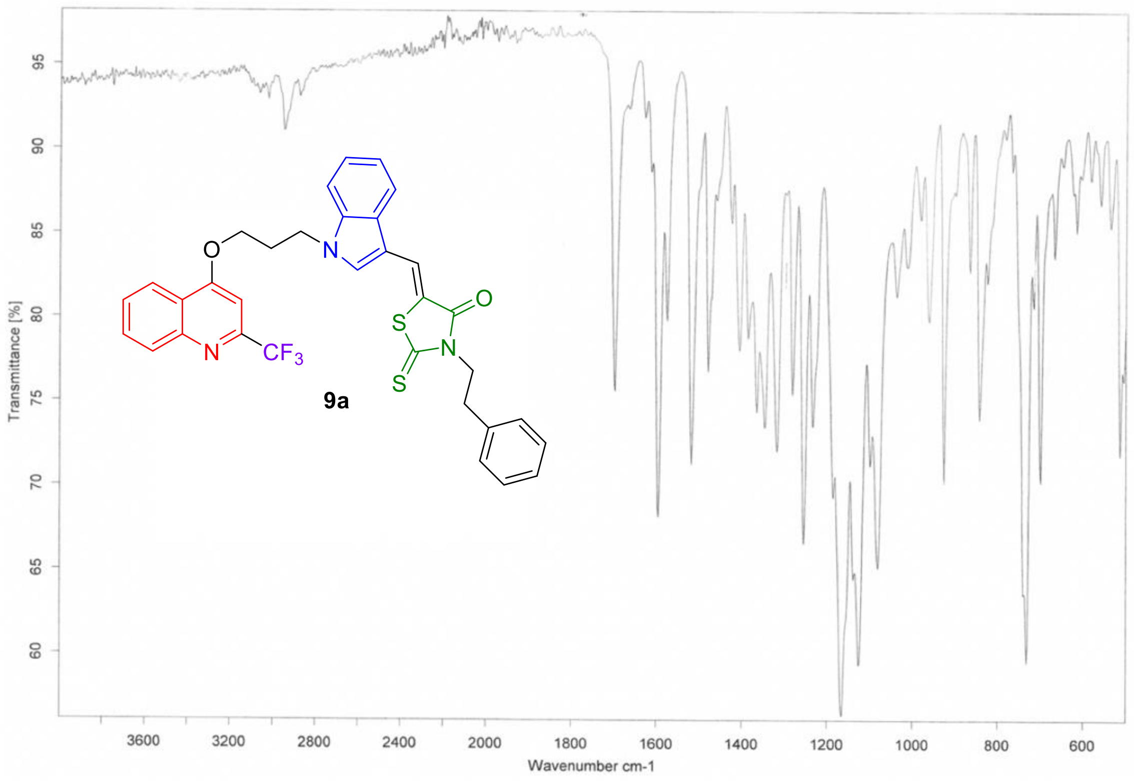

Transmittance [%]
Wavenumber cm-1
95
90
85
80
75
70
65
60
3600
3200
2800
2400
2000
1800
1600
1400
1200
1000
800
600
O
N
N
CF3
S
O
N
S
9a

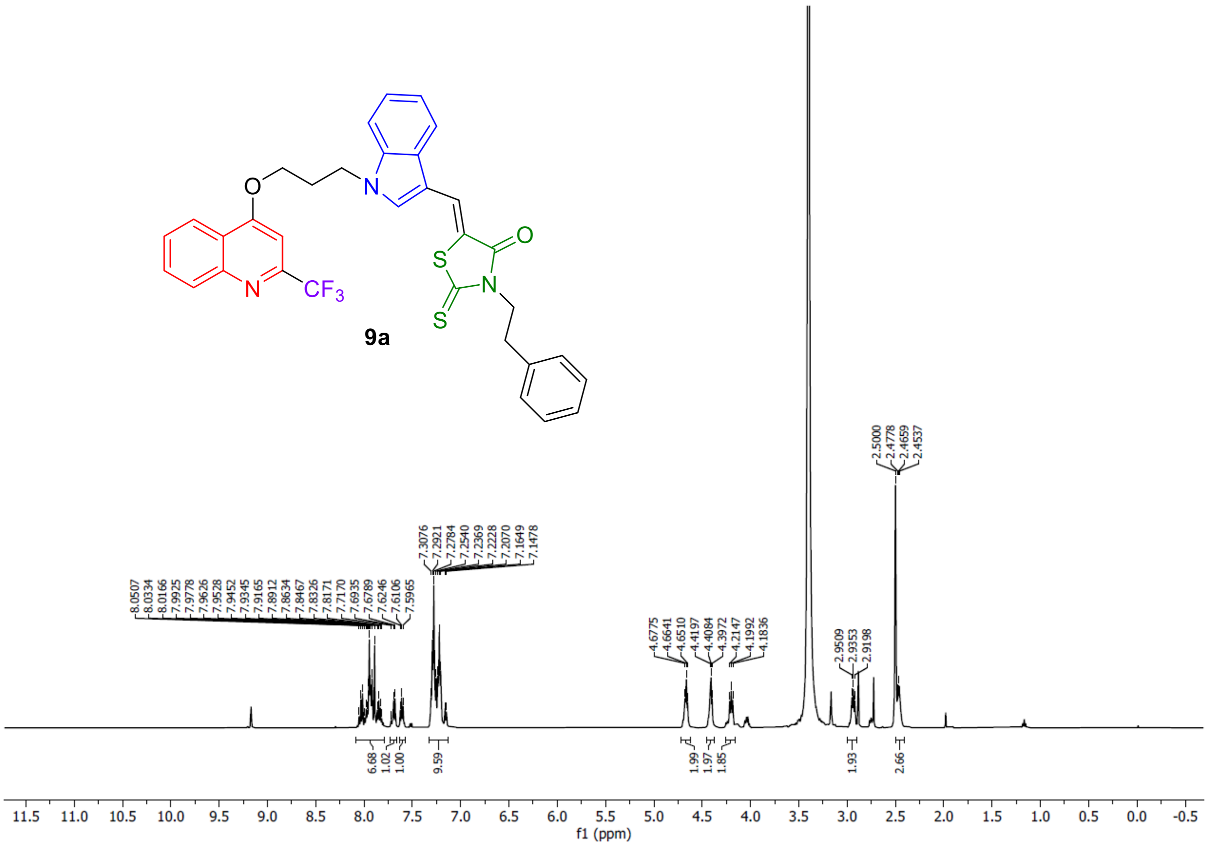

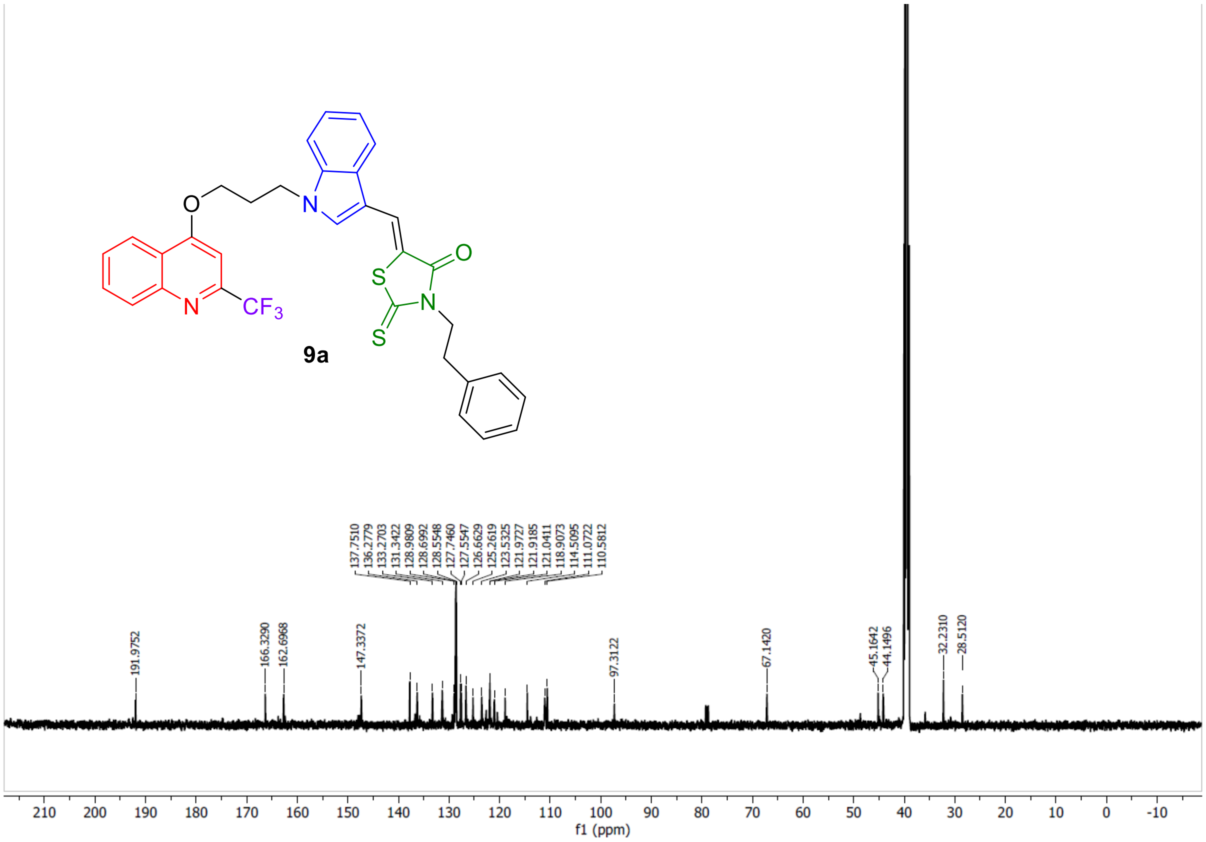

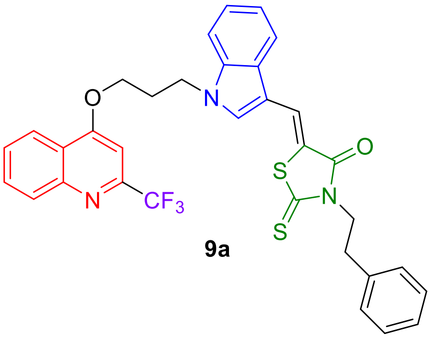

9a

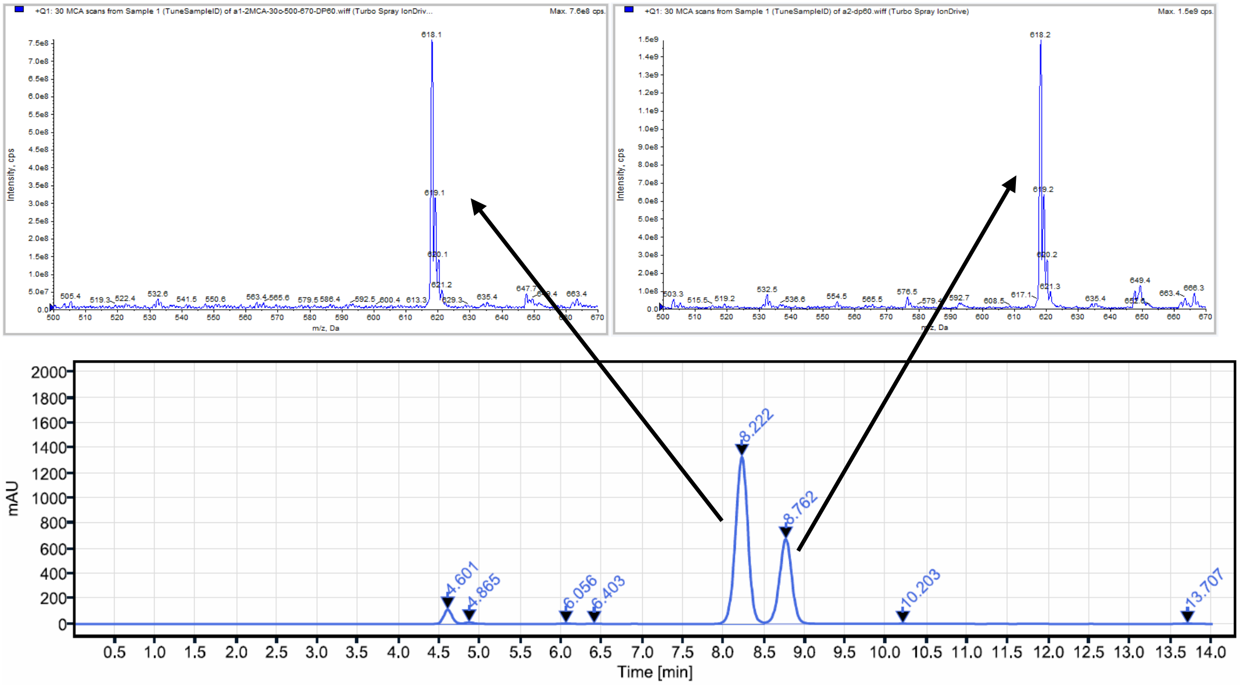

+Q1: 30 MCA scans from Sample 1 (TuneSampleID) of a1-2MCA-30o-500-670-DP60.wiff (Turbo Spray IonDriv...
Max. 7.6e8 cps.
+Q1: 30 MCA scans from Sample 1 (TuneSampleID) of a2-dp60.wiff (Turbo Spray IonDrive)
Max. 1.5e9 cps.
Intensity, cps
m/z, Da
618.1
618.2
mAU
Time [min]
4.601
4.865
6.056
6.403
8.222
8.762
10.203
13.707

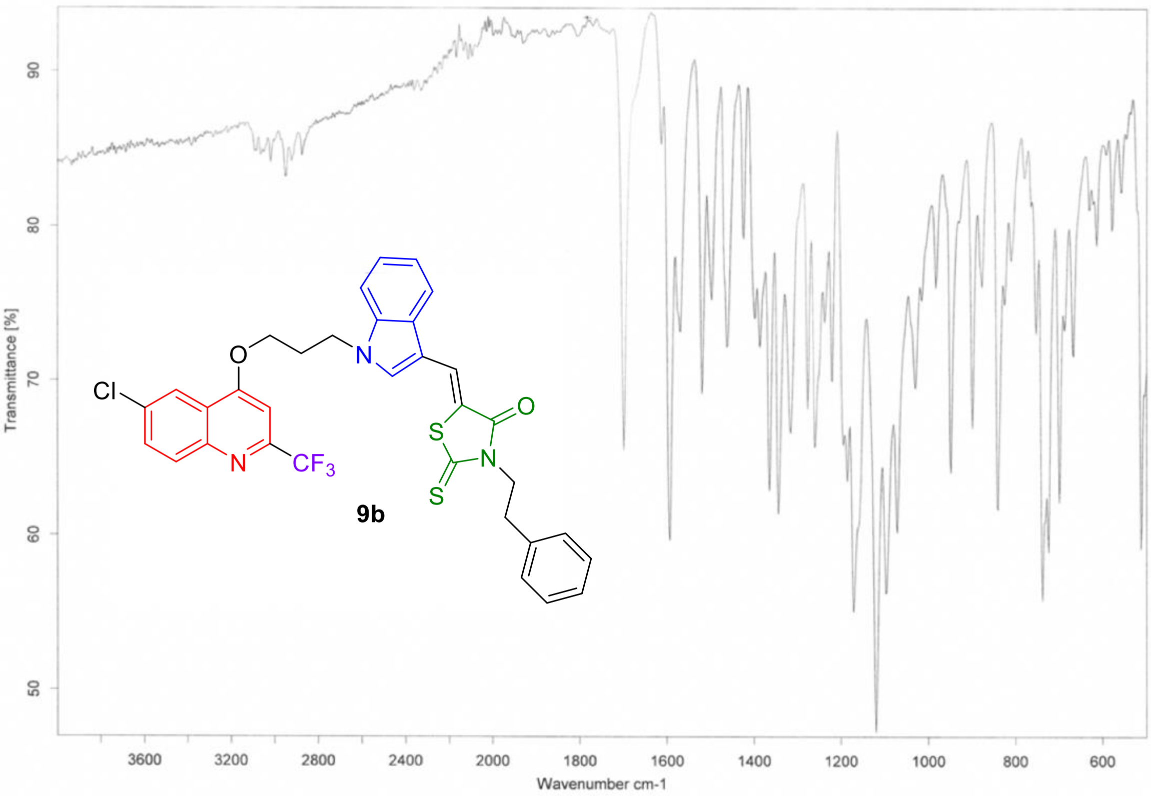
Transmittance [%]
90
80
70
60
50
3600
3200
2800
2400
2000
1800
1600
1400
1200
1000
800
600
Wavenumber cm-1
Cl
O
N
CF3
N
S
O
N
S
9b

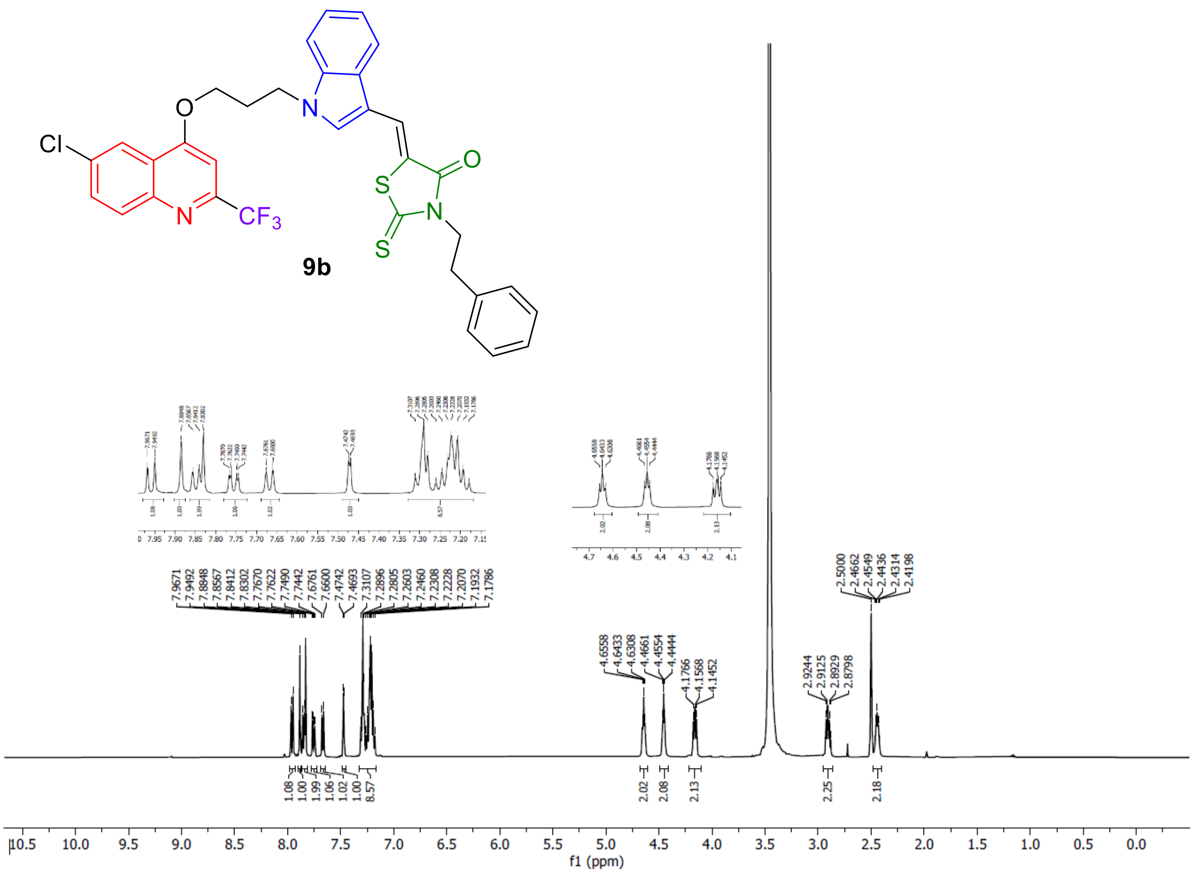

Cl
O
N
N
CF3
S
O
N
S
9b
7.9671 7.9492 7.8848 7.8567 7.8412 7.8302 7.7670 7.7622 7.7490 7.7442 7.6761 7.6600 7.4742 7.4693 7.3107 7.2896 7.2805 7.2603 7.2460 7.2308 7.2228 7.2070 7.1932 7.1786
4.6558 4.6433 4.6308 4.4661 4.4554 4.4444 4.1766 4.1568 4.1452
2.9244 2.9125 2.8929 2.8798
2.5000 2.4662 2.4549 2.4436 2.4314 2.4198
1.08 1.00 1.99 1.06 1.02 1.00 8.57 2.02 2.08 2.13 2.25 2.18
f1 (ppm)

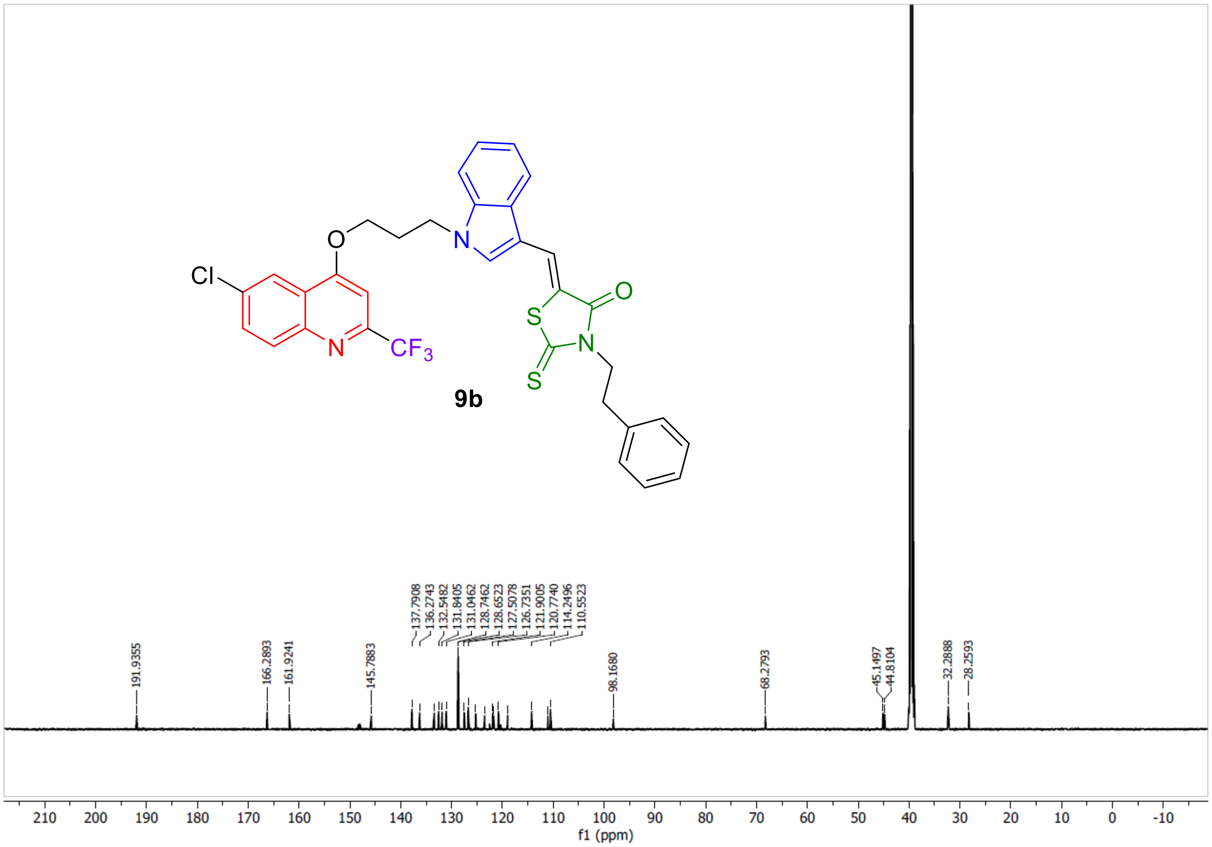

Cl
O
N
N
CF3
S
O
N
S
9b
191.9355
166.2893 161.9241
145.7883
137.7908 136.2743 132.5482 131.8405 131.0462 128.7462 128.6523 127.5078 126.7351 121.9005 120.7740 114.2496 110.5523
98.1680
68.2793
45.1497 44.8104
32.2888 28.2593
f1 (ppm)

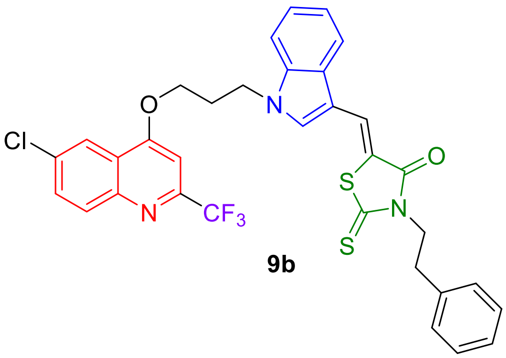

Cl
O
N
N
CF3
S
O
N
S

9b

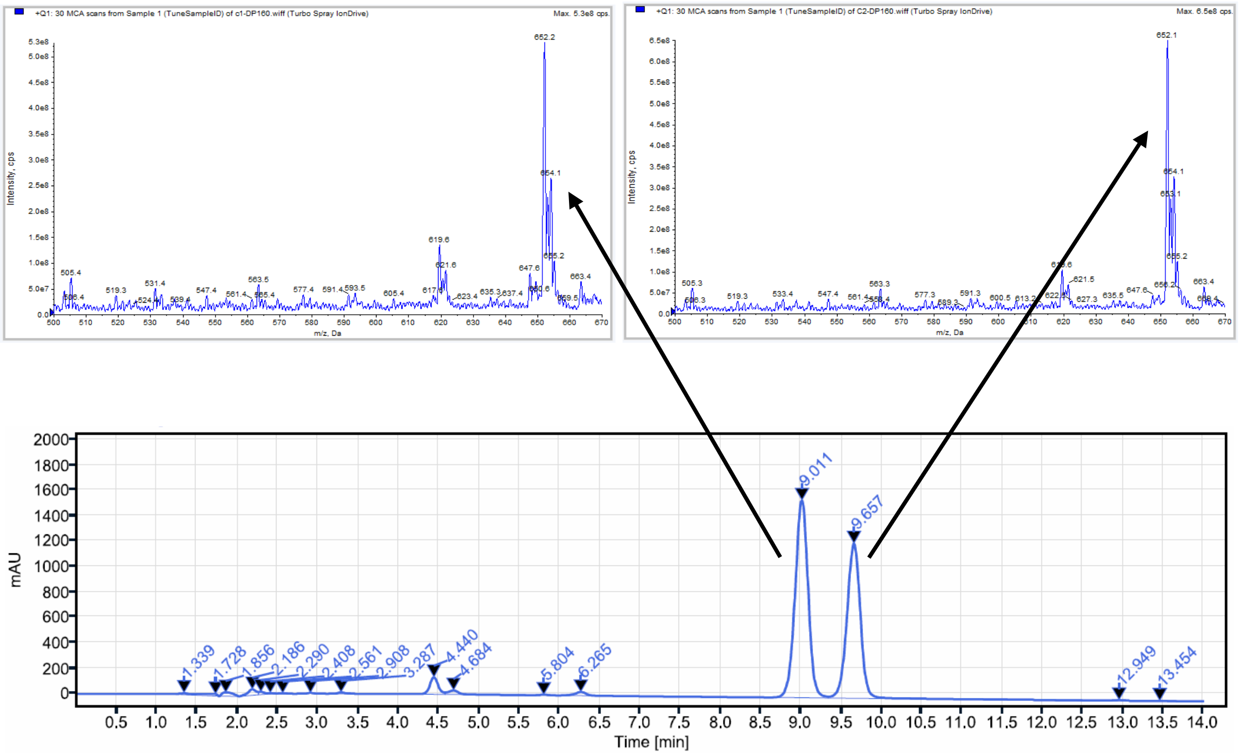

+Q1: 30 MCA scans from Sample 1 (TuneSampleID) of c1-DP160.wiff (Turbo Spray IonDrive)
Max. 5.3e8 cps.
652.2
654.1
619.6
Intensity, cps
m/z, Da
+Q1: 30 MCA scans from Sample 1 (TuneSampleID) of C2-DP160.wiff (Turbo Spray IonDrive)
Max. 6.5e8 cps.
652.1
654.1
mAU
Time [min]
9.011
9.657

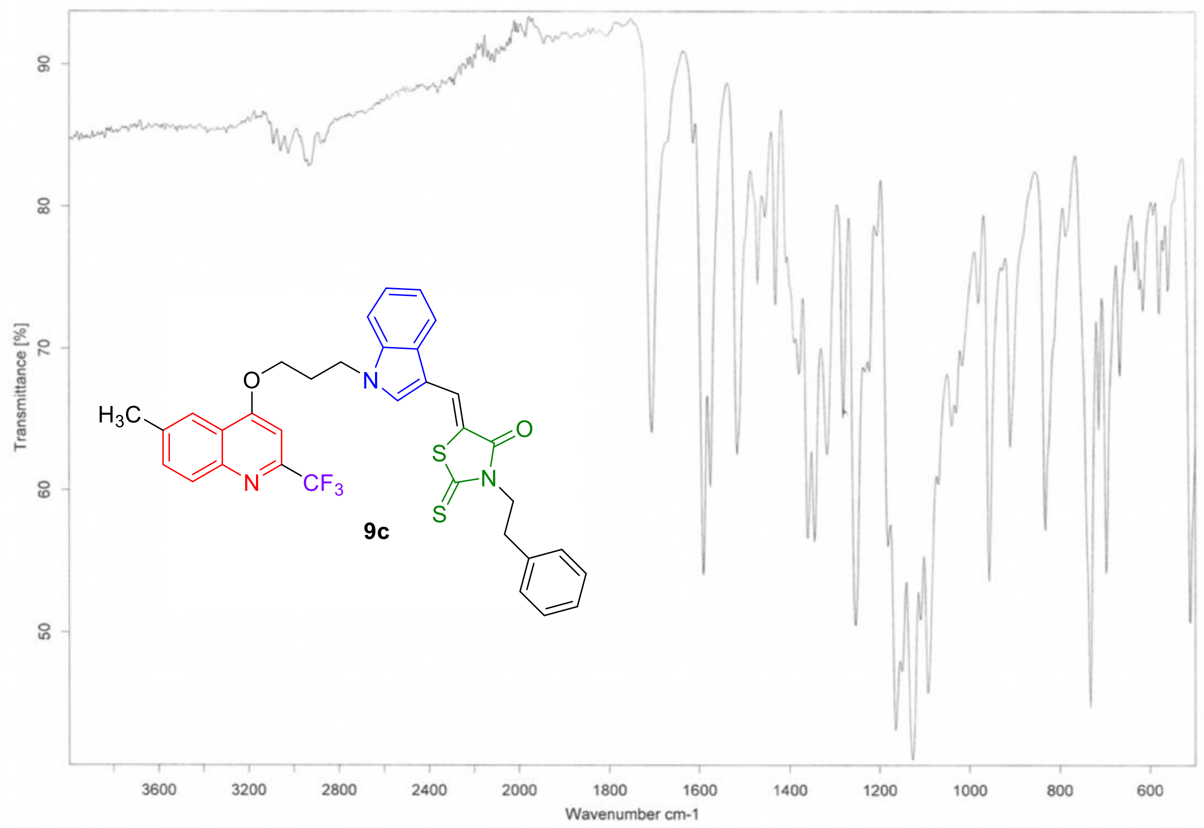

Transmittance [%]
Wavenumber cm-1
H3C
O
N
N
CF3
S
S
N
O
9c

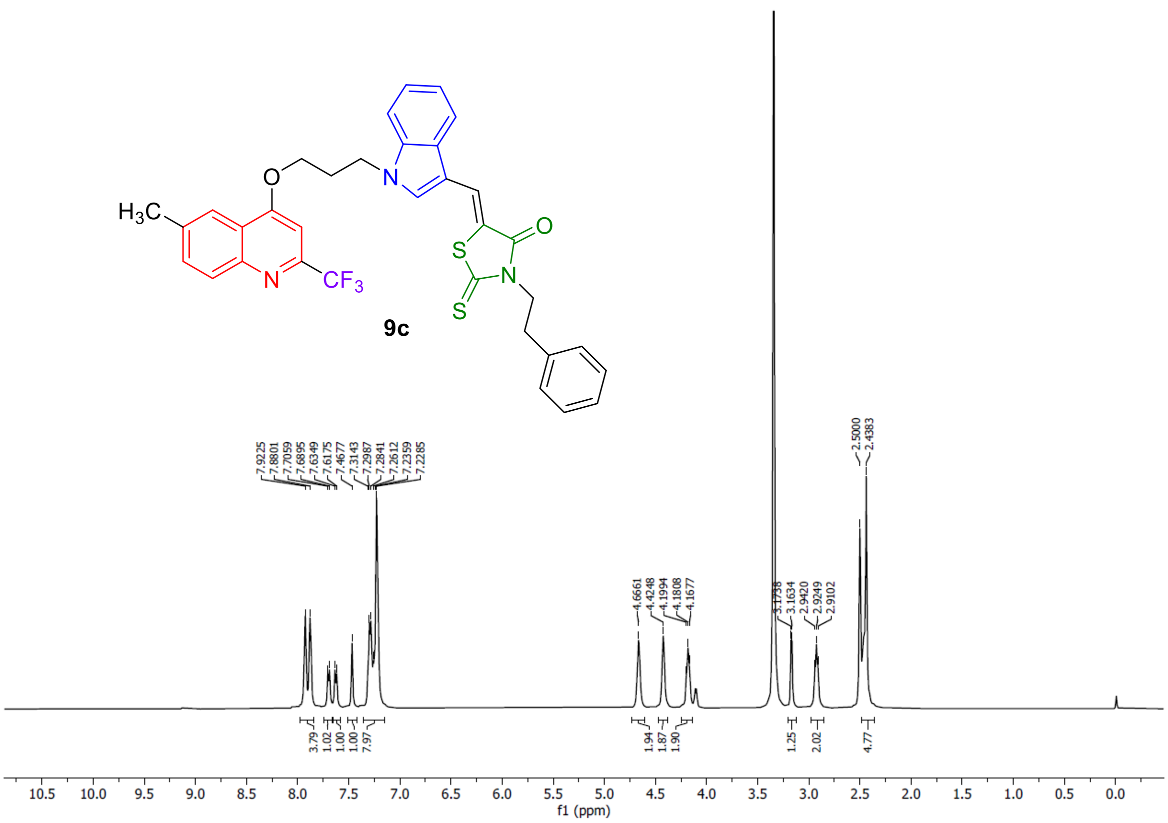

H3C
O
N
N
CF3
S
S
N
O
9c
7.9225
7.8801
7.7059
7.6895
7.6349
7.6175
7.4677
7.3143
7.2987
7.2841
7.2612
7.2359
7.2285
4.6661
4.4248
4.1994
4.1808
4.1677
3.1738
3.1634
2.9420
2.9249
2.9102
2.5000
2.4383
3.79
1.02
1.00
1.00
7.97
1.94
1.87
1.90
1.25
2.02
4.77
f1 (ppm)

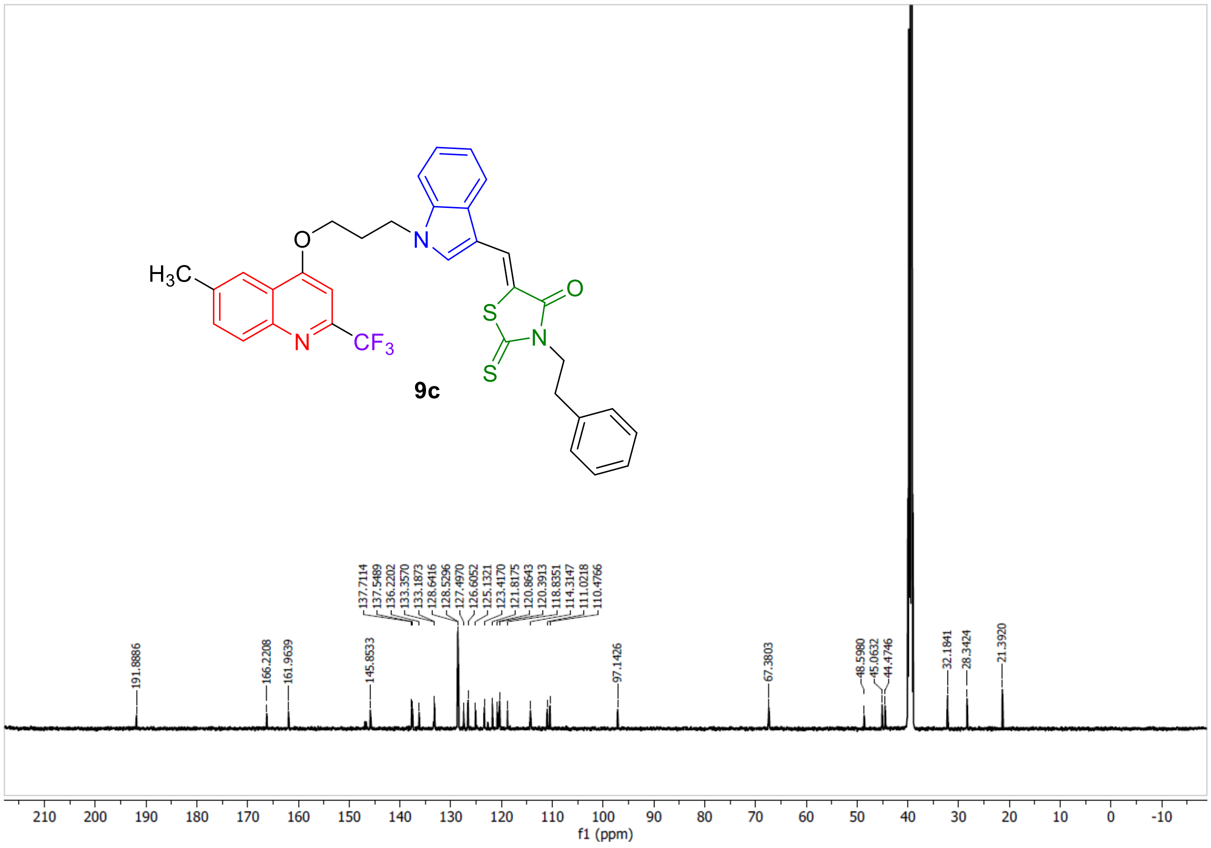

H3C
O
N
N
CF3
S
S
N
O
9c
191.8886
166.2208
161.9639
145.8533
137.7114
137.5489
136.2202
133.3570
133.1873
128.6416
128.5296
127.4970
126.6052
125.1321
123.4170
121.8175
120.8643
120.3913
118.8351
114.3147
111.0218
110.4766
97.1426
67.3803
48.5980
45.0632
44.4746
32.1841
28.3424
21.3920
f1 (ppm)

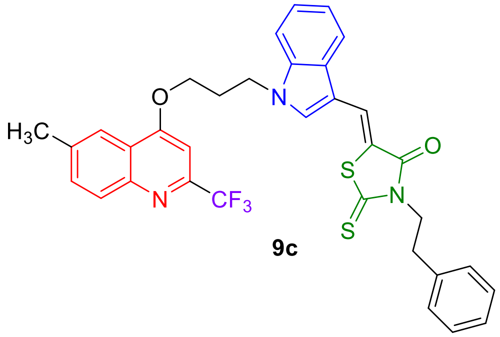
O
N
H3C
N
CF3
S
S
N
O

9c

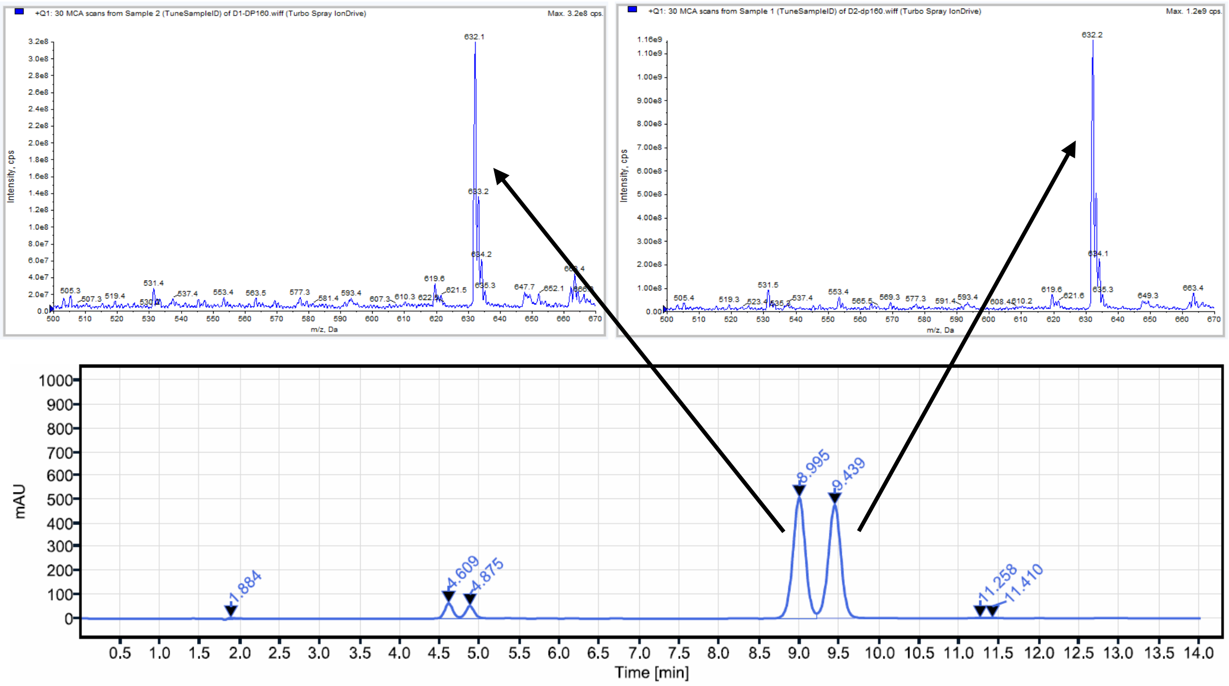
+Q1: 30 MCA scans from Sample 2 (TuneSampleID) of D1-DP160.wiff (Turbo Spray IonDrive)
Max. 3.2e8 cps.
Intensity, cps
m/z, Da
632.1
633.2
634.2
+Q1: 30 MCA scans from Sample 1 (TuneSampleID) of D2-dp160.wiff (Turbo Spray IonDrive)
Max. 1.2e9 cps.
Intensity, cps
m/z, Da
632.2
634.1
mAU
Time [min]
1.884
4.609
4.875
8.995
9.439
11.258
11.410

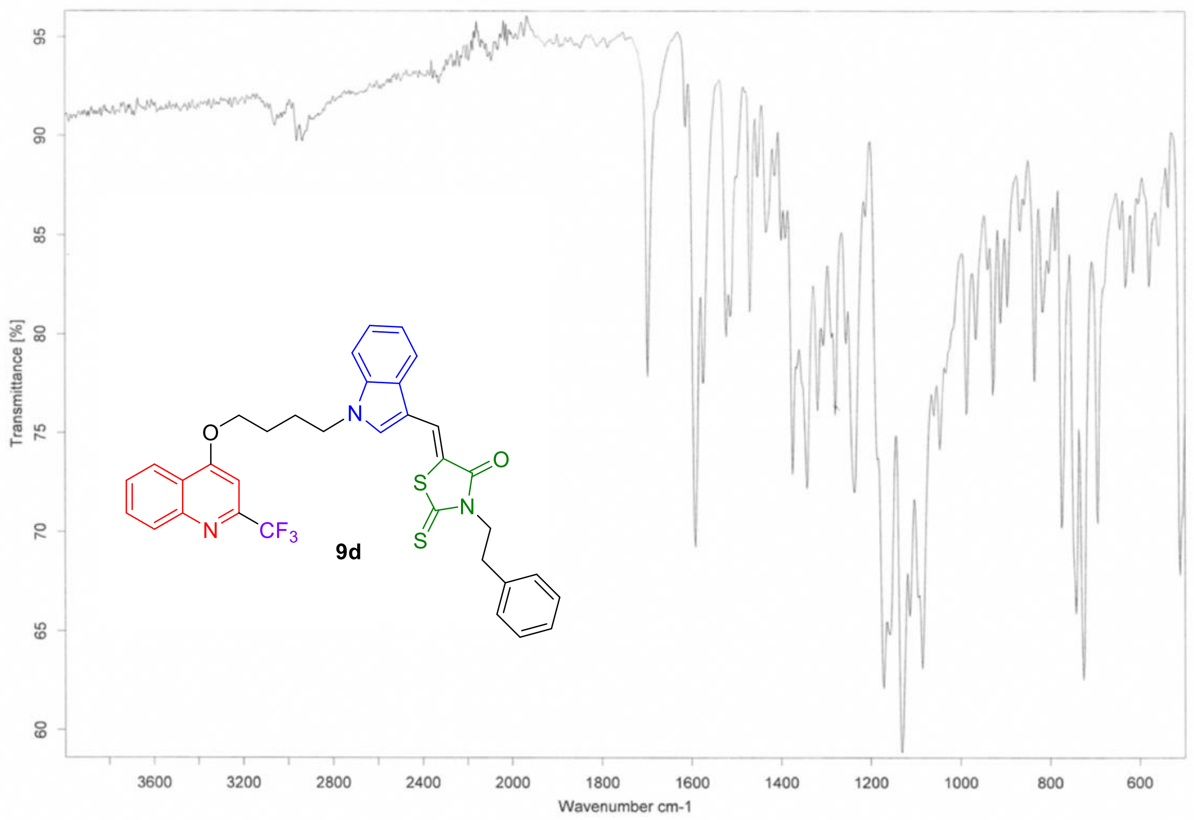

95
90
85
80
75
70
65
60
Transmittance [%]
3600
3200
2800
2400
2000
1800
1600
1400
1200
1000
800
600
Wavenumber cm-1
O
N
N
CF3
S
N
O
S
9d

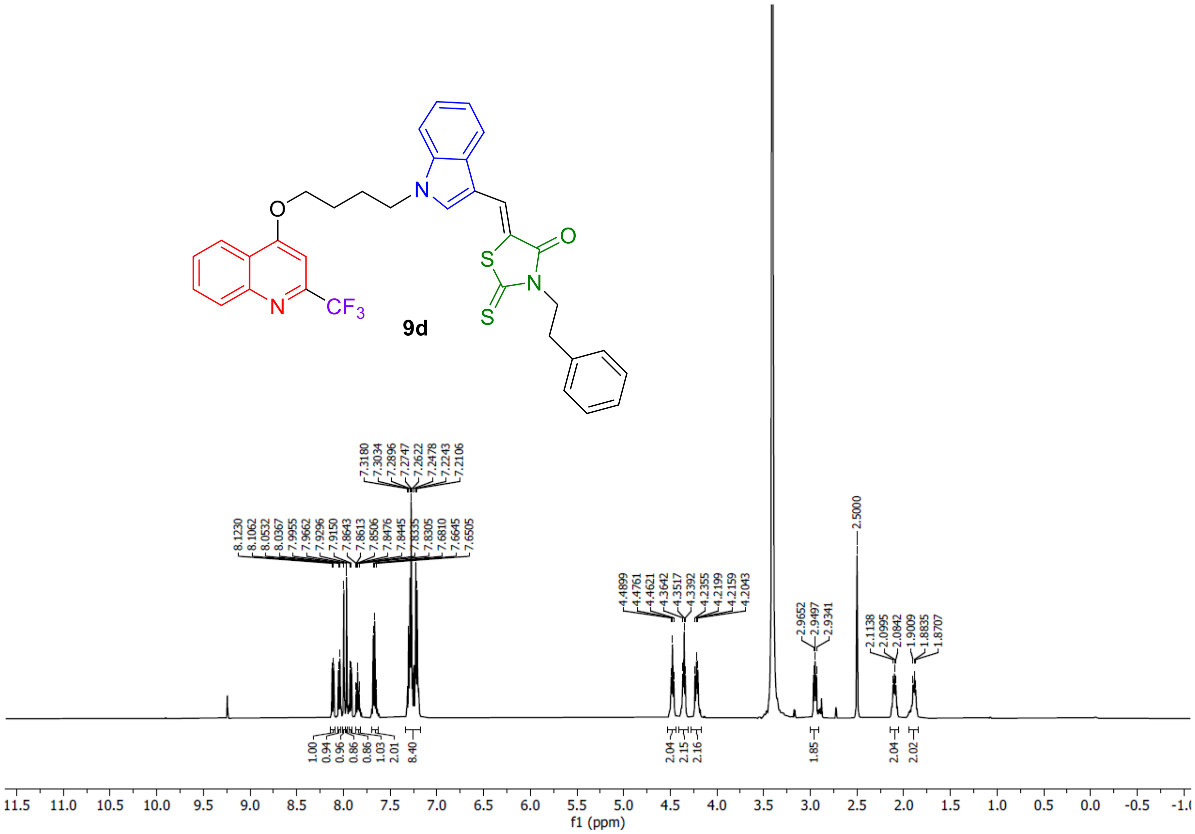
9d
f1 (ppm)

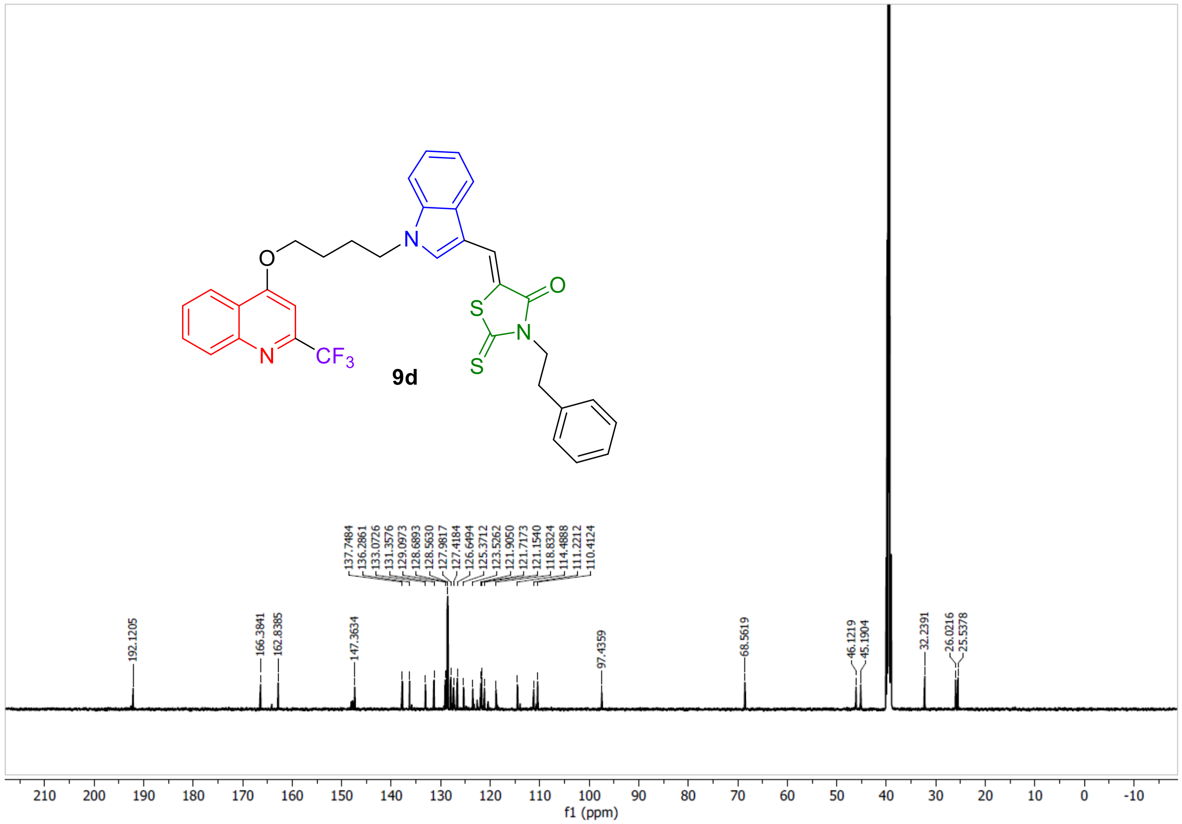
9d
f1 (ppm)

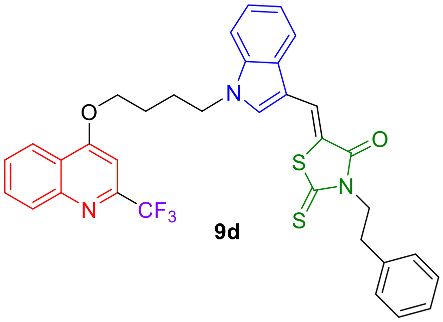
O
N
CF3
N
S
N
O
S

9d

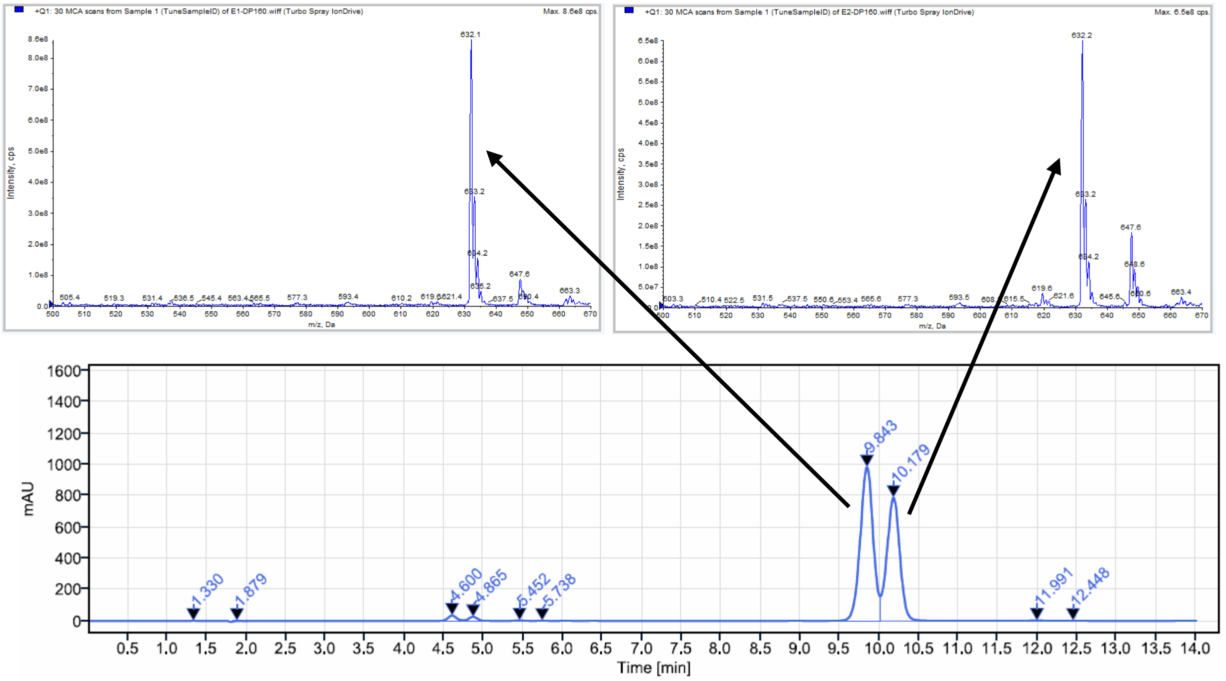
+Q1: 30 MCA scans from Sample 1 (TuneSampleID) of E1-DP160.wiff (Turbo Spray IonDrive)
Max. 8.6e8 cps.
632.1
647.6
+Q1: 30 MCA scans from Sample 1 (TuneSampleID) of E2-DP160.wiff (Turbo Spray IonDrive)
Max. 6.5e8 cps.
632.2
647.6
Intensity, cps
m/z, Da
9.843
10.179
mAU
Time [min]

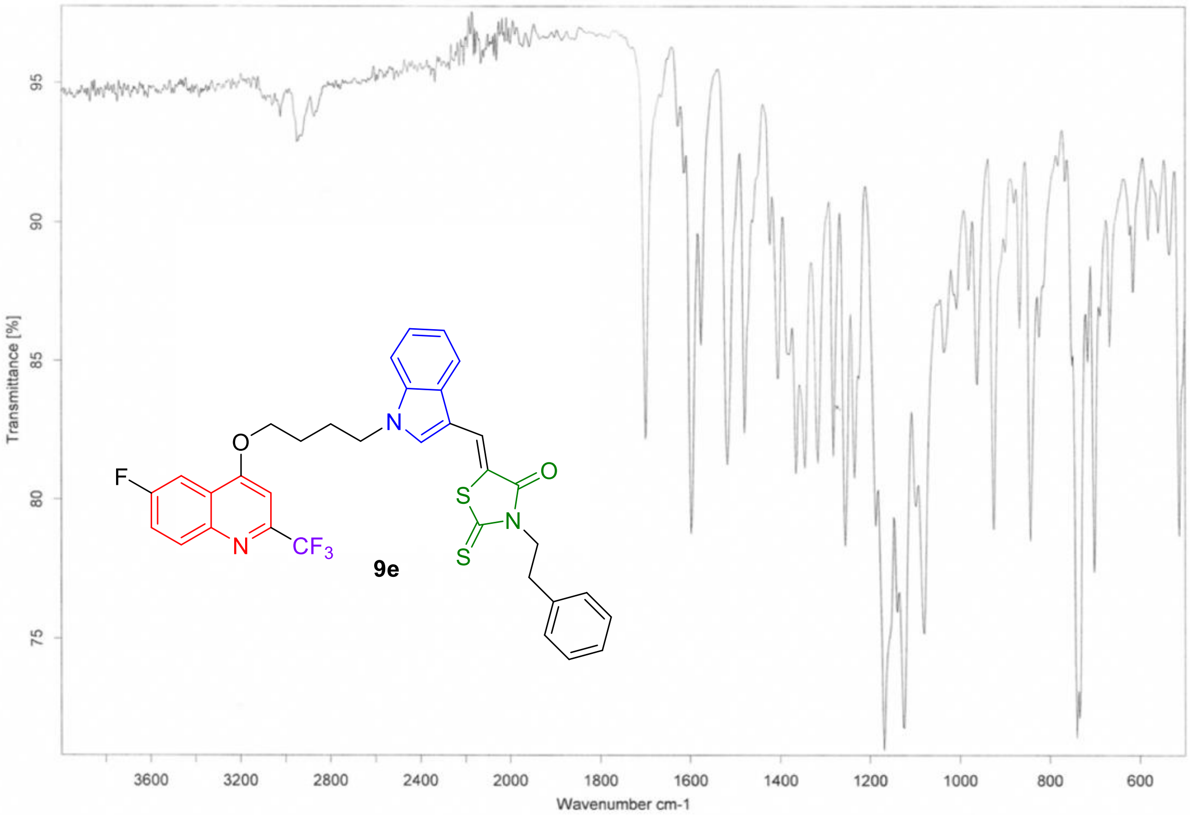

95
90
85
80
75
Transmittance [%]
3600
3200
2800
2400
2000
1800
1600
1400
1200
1000
800
600
Wavenumber cm-1
F
O
N
CF3
N
S
O
N
S

9e

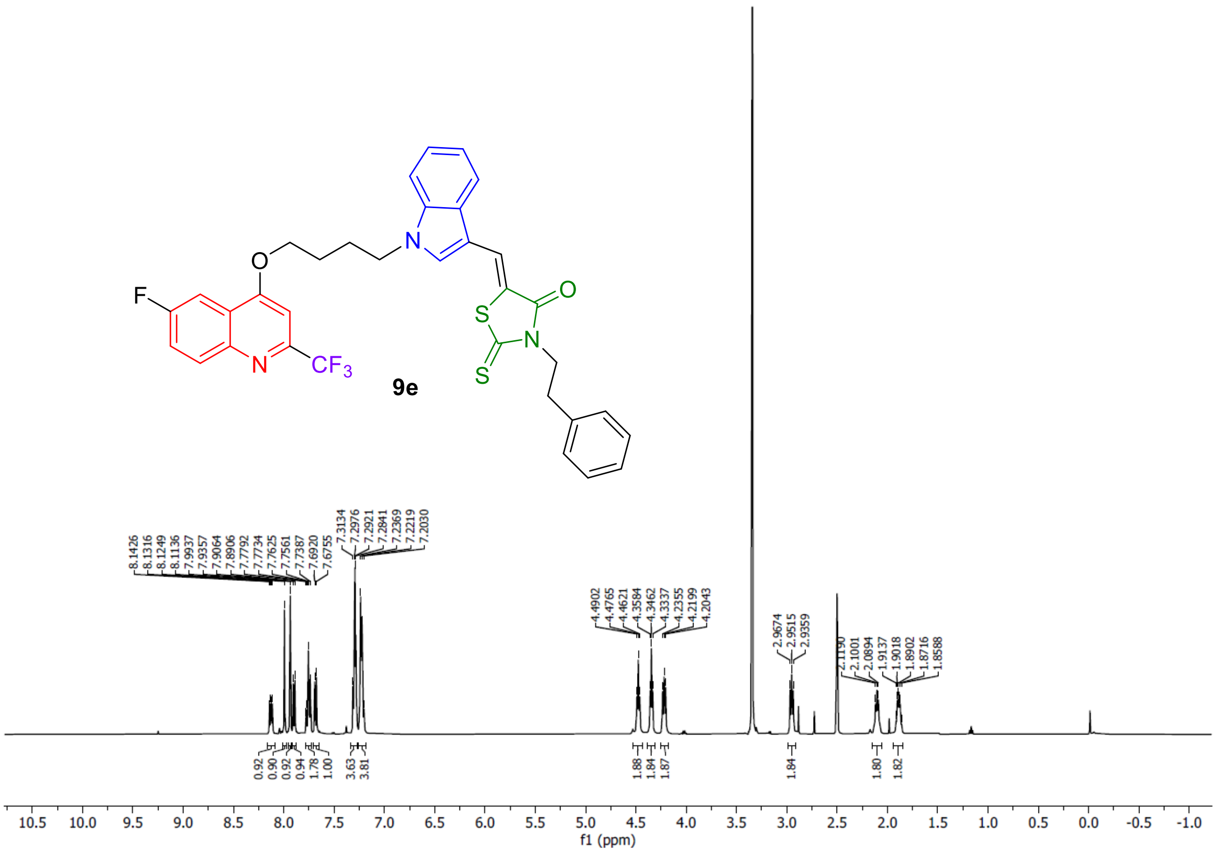

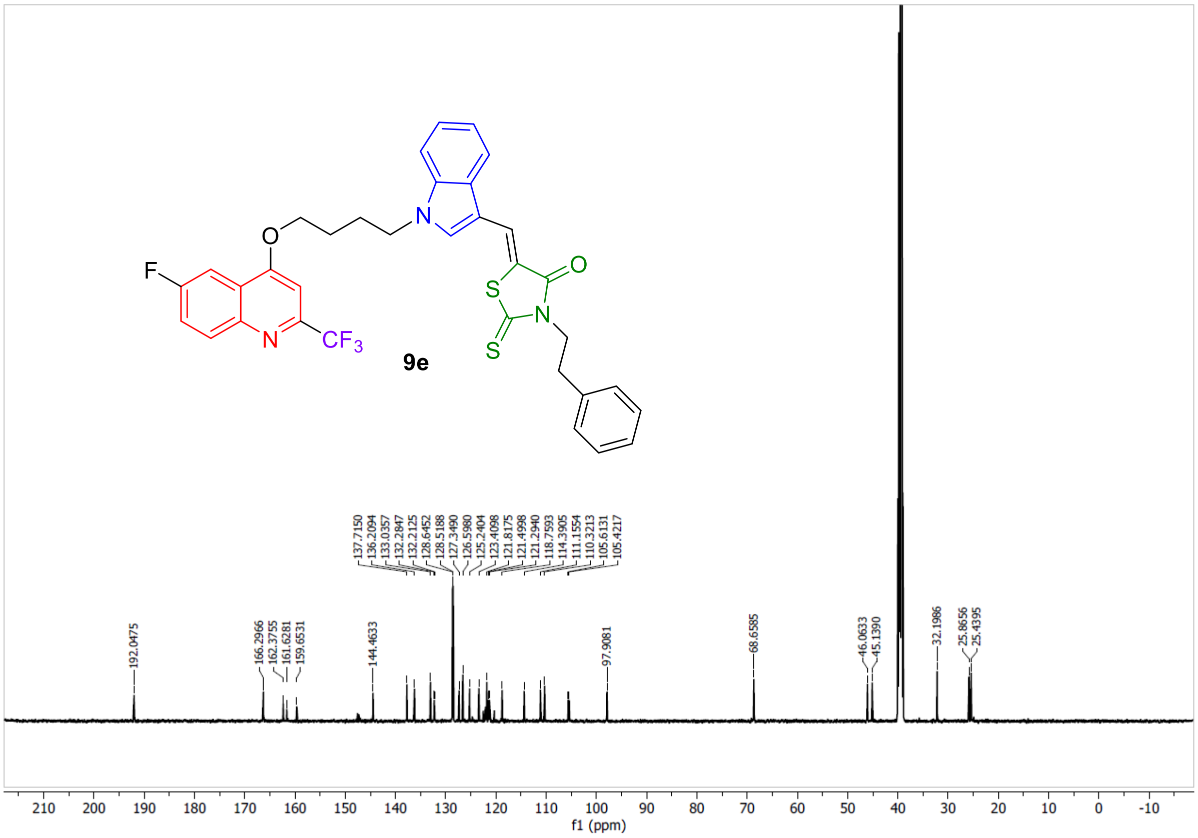

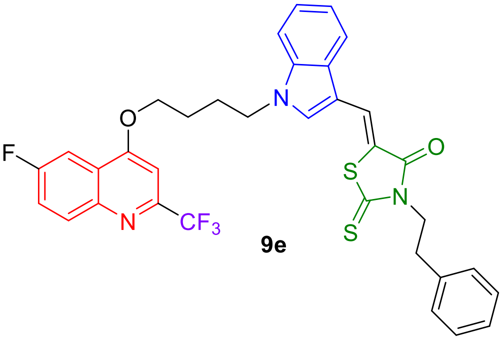

F
O
N
S
O
N
S
N
CF3

9e

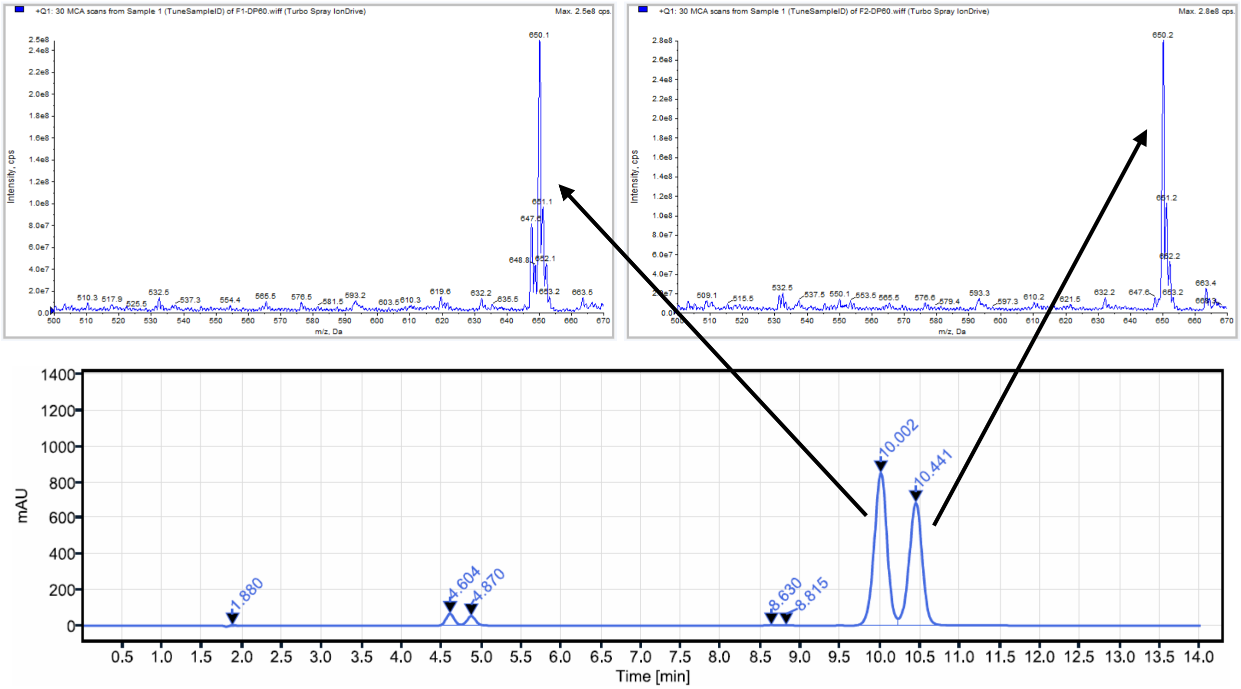

+Q1: 30 MCA scans from Sample 1 (TuneSampleID) of F1-DP60.wiff (Turbo Spray IonDrive)
Max. 2.5e8 cps.
650.1
651.1
647.5
648.8
652.1
Intensity, cps
m/z, Da
+Q1: 30 MCA scans from Sample 1 (TuneSampleID) of F2-DP60.wiff (Turbo Spray IonDrive)
Max. 2.8e8 cps.
650.2
651.2
652.2
663.4
mAU
Time [min]
1.880
4.604
4.870
8.630
8.815
10.002
10.441

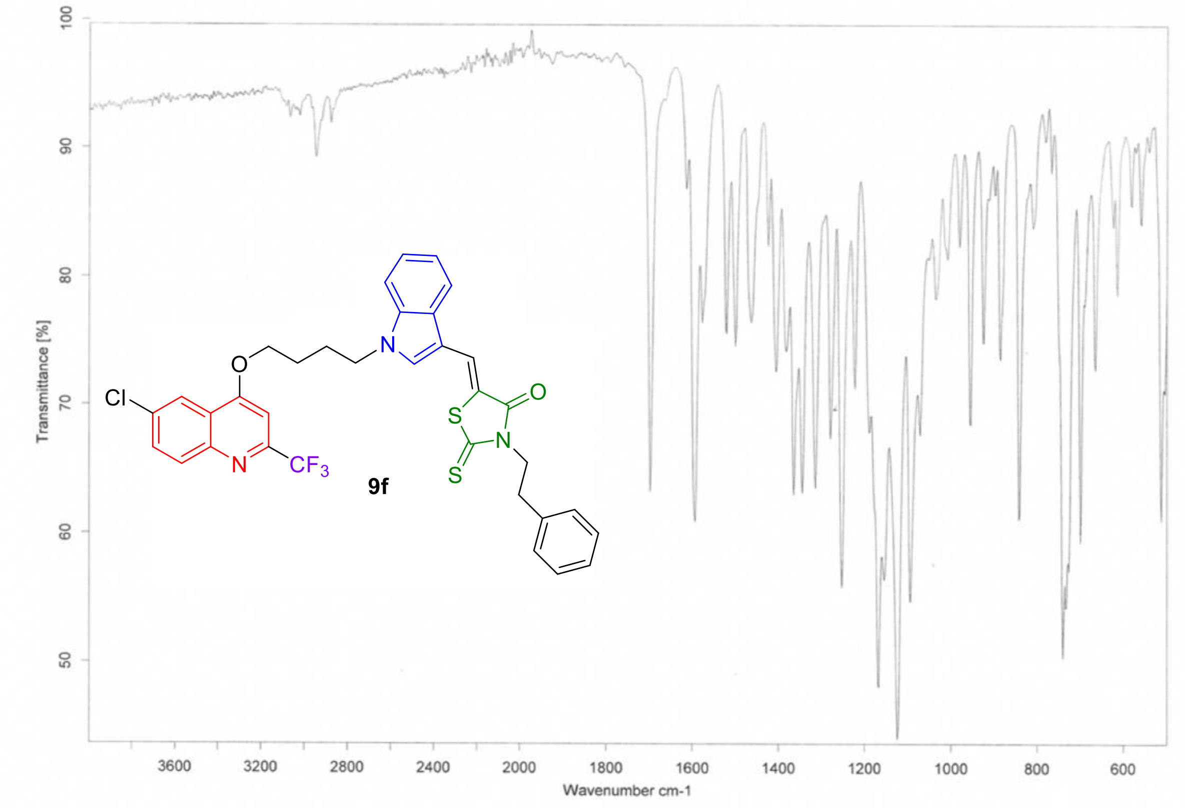
Transmittance [%]
Wavenumber cm-1
9f

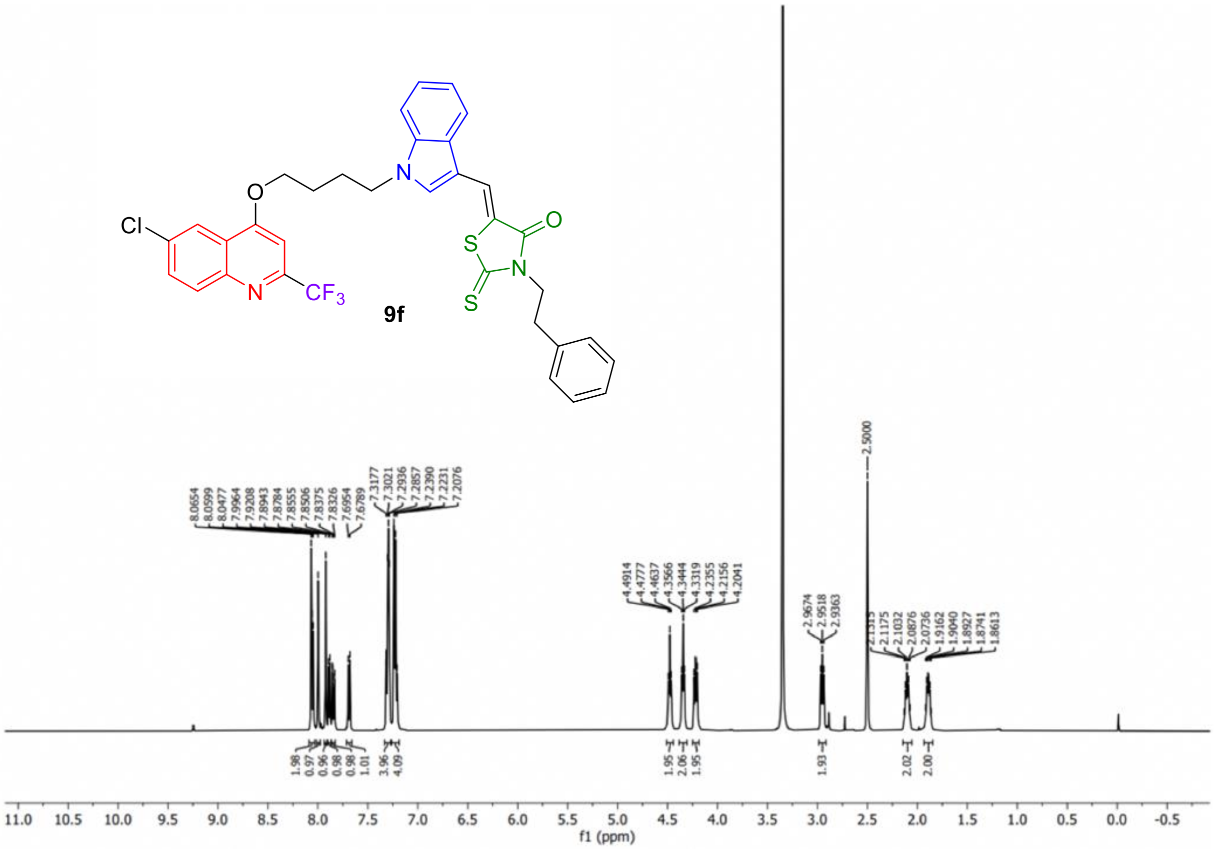
Cl
N
CF3
O
N
S
N
O
S
9f
8.0654
8.0599
8.0477
7.9964
7.9208
7.8943
7.8784
7.8555
7.8506
7.8375
7.8326
7.6954
7.6789
7.3177
7.3021
7.2936
7.2857
7.2390
7.2231
7.2076
4.4914
4.4777
4.4637
4.3566
4.3444
4.3319
4.2355
4.2156
4.2041
2.9674
2.9518
2.9363
2.5000
1.98
0.97
0.96
0.98
0.98
1.01
3.96
4.09
1.95
2.06
1.95
1.93
2.02
2.00
f1 (ppm)

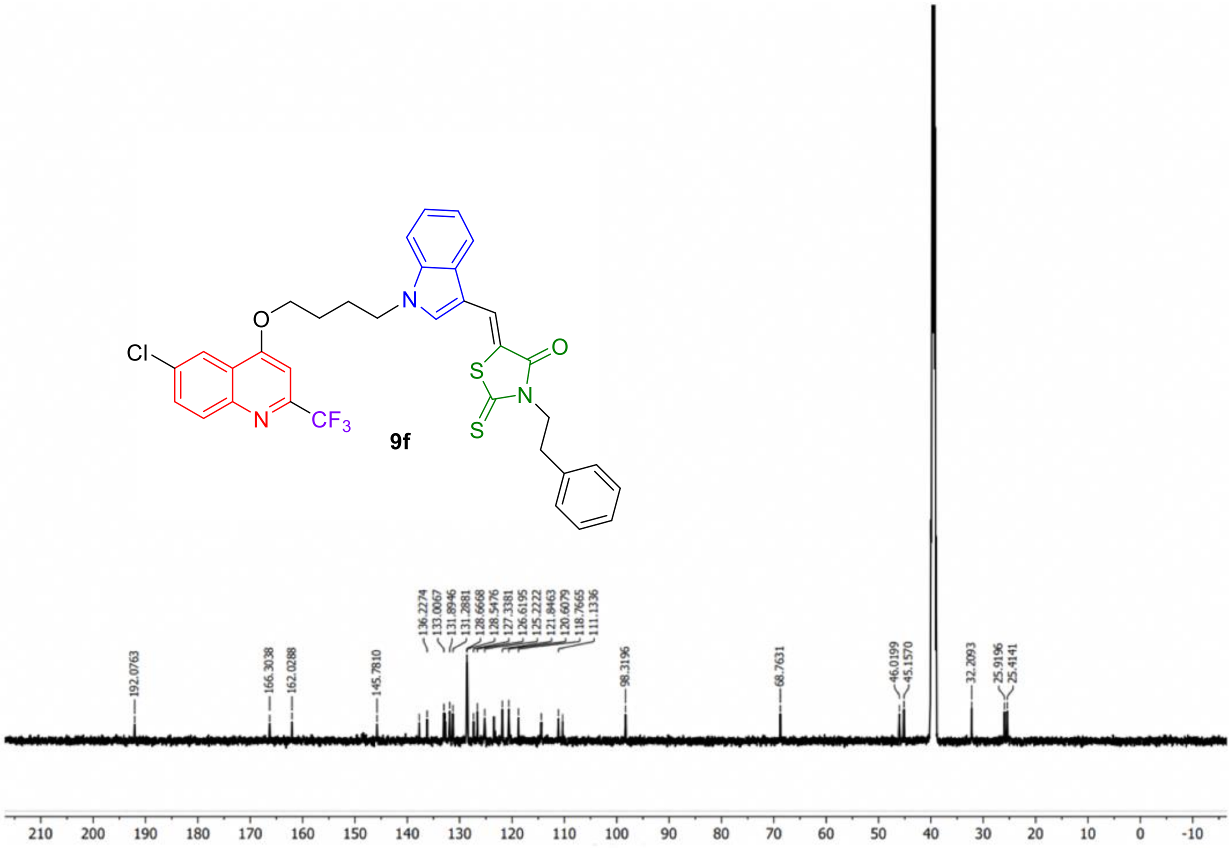
Cl
N
CF3
O
N
S
N
O
S
9f
192.0763
166.3038
162.0288
145.7810
136.2274
133.0067
131.8946
131.2881
128.6668
128.5476
127.3381
126.6195
125.2222
121.8463
120.6079
118.7665
111.1336
98.3196
68.7631
46.0199
45.1570
32.2093
25.9196
25.4141

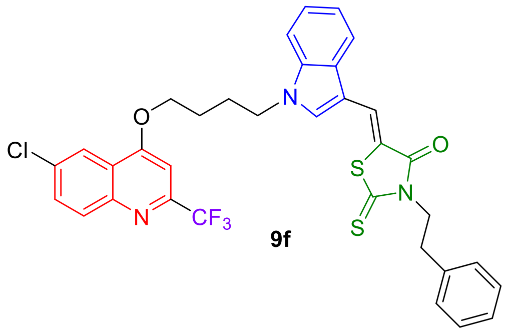
Cl
O
N
CF3
N
S
O
N
S

9f

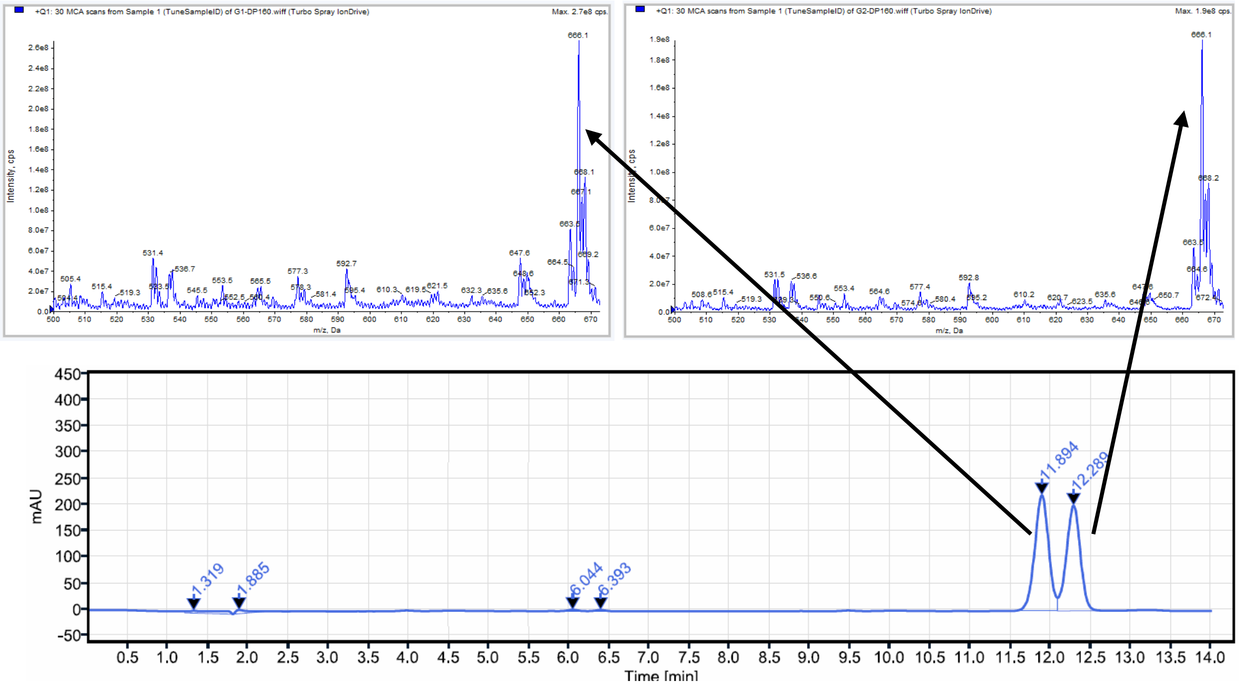
+Q1: 30 MCA scans from Sample 1 (TuneSampleID) of G1-DP160.wiff (Turbo Spray IonDrive)
Max. 2.7e8 cps.
666.1
+Q1: 30 MCA scans from Sample 1 (TuneSampleID) of G2-DP160.wiff (Turbo Spray IonDrive)
Max. 1.9e8 cps.
666.1
m/z, Da
mAU
Time [min]
1.319
1.885
6.044
6.393
11.894
12.289

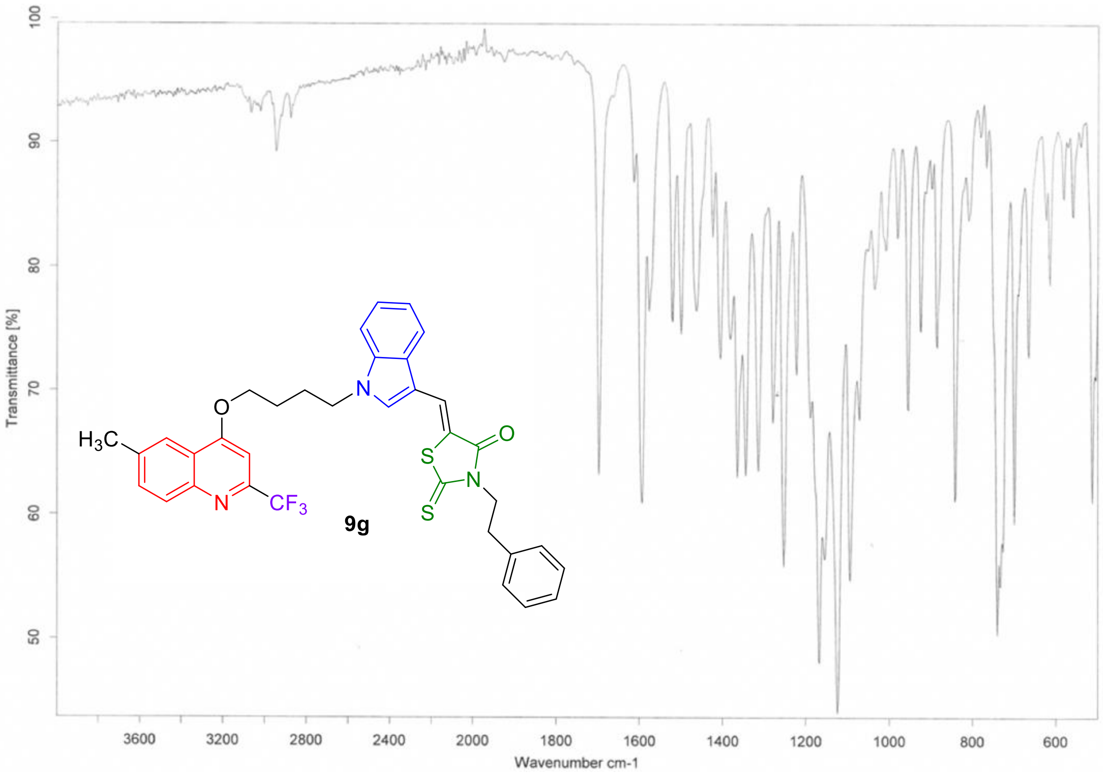
Transmittance [%]
Wavenumber cm-1
100
90
80
70
60
50
3600
3200
2800
2400
2000
1800
1600
1400
1200
1000
800
600
H3C
O
N
N
CF3
S
O
N
S
9g

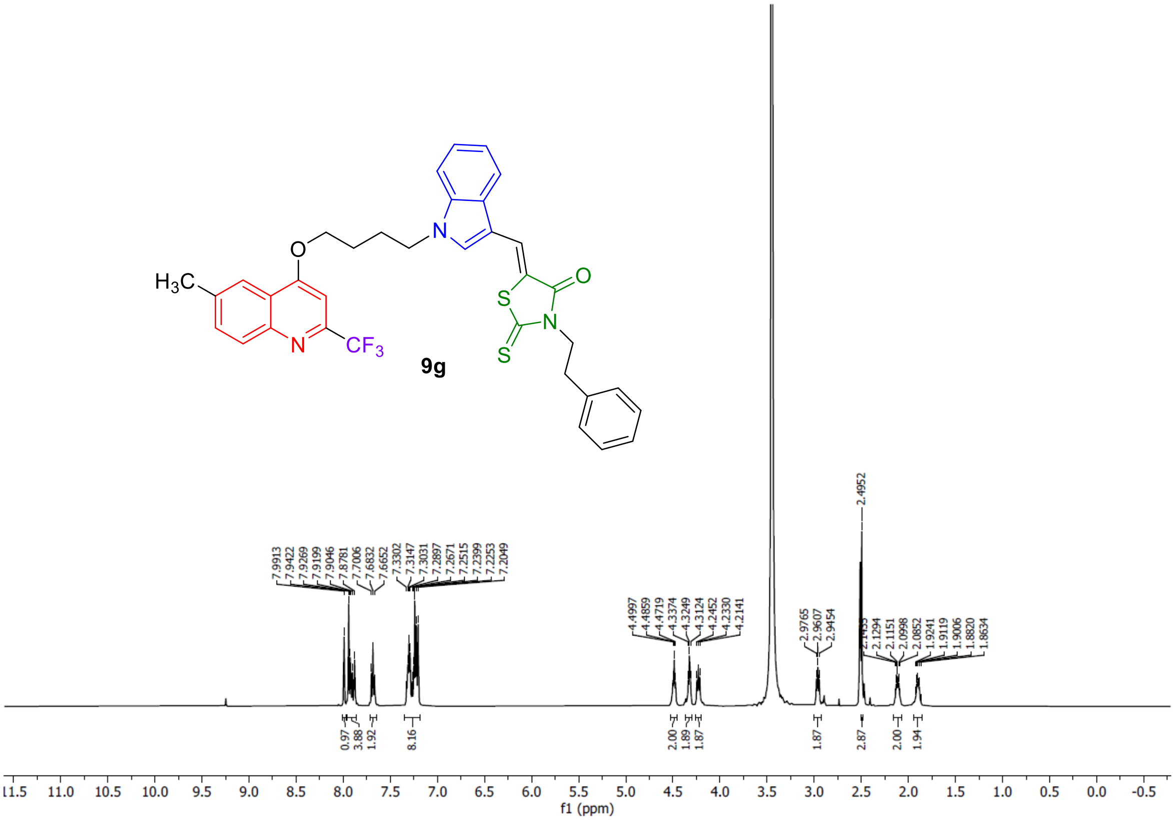
H3C
O
N
CF3
9g
S
N
O
S
7.9913
7.9422
7.9269
7.9199
7.9046
7.8781
7.7006
7.6832
7.6652
7.3302
7.3147
7.3031
7.2897
7.2671
7.2515
7.2399
7.2253
7.2049
4.4997
4.4859
4.4719
4.3374
4.3249
4.3124
4.2452
4.2330
4.2141
2.9765
2.9607
2.9454
2.4952
2.1294
2.1151
2.0998
2.0852
1.9241
1.9119
1.9006
1.8820
1.8634
0.97
3.88
1.92
8.16
2.00
1.89
1.87
1.87
2.87
2.00
1.94
f1 (ppm)

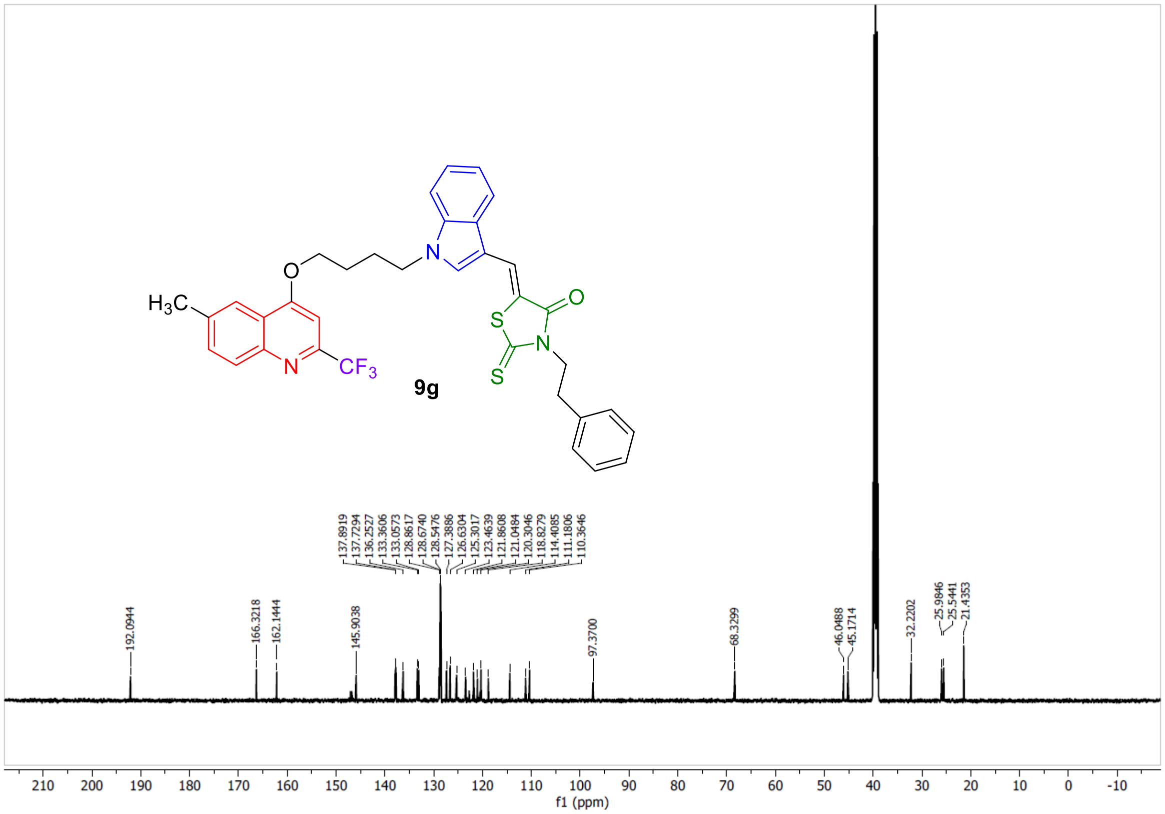
H3C
O
N
CF3
9g
S
N
O
S
192.0944
166.3218
162.1444
145.9038
137.8919
137.7294
136.2527
133.3606
133.0573
128.8617
128.6740
128.5476
127.3886
126.6304
125.3017
123.4639
121.8608
121.0484
120.3046
118.8279
114.4085
111.1806
110.3646
97.3700
68.3299
46.0488
45.1714
32.2202
25.9846
25.5441
21.4353
f1 (ppm)

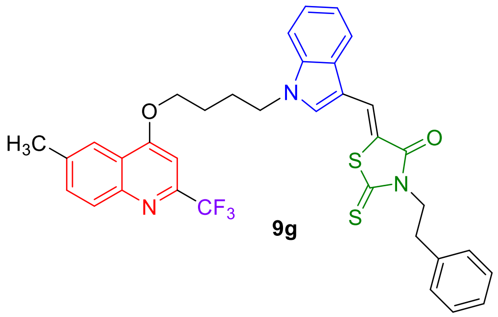

9g

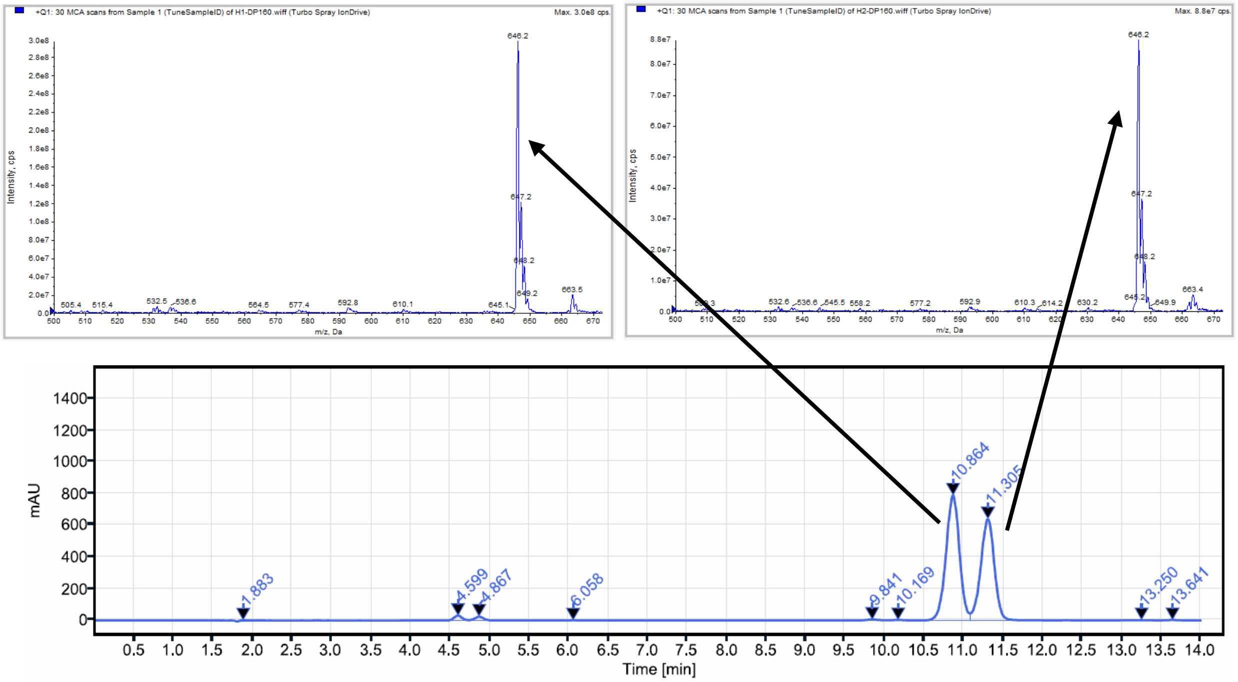

+Q1: 30 MCA scans from Sample 1 (TuneSampleID) of H1-DP160.wiff (Turbo Spray IonDrive)
Max. 3.0e8 cps.
Intensity, cps
m/z, Da
646.2
647.2
648.2
649.2
663.5
+Q1: 30 MCA scans from Sample 1 (TuneSampleID) of H2-DP160.wiff (Turbo Spray IonDrive)
Max. 8.8e7 cps.
Intensity, cps
m/z, Da
646.2
647.2
648.2
663.4
mAU
Time [min]
1.883
4.599
4.867
6.058
9.841
10.169
10.864
11.305
13.250
13.641

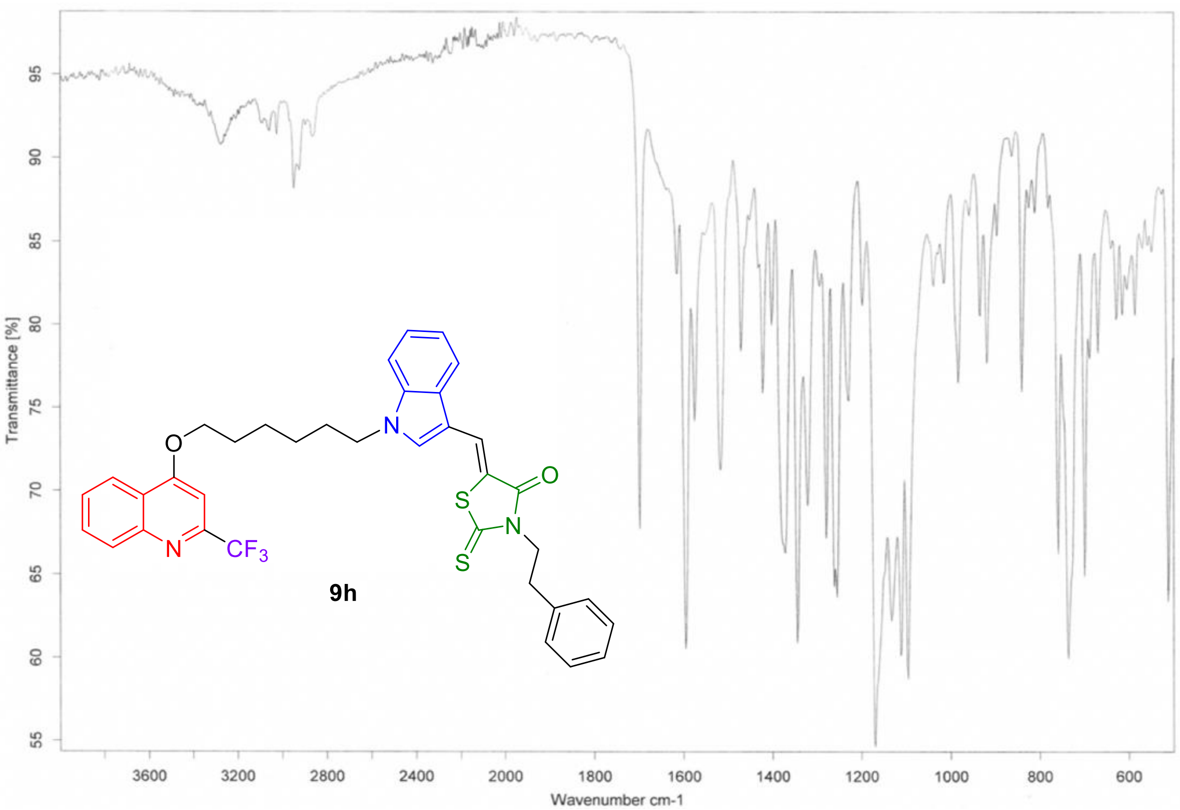
Transmittance [%]
95
90
85
80
75
70
65
60
55
3600
3200
2800
2400
2000
1800
1600
1400
1200
1000
800
600
Wavenumber cm-1
O
N
CF3
N
S
O
N
S
9h

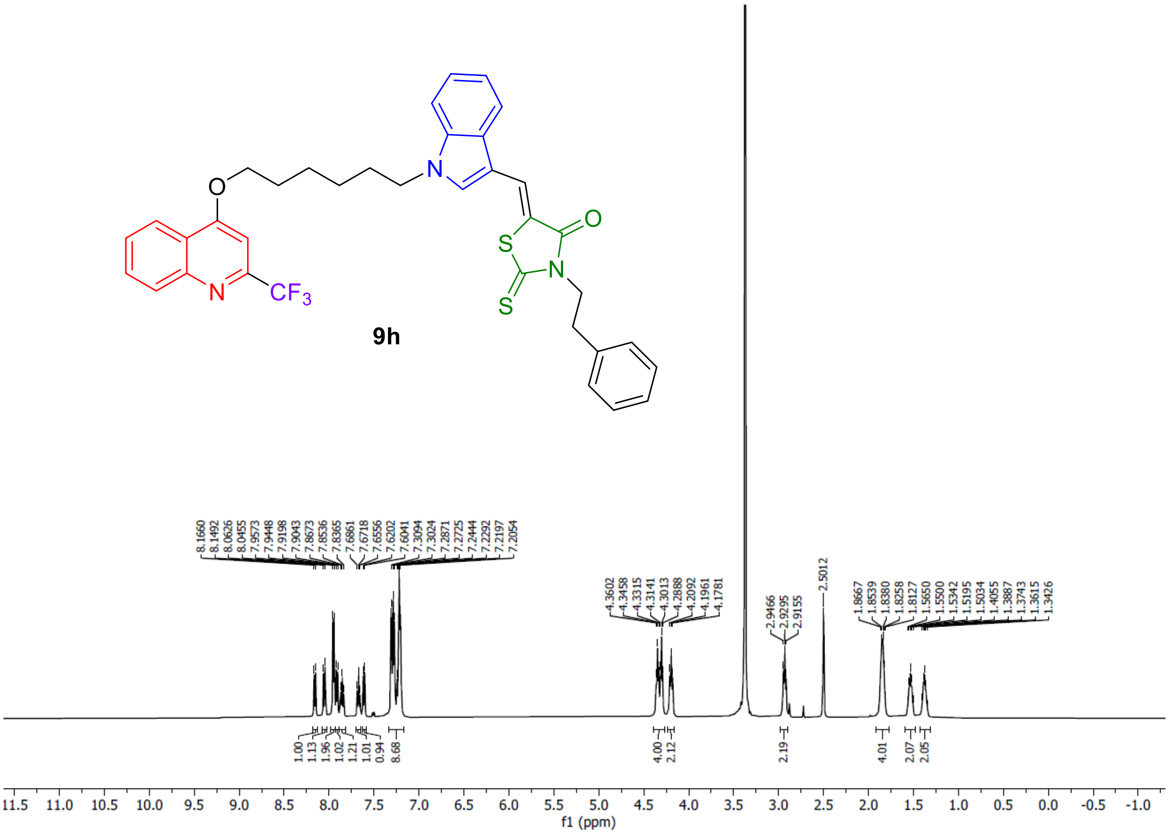
9h

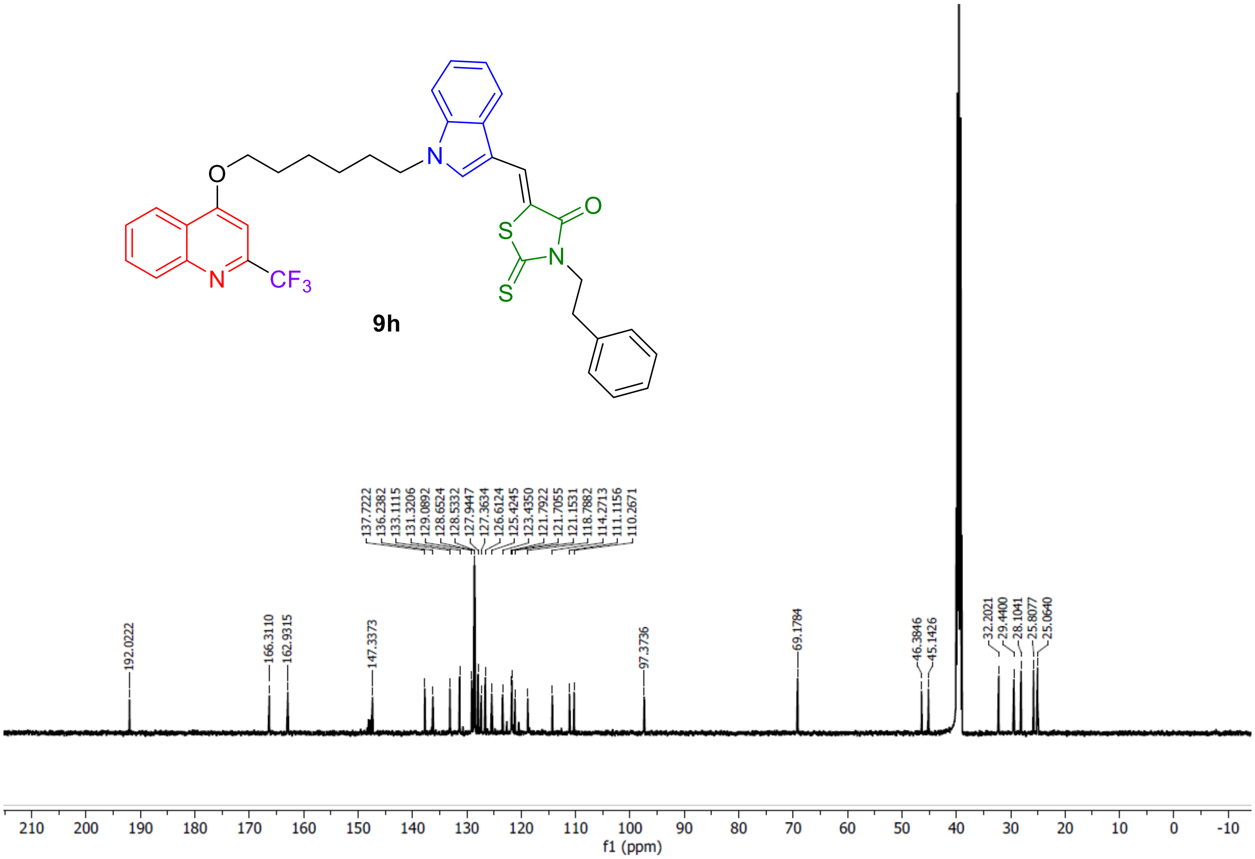
9h

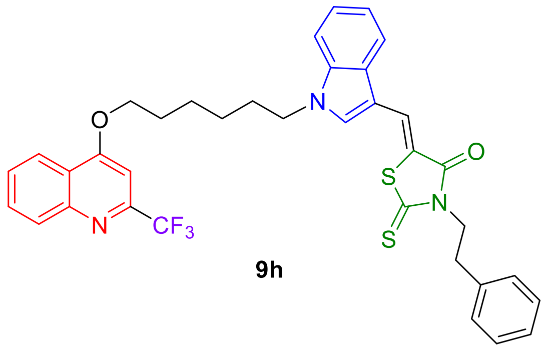

9h

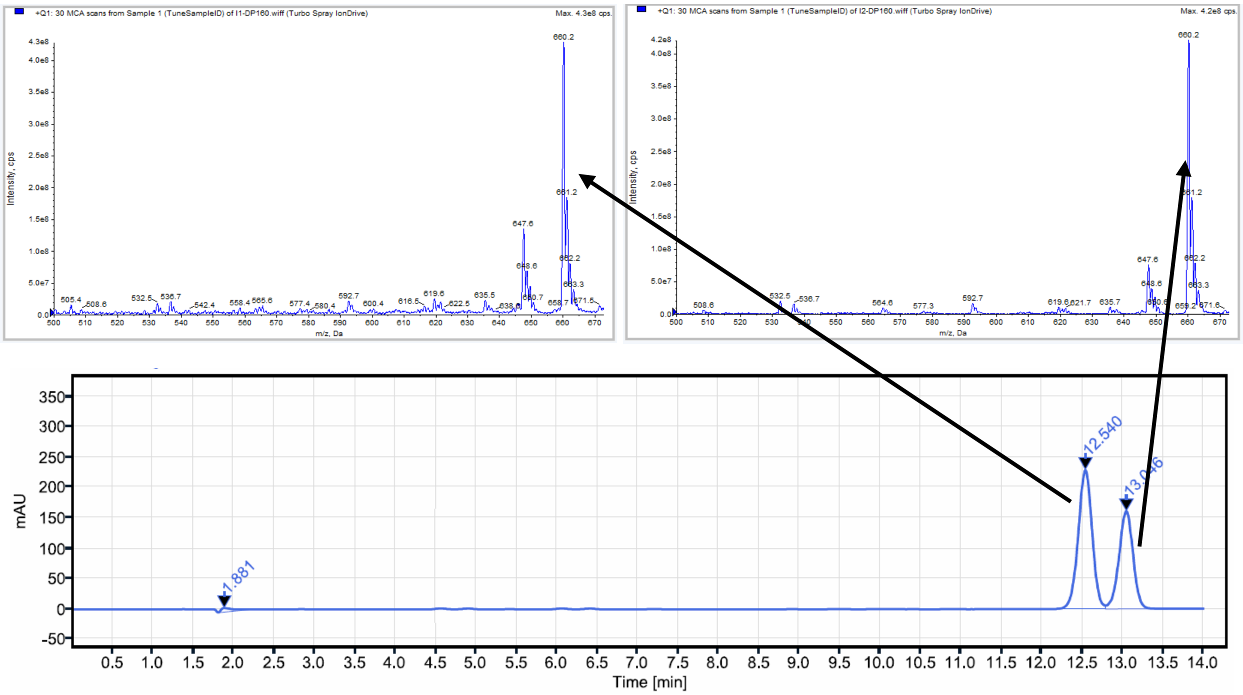

+Q1: 30 MCA scans from Sample 1 (TuneSampleID) of I1-DP160.wiff (Turbo Spray IonDrive)
Max. 4.3e8 cps.
+Q1: 30 MCA scans from Sample 1 (TuneSampleID) of I2-DP160.wiff (Turbo Spray IonDrive)
Max. 4.2e8 cps.
Intensity, cps
m/z, Da
660.2
647.6
mAU
Time [min]
12.540
1.881

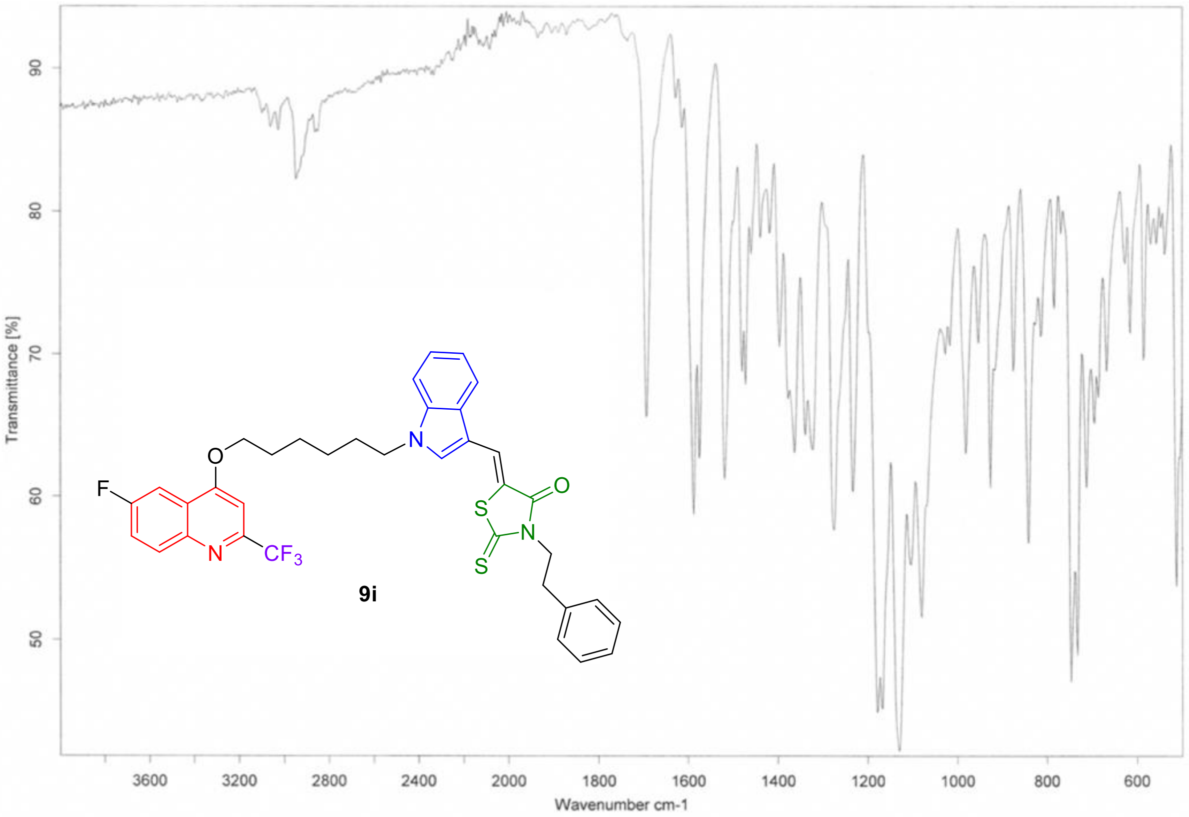

Transmittance [%]
90
80
70
60
50
3600
3200
2800
2400
2000
1800
1600
1400
1200
1000
800
600
Wavenumber cm-1
F
O
N
CF3
S
O
N
S
9i

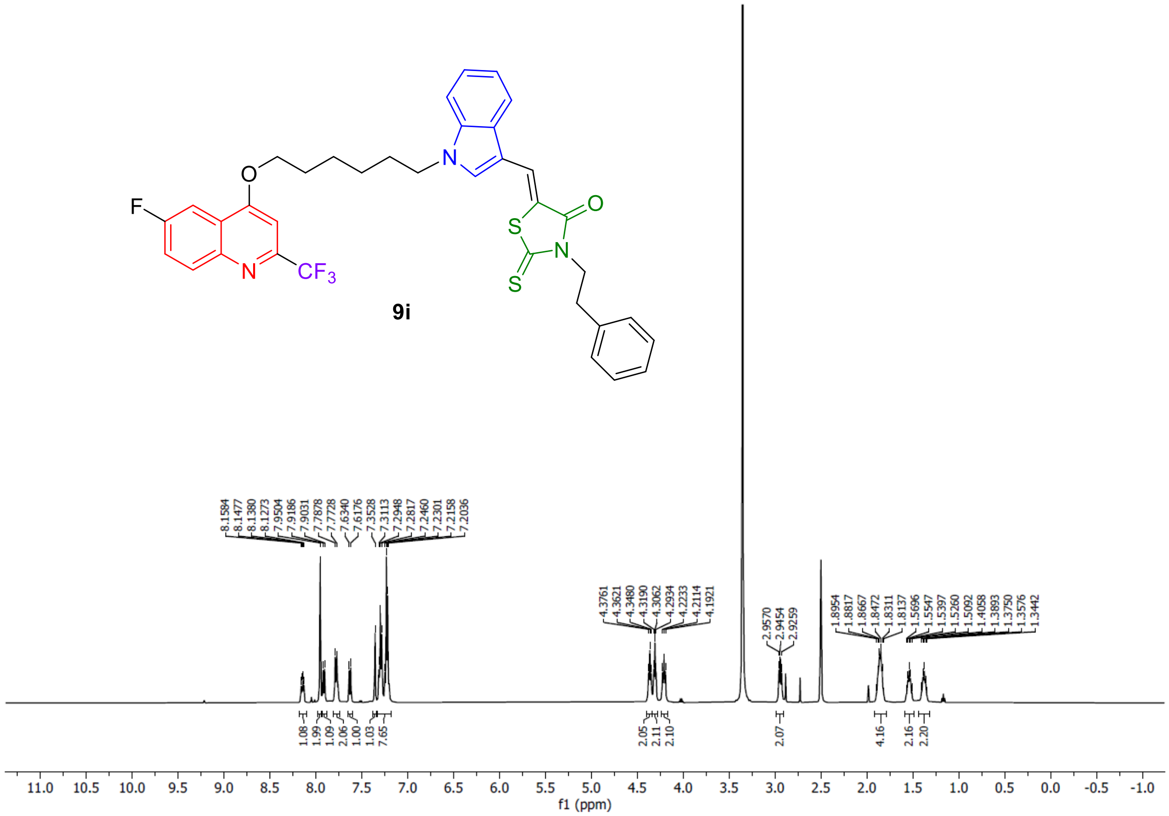

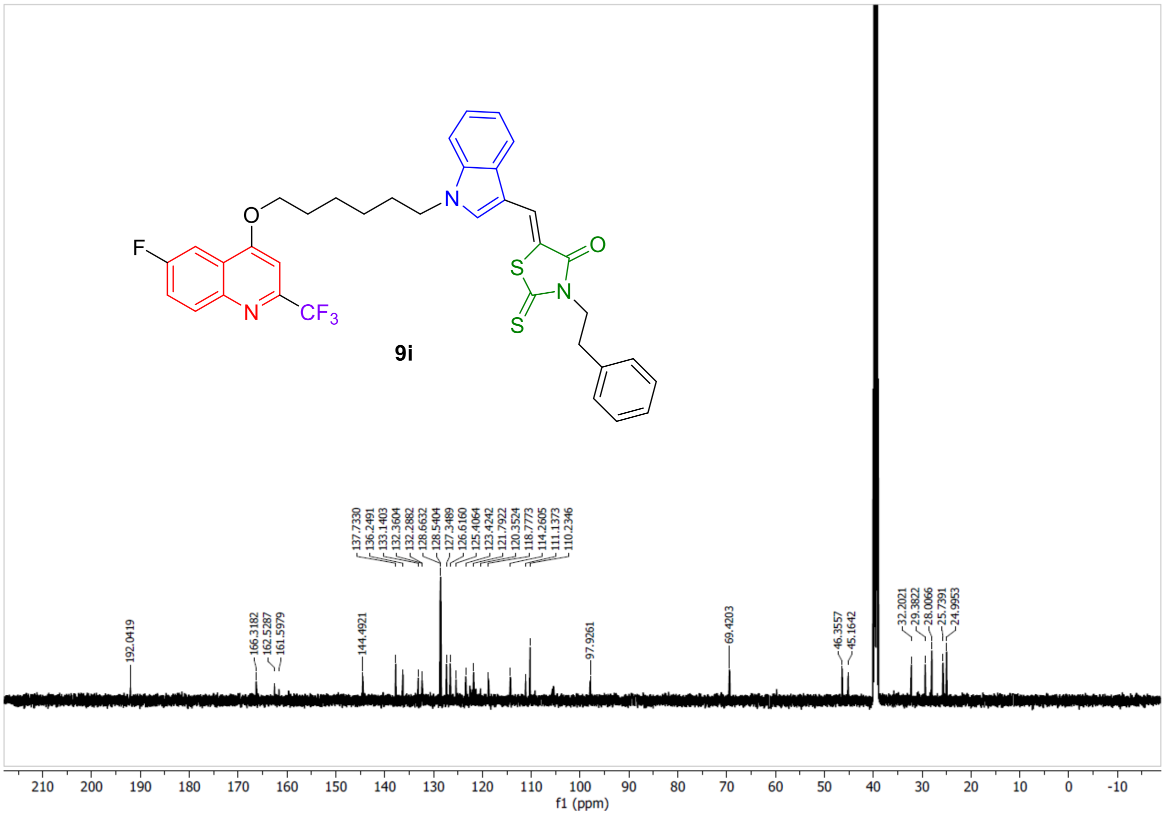

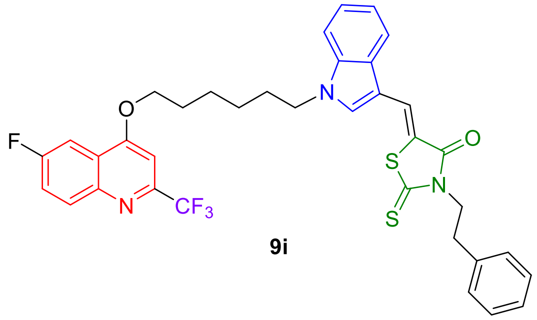
F
O
N
CF_3
N
S
O
N
S

9i

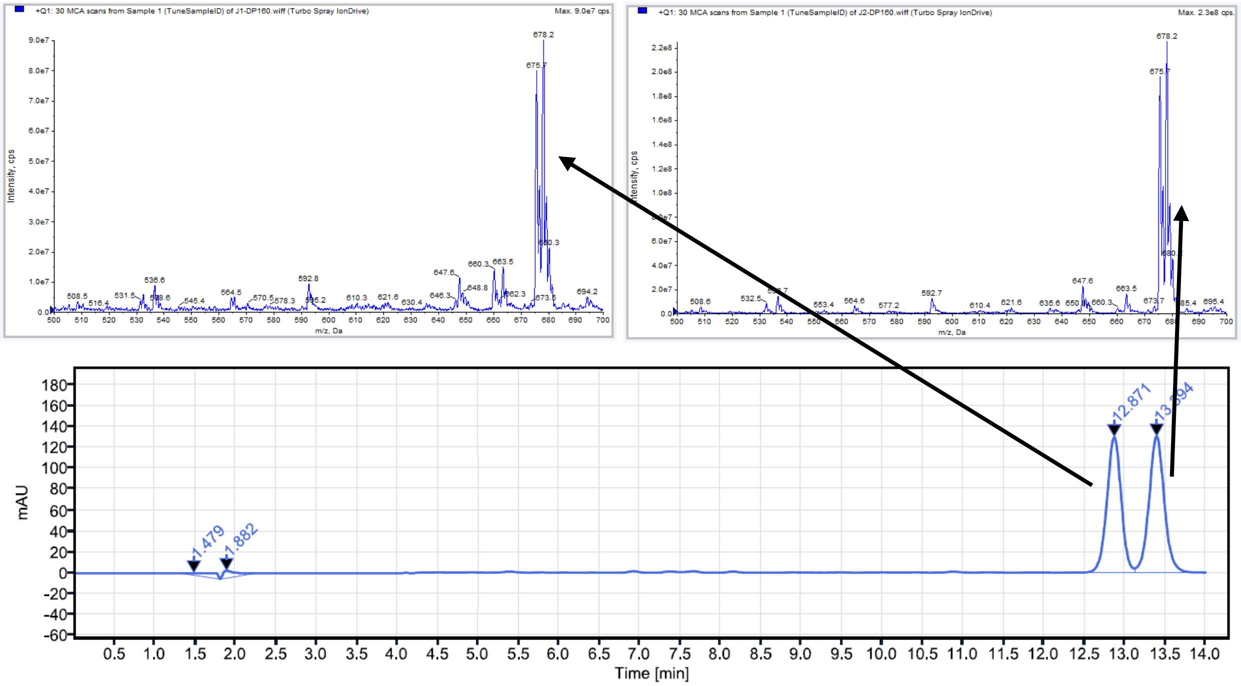
+Q1: 30 MCA scans from Sample 1 (TuneSampleID) of J1-DP160.wiff (Turbo Spray IonDrive)
Max. 9.0e7 cps.
678.2
675.7
Intensity, cps
m/z, Da
+Q1: 30 MCA scans from Sample 1 (TuneSampleID) of J2-DP160.wiff (Turbo Spray IonDrive)
Max. 2.3e8 cps.
678.2
675.7
Intensity, cps
m/z, Da
1.479
1.882
12.871
13.394
mAU
Time [min]

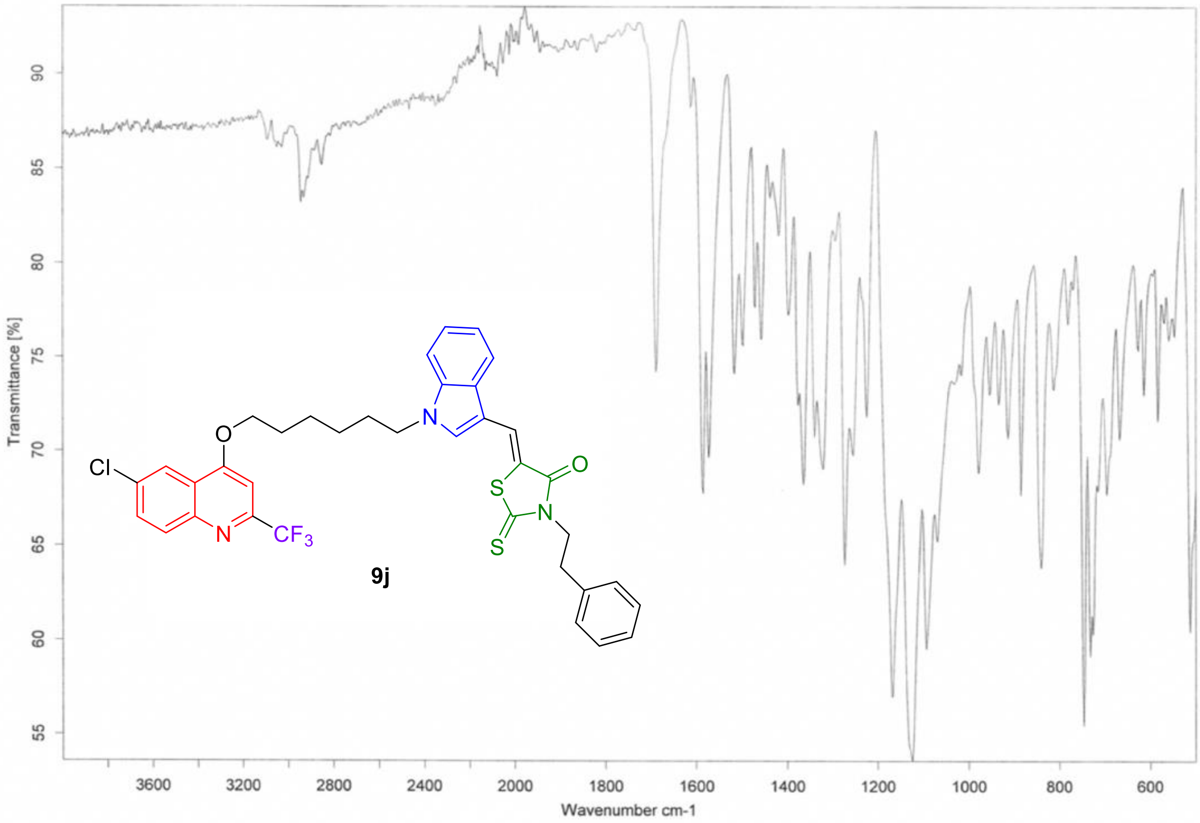

Transmittance [%]
90
85
80
75
70
65
60
55
3600
3200
2800
2400
2000
1800
1600
1400
1200
1000
800
600
Wavenumber cm-1
Cl
O
N
CF3
N
S
O
N
S
9j

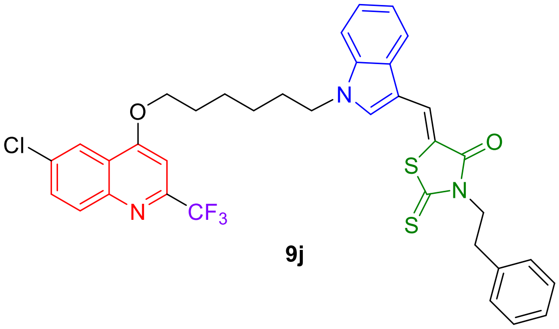
Cl
O
N
CF3
N
S
O
N
S

9j

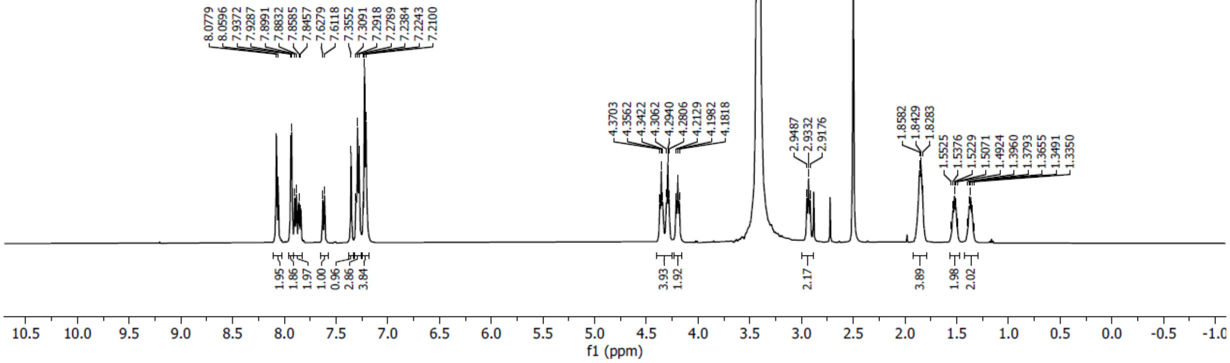
8.0779
8.0596
7.9372
7.9287
7.8991
7.8832
7.8585
7.8457
7.6279
7.6118
7.3552
7.3091
7.2918
7.2789
7.2384
7.2243
7.2100
4.3703
4.3562
4.3422
4.3062
4.2940
4.2806
4.2129
4.1982
4.1818
2.9487
2.9332
2.9176
1.8582
1.8429
1.8283
1.5525
1.5376
1.5229
1.5071
1.4924
1.3960
1.3793
1.3655
1.3491
1.3350
1.95
1.86
1.97
1.00
0.96
2.86
3.84
3.93
1.92
2.17
3.89
1.98
2.02
f1 (ppm)

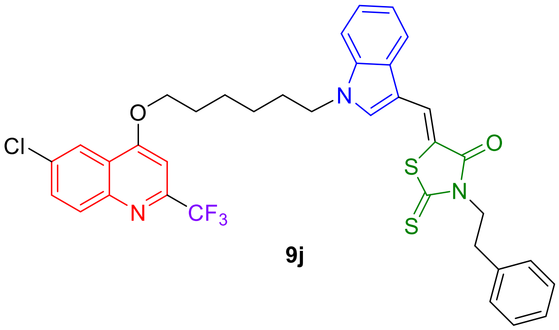
Cl
O
N
CF3
N
S
S
N
O

9j

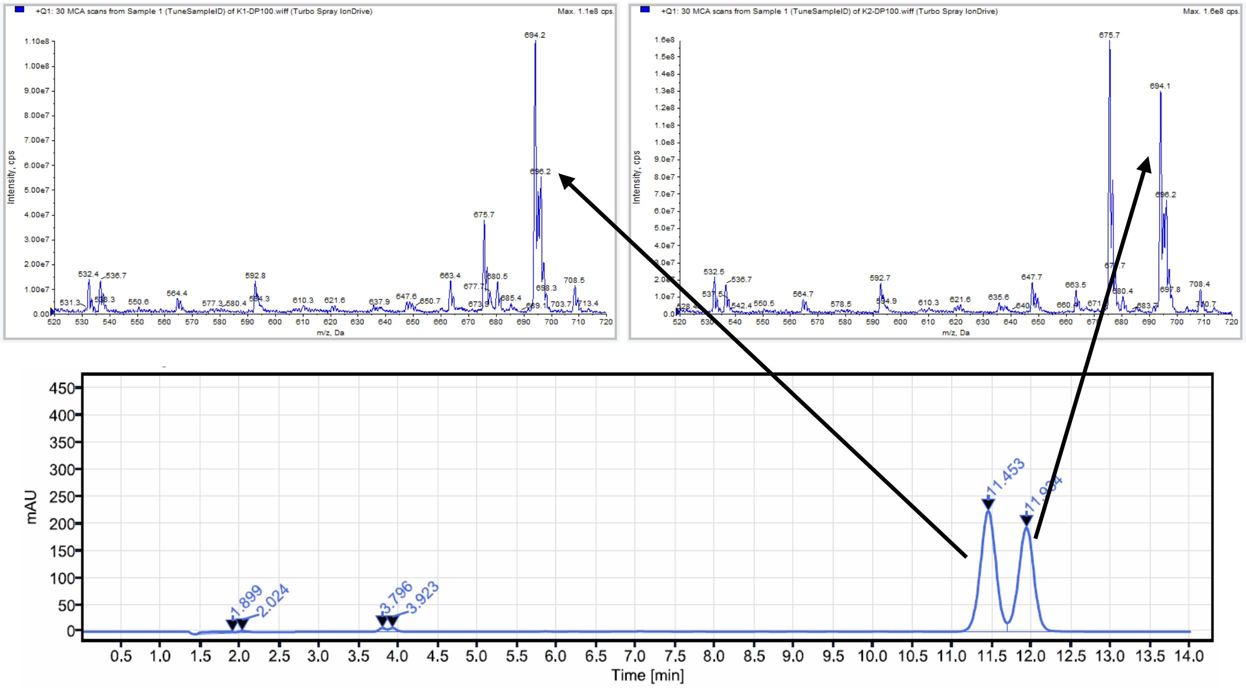
+Q1: 30 MCA scans from Sample 1 (TuneSampleID) of K1-DP100.wiff (Turbo Spray IonDrive)
Max. 1.1e8 cps.
694.2
696.2
675.7
663.4
592.8
532.4
536.7
564.4
708.5
+Q1: 30 MCA scans from Sample 1 (TuneSampleID) of K2-DP100.wiff (Turbo Spray IonDrive)
Max. 1.6e8 cps.
675.7
694.1
696.2
647.7
663.5
592.7
532.5
536.7
564.7
708.4
Intensity, cps
m/z, Da
11.453
1.899
2.024
3.796
3.923
mAU
Time [min]

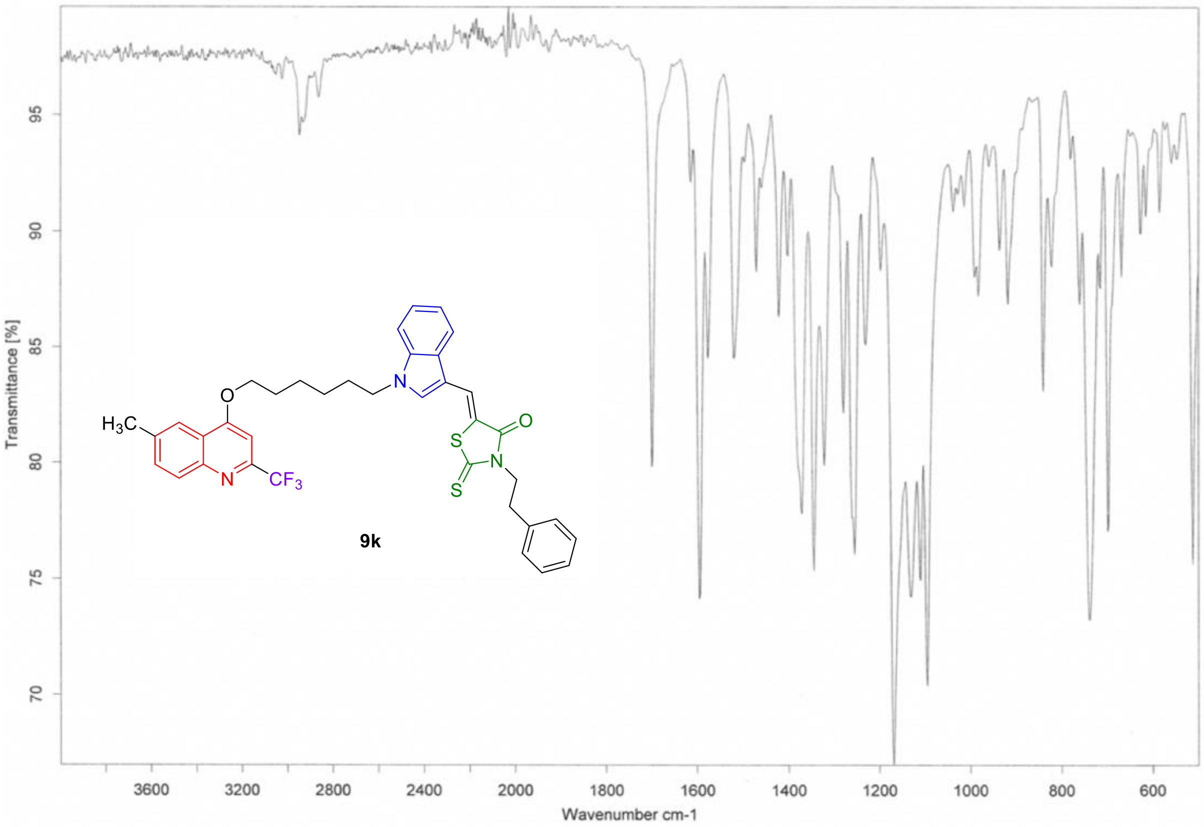
Transmittance [%]
Wavenumber cm-1
3600
3200
2800
2400
2000
1800
1600
1400
1200
1000
800
600
95
90
85
80
75
70
H3C
O
N
N
CF3
S
O
N
S
9k

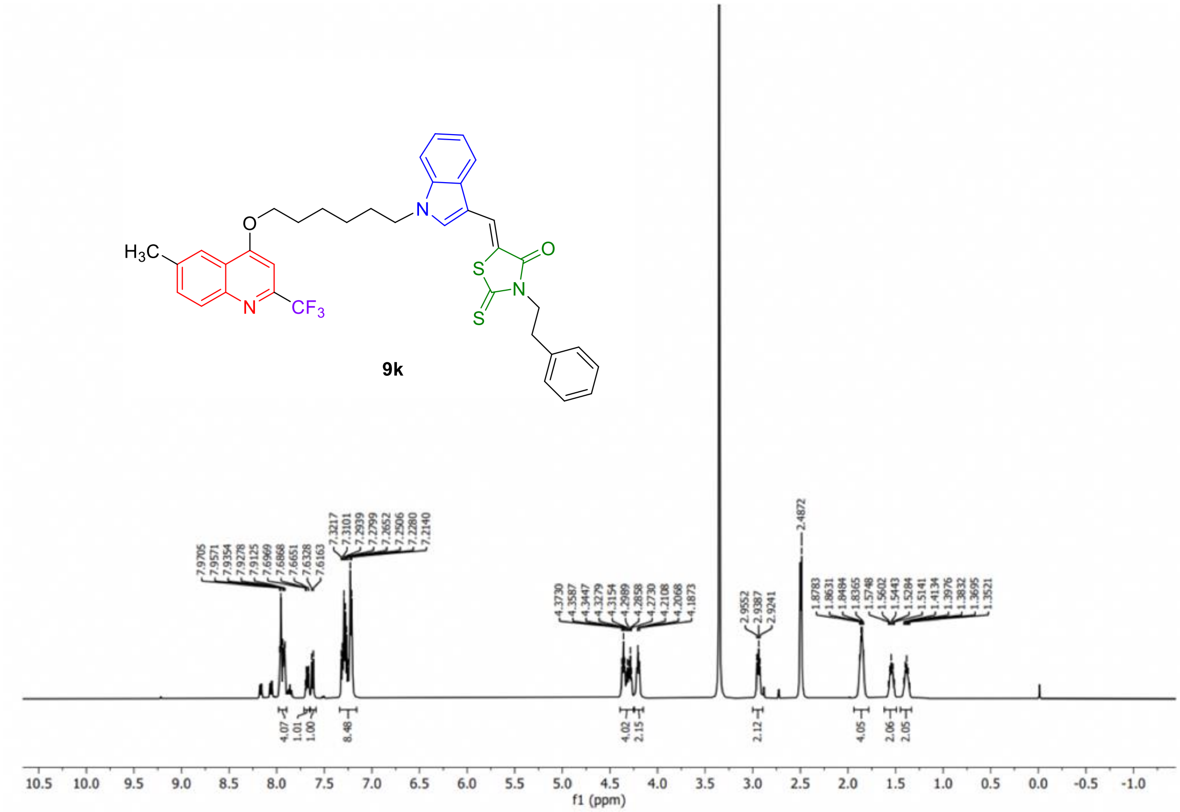
H3C
O
N
CF3
N
S
O
S
N
9k
7.9705
7.9571
7.9354
7.9278
7.9125
7.6969
7.6868
7.6651
7.6328
7.6163
7.3217
7.3101
7.2939
7.2799
7.2652
7.2506
7.2280
7.2140
4.3730
4.3587
4.3447
4.3279
4.3154
4.2989
4.2858
4.2730
4.2108
4.2068
4.1873
2.9552
2.9387
2.9241
2.4872
1.8783
1.8631
1.8484
1.8365
1.5748
1.5602
1.5543
1.5284
1.5141
1.4134
1.3976
1.3832
1.3695
1.3521
4.07
1.01
1.00
8.48
4.02
2.15
2.12
4.05
2.06
2.05
f1 (ppm)

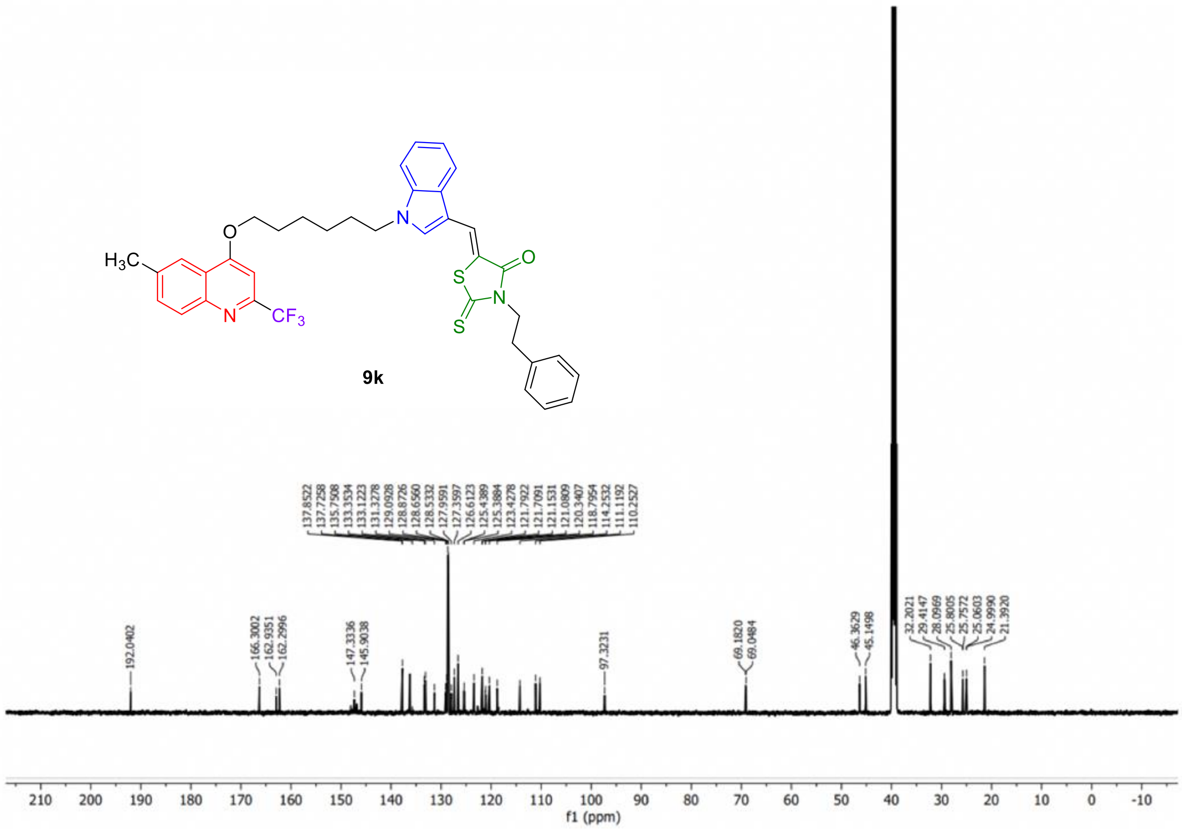
H3C
O
N
CF3
N
S
O
S
N
9k
192.0402
166.3002
162.9351
162.2996
147.3336
145.9038
137.8522
137.7258
135.7508
133.3534
133.1223
131.3278
129.0928
128.8726
128.6560
128.5332
127.9591
127.3597
126.6123
125.4389
125.3884
123.4278
121.7922
121.7091
121.1531
121.0809
120.3407
118.7954
114.2532
111.1192
110.2527
97.3231
69.1820
69.0484
46.3629
45.1498
32.2021
29.4147
28.0969
25.8005
25.7572
25.0603
24.9990
21.3920
f1 (ppm)

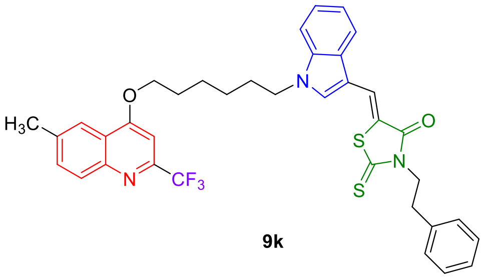

O
N
H3C
N
CF3
S
S
N
O

9k

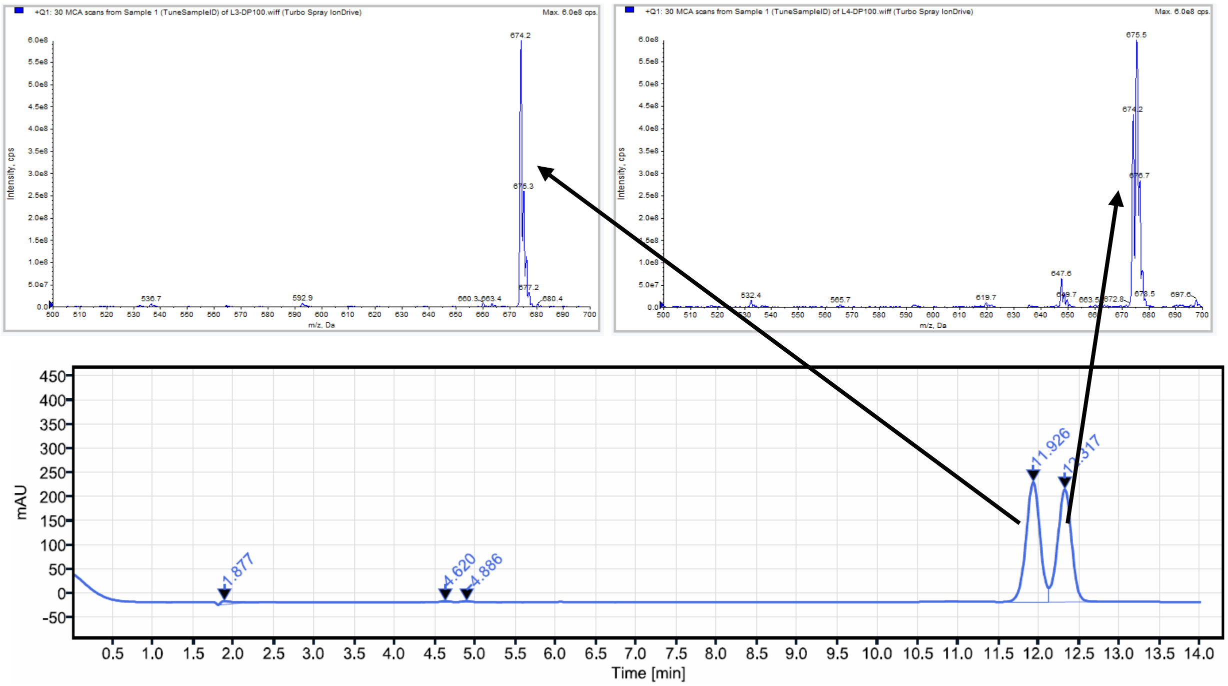

+Q1: 30 MCA scans from Sample 1 (TuneSampleID) of L3-DP100.wiff (Turbo Spray IonDrive)
Max. 6.0e8 cps.
Intensity, cps
m/z, Da
674.2
675.3
536.7
592.9
660.3
663.4
677.2
680.4
+Q1: 30 MCA scans from Sample 1 (TuneSampleID) of L4-DP100.wiff (Turbo Spray IonDrive)
Max. 6.0e8 cps.
675.5
674.2
676.7
647.6
532.4
565.7
619.7
697.6
mAU
Time [min]
1.877
4.620
4.886
11.926
12.317

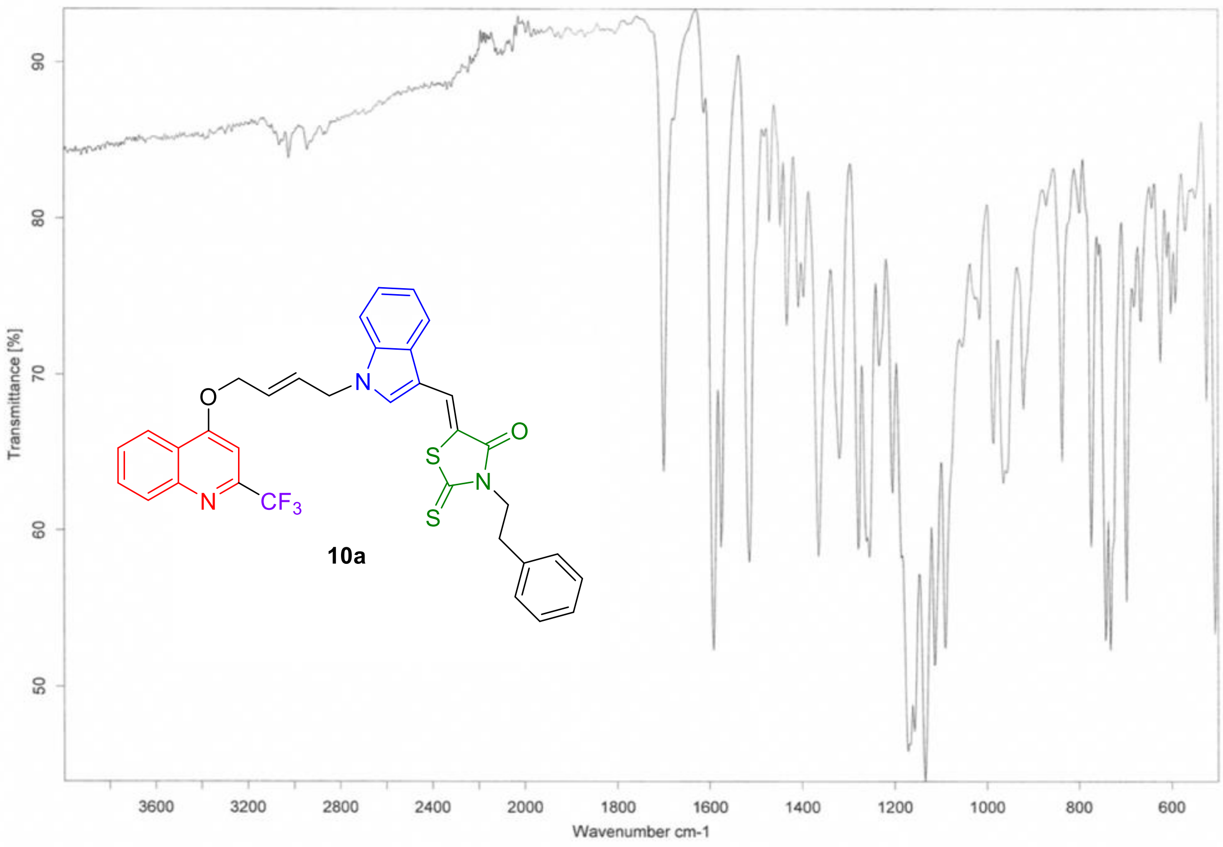
Transmittance [%]
90
80
70
60
50
3600
3200
2800
2400
2000
1800
1600
1400
1200
1000
800
600
Wavenumber cm-1
O
N
CF3
N
S
O
N
S
10a

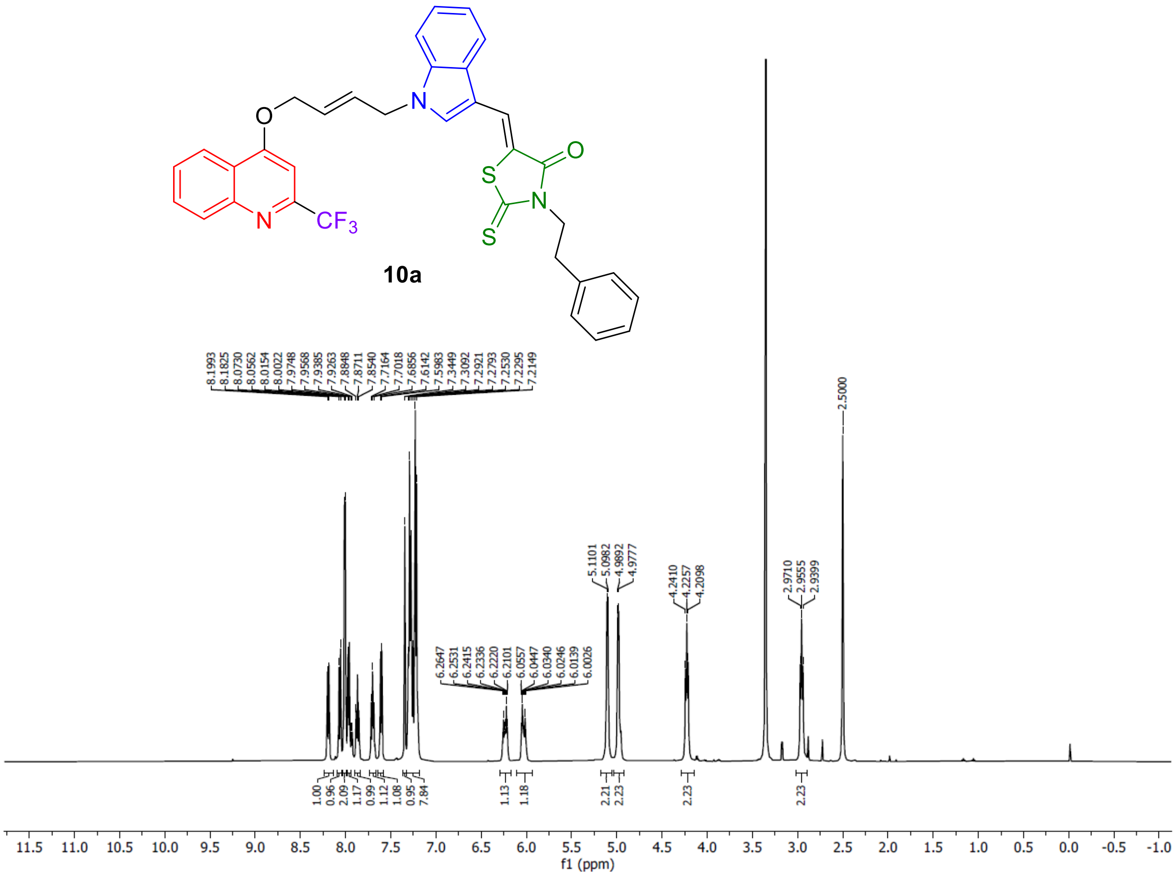
O
N
N
CF3
S
S
O
N
10a
8.1993 8.1825 8.0730 8.0562 8.0154 8.0022 7.9748 7.9568 7.9385 7.9263 7.8848 7.8711 7.8540 7.7164 7.7018 7.6856 7.6142 7.5983 7.3449 7.3092 7.2921 7.2793 7.2530 7.2295 7.2149
6.2647 6.2531 6.2415 6.2336 6.2220 6.2101 6.0557 6.0447 6.0340 6.0246 6.0139 6.0026
5.1101 5.0982 4.9892 4.9777
4.2410 4.2257 4.2098
2.9710 2.9555 2.9399
2.5000
1.00 0.96 2.09 1.17 0.99 1.12 1.08 0.95 7.84
1.13 1.18
2.21 2.23
2.23
2.23
11.5 11.0 10.5 10.0 9.5 9.0 8.5 8.0 7.5 7.0 6.5 6.0 5.5 5.0 4.5 4.0 3.5 3.0 2.5 2.0 1.5 1.0 0.5 0.0 -0.5 -1.0
f1 (ppm)

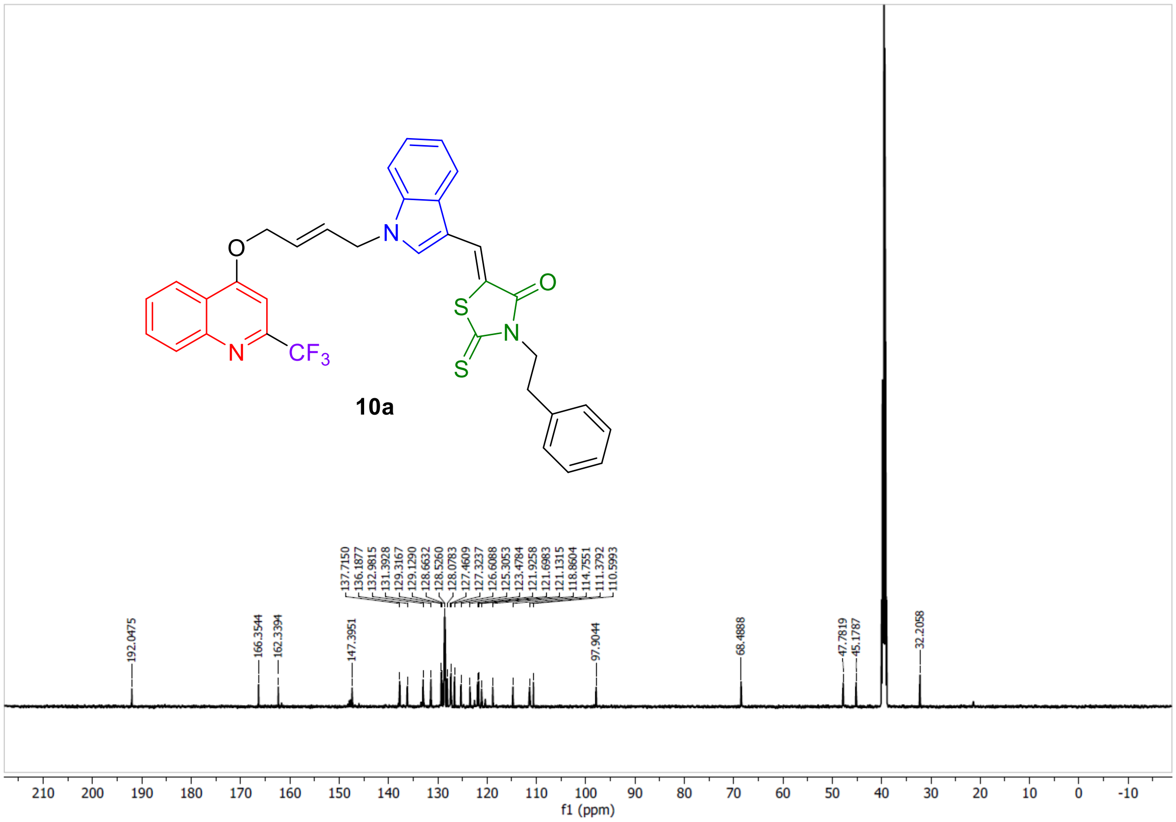
O
N
N
CF3
S
S
O
N
10a
192.0475
166.3544 162.3394
147.3951
137.7150 136.1877 132.9815 131.3928 129.3167 129.1290 128.6632 128.5260 128.0783 127.4609 127.3237 126.6088 125.3053 123.4784 121.9258 121.6983 121.1315 118.8604 114.7551 111.3792 110.5993
97.9044
68.4888
47.7819 45.1787
32.2058
210 200 190 180 170 160 150 140 130 120 110 100 90 80 70 60 50 40 30 20 10 0 -10
f1 (ppm)

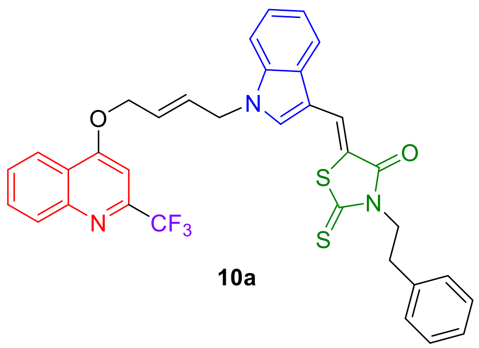
O
N
N
CF3
S
N
O
S

10a

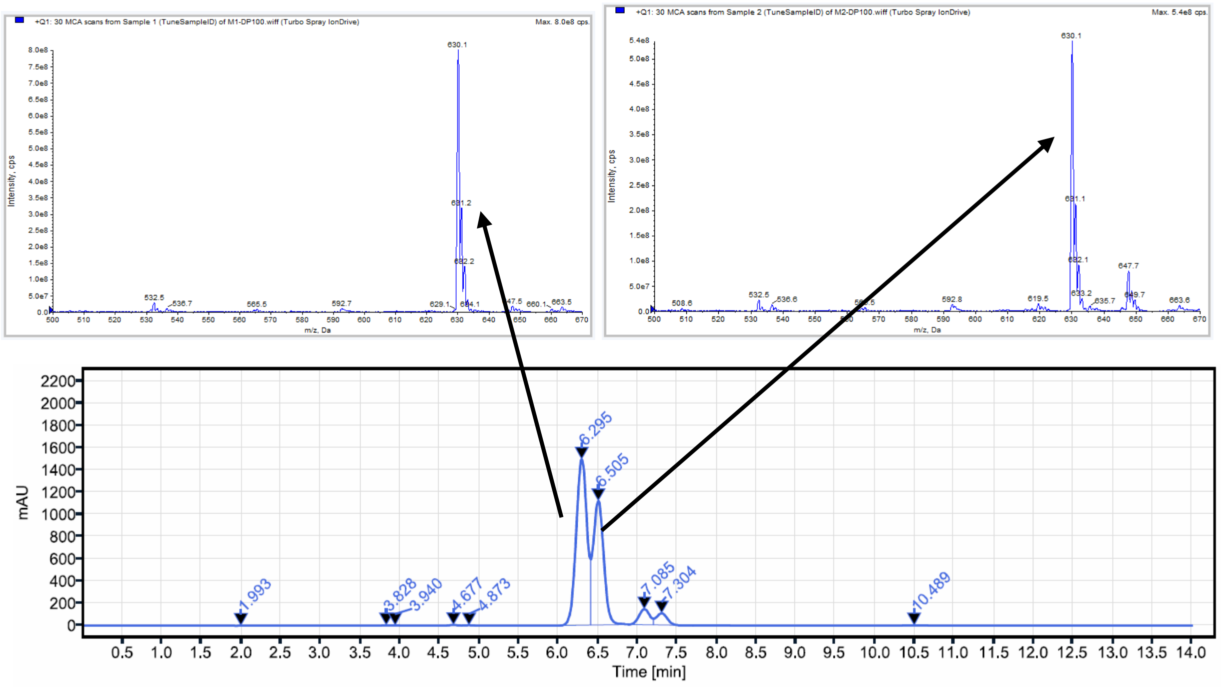
mAU
Time [min]
6.295
6.505
7.085
7.304
1.993
3.828
3.940
4.677
4.873
10.489
630.1
647.7

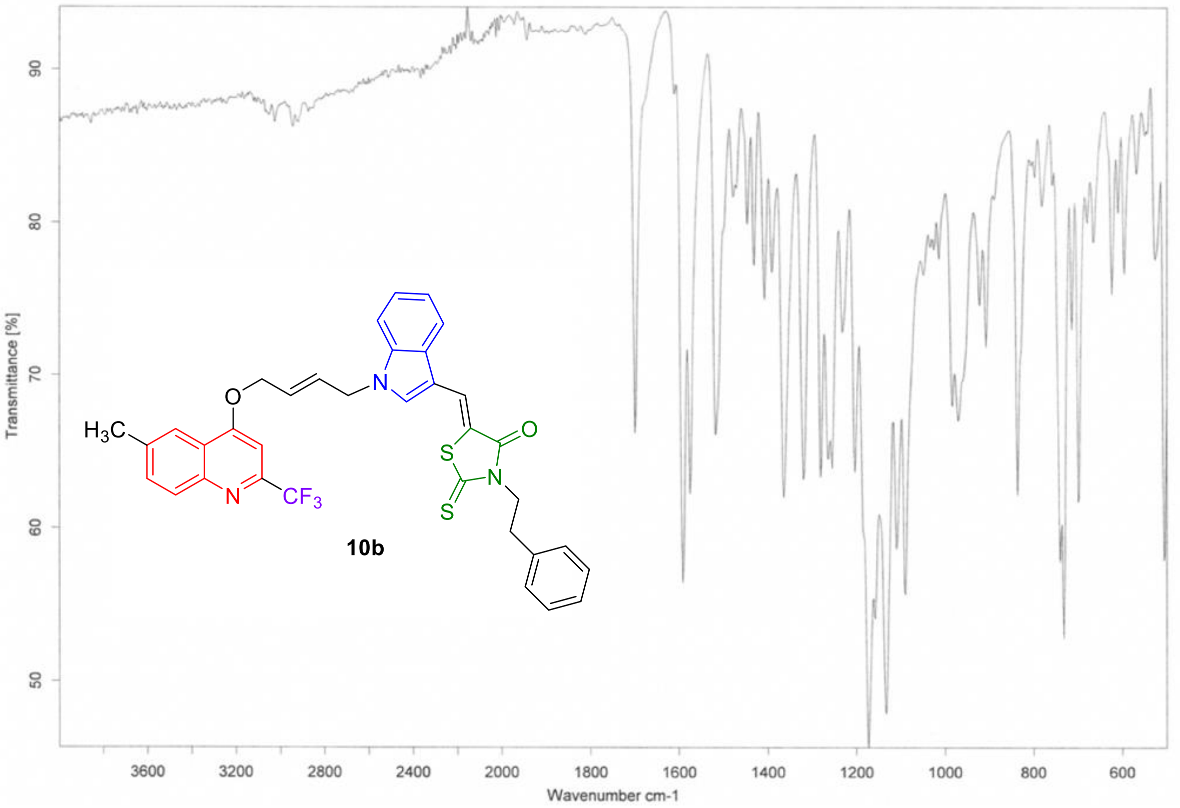
Transmittance [%]
Wavenumber cm-1
90
80
70
60
50
3600
3200
2800
2400
2000
1800
1600
1400
1200
1000
800
600
H3C
O
N
CF3
N
S
O
N
S
10b

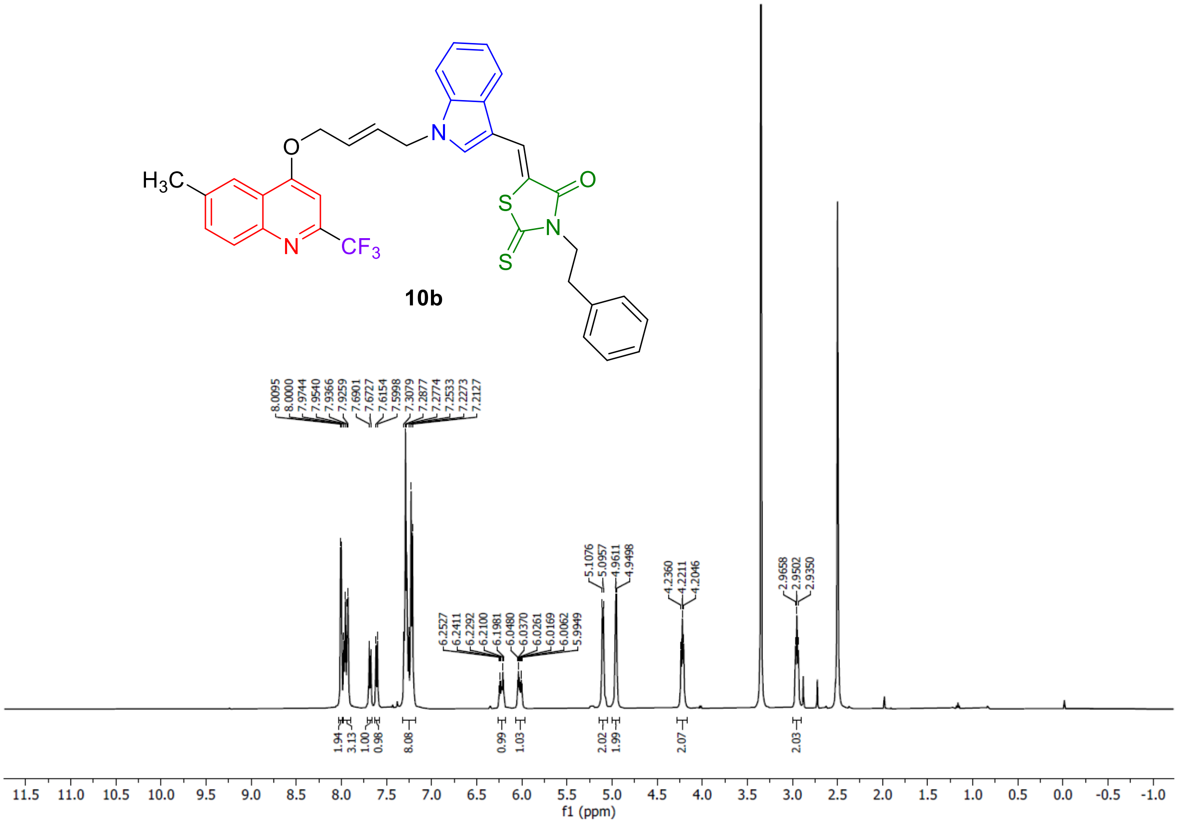

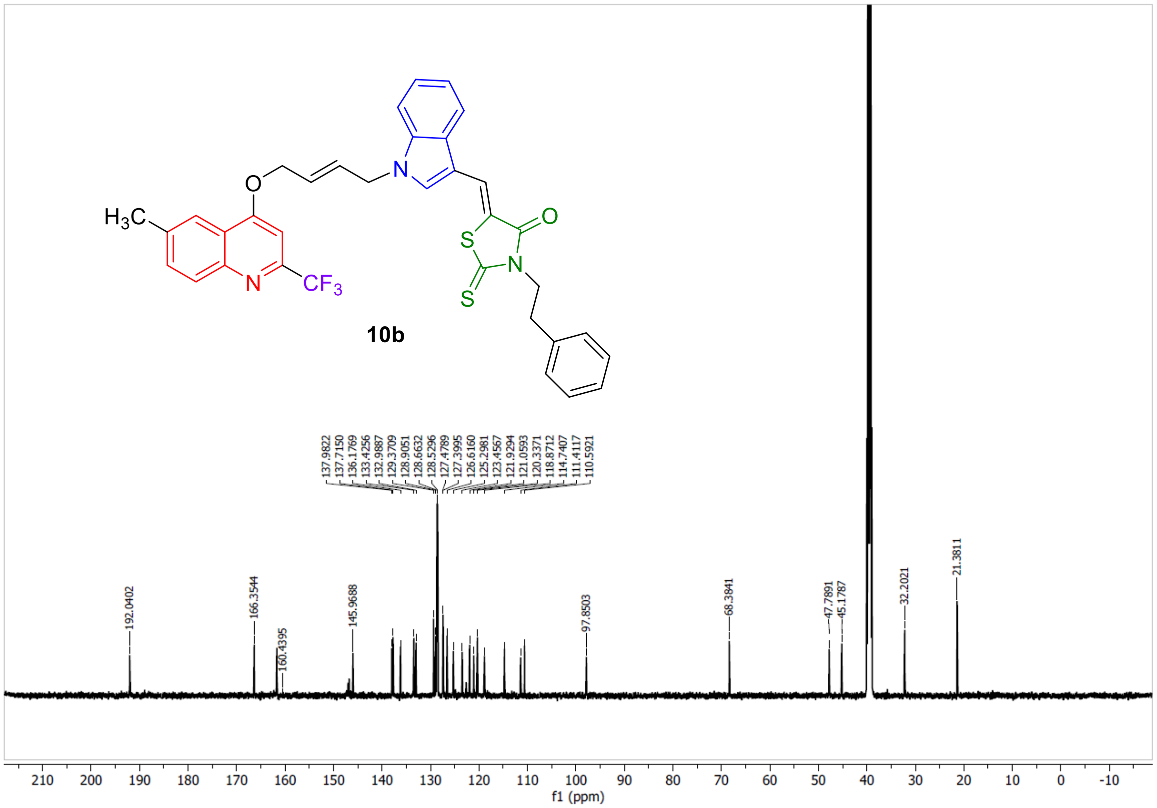

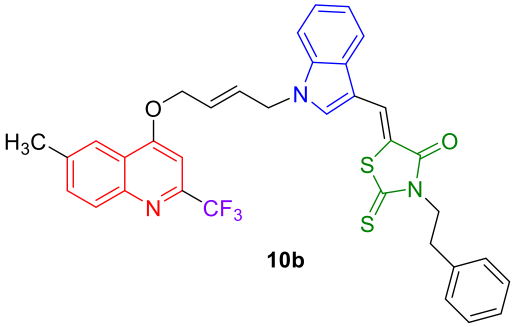

O
H3C
N
CF3
N
S
O
N
S

10b

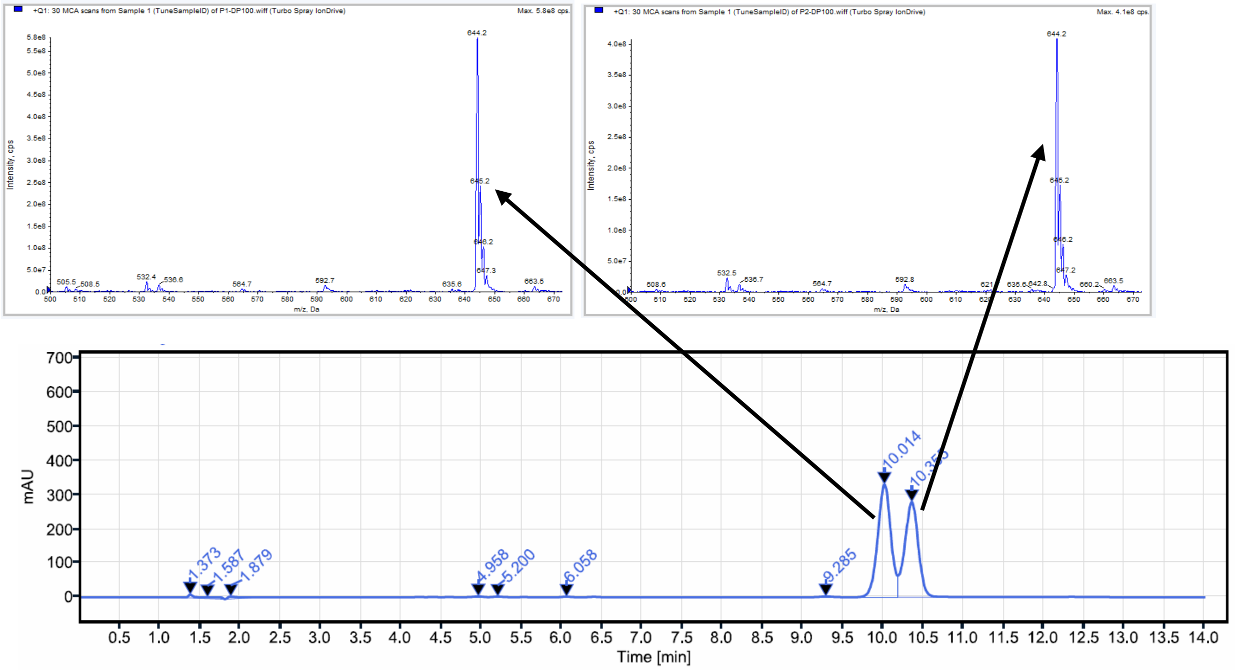

644.2
645.2
646.2
647.3
644.2
645.2
mAU
Time [min]
10.014
9.285
6.058
5.200
4.958
1.879
1.587
1.373
